# Global Suicide Mortality Rates (2000–2019): Clustering, Themes, and Causes Analyzed through Machine Learning and Bibliographic Data

**DOI:** 10.3390/ijerph21091202

**Published:** 2024-09-10

**Authors:** Erinija Pranckeviciene, Judita Kasperiuniene

**Affiliations:** 1 Faculty of Informatics, Vytautas Magnus University, LT-53361 Akademija Kauno r., Lithuania; 2 Faculty of Medicine, Vilnius University, LT-08661 Vilnius, Lithuania; 3 Education Research Institute, Vytautas Magnus University, LT-44248 Kaunas, Lithuania

**Keywords:** suicide, machine learning, keyword clustering, network analysis, association rule mining, text mining, word embedding, recurrent neural network, age-adjusted suicide mortality rate, bibliographic analysis

## Abstract

Suicide research is directed at understanding social, economic, and biological causes of suicide thoughts and behaviors. (1) Background: Worldwide, certain countries have high suicide mortality rates (SMRs) compared to others. Age-standardized suicide mortality rates (SMRs) published by the World Health Organization (WHO) plus numerous bibliographic records of the Web of Science (WoS) database provide resources to understand these disparities between countries and regions. (2) Methods: Hierarchical clustering was applied to age-standardized suicide mortality rates per 100,000 population from 2000–2019. Keywords of country-specific suicide-related publications collected from WoS were analyzed by network and association rule mining. Keyword embedding was carried out using a recurrent neural network. (3) Results: Countries with similar SMR trends formed naturally distinct groups of high, medium, and low suicide mortality rates. Major themes in suicide research worldwide are depression, mental disorders, youth suicide, euthanasia, hopelessness, loneliness, unemployment, and drugs. Prominent themes differentiating countries and regions include: alcohol in post-Soviet countries; HIV/AIDS in Sub-Saharan Africa, war veterans and PTSD in the Middle East, students in East Asia, and many others. (4) Conclusion: Countries naturally group into high, medium, and low SMR categories characterized by different keyword-informed themes. The compiled dataset and presented methodology enable enrichment of analytical results by bibliographic data where observed results are difficult to interpret.

## 1. Introduction

The suicide mortality rate (SMR) worldwide from 2000–2019 diminished in the majority of countries. However, in some countries such as the US, Mexico, Brazil, Paraguay, and others, the SMR was increasing. These trends are evidenced by the statistical crude and age-standardized suicide mortality rate data available from the World Health Organization (WHO) [1] and also World Bank World Development Indicator (WDI) SMR data [2]. The period of 2000–2019 was before the COVID-19 pandemic that might have affected the SMRs and interpretations.

The World Health Organization indicates [3] that more than 700,000 people per year kill themselves. But even more attempt it [4,5,6,7]. In particular, suicide among young populations [8,9,10] has become a global public health concern. A reduction of suicide mortality rate by one third by 2030 is one of the global goals of the WHO Member States.

There are multitudes of publications addressing this type of preventable death in various contexts. For the current study we collected 19,000 bibliographic records published from 2000–2019 having a suicide keyword either in the keywords or article title or abstract. This number of records shows the magnitude of research devoted to this field and efforts to develop prevention strategies [11,12,13]. It would be impossible to review and cite all publications. However, through the analysis of keywords it is possible to understand directions that are the focus of suicide research.

Epidemiological studies show that mental disorders, depression, and addictions are major risk factors of suicide [4,14,15]. Socioeconomic factors, unemployment rates, access to the internet, unhealthy lifestyle, and mental illness in urbanized areas all negatively affect suicide mortality rates [16,17,18] while increased literacy is strongly positively correlated with help seeking and a lower risk of suicidal ideation [19,20]. Economic downturns, increasing loneliness, and sleep disturbances are other factors increasing suicide rates [12,21,22] while restriction of access to the lethal means had driven concurrent fall in SMR worldwide [23]

Studies performed during various periods of time in different groups of countries reveal significant regional disparities in suicide mortality rates [24,25]. The incidence of suicide varies more than ten-fold between countries, being especially high in Eastern Europe and very low in the Middle East [13,24]. This disparity of suicide incidence between countries and regions is very interesting and merits more study.

Studying the differences and similarities of suicide incidence dynamics (SMR) and literature between countries may provide a better understanding of what makes some societies more prone or protective regarding suicide. Country- or region-specific cultural identity or geography may influence either susceptibility or resilience to suicide. Factors associated with suicidal ideation may have different causes in different regions and countries and may need to be addressed differently through prevention strategies. Reasons leading to the development of depression risk factors might be different in different regions. For example, unemployment in European or East Asian countries may be a serious risk factor for depression but not in Sub-Saharan Africa. In Sub-Saharan Africa, having an infectious disease may be a bigger problem and a risk factor for depression than in Europe.

We attempted to identify and systematically describe groups of countries similar in their SMR dynamics across different geographical regions: Europe and Central Asia, East Asia and the Pacific, South Asia, the Middle East and North Africa, Sub-Saharan Africa, North America, and Latin America and the Caribbean. To do so we first used unsupervised machine learning to group countries by similarity in their SMR dynamics from 200–2019. Subsequently, we performed analysis of keywords using the Web of Science database bibliographic records on articles published from 2000–2019 associated with suicide and each particular country.

The main goal of this study was to highlight common and specific suicide-related contexts in groups of countries similar in their SMR dynamics across geographic regions. We believe that the analysis and data collected by our study will be useful for practitioners developing suicide prevention strategies and ways to increase literacy.

## 2. Materials and Methods

### 2.1. Suicide Mortality Rate Dataset

The World Health Organization age-standardized suicide mortality rate per 100,000 population for the period 2000–2019 of 183 countries with no missing values was downloaded for this study [1]. SMR analysis between regions and in time has to be age-standardized since age structures can vary significantly between populations. Over time, the age composition of populations changes due to factors such as aging, birth rates, and migration. Age standardization allows for accurate tracking of mortality trends over time, distinguishing true changes in mortality risk from changes in population age structure and allows unbiased comparisons of SMR between countries and regions. The downloaded dataset was prepared as SMR time series in which each row represents a country and each column represents year.gender (females as yyyy.f, males as yyyy.m, and both genders as yyyy.b) (see data availability statement for availability). Corresponding crude SMR analysis data related to this study are available from Zenodo [26] for reference.

### 2.2. Web of Science (WoS) Bibliographic Records and Keywords

Bibliographic records from the Web of Science bibliographic database [27] were available for download for 158 out of 183 countries in the WHO dataset. For each country from the WHO dataset a search was performed in WoS for articles published from 2000–2019 with the query “country name” and “suicide” for the topic, meaning that both words must appear in the title or keywords or abstract. Full bibliographic records were downloaded for each country. The extended and author keywords were extracted and saved in separate files for each country/article, resulting in a training dataset of keywords in which each article is represented by a separate file containing one line of text with keywords separated by spaces and labeled by the country name (available from [28]). Some keywords are phrases, for example, “children left behind”.

### 2.3. Clustering

**Figure 1:**
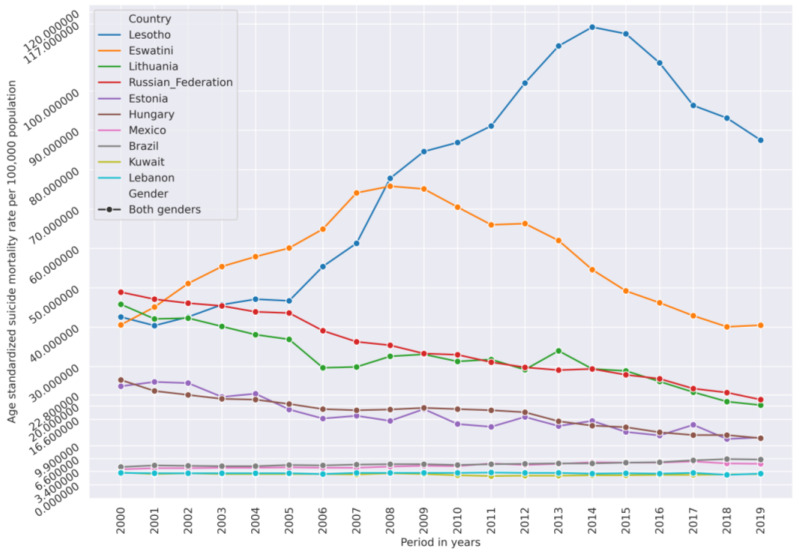
Age-standardized suicide mortality rate (SMR) in selected countries from 2000–2019 showing similarity of SMR levels and trends in selected countries: Estonia is similar to Hungary, Lithuania is similar to Russian Federation, Brazil is similar to Mexico, and Lebanon is similar to Kuwait. The lower y-axis ticks represent the 10th (3.4), the 30th (6.6), the 50th (9.9) (median), the 80th (16.6), and the 90th (22.8) percentiles of the SMR across all countries. Some countries, such as Lesotho and Eswatini, have an SMR in most of the years from 2009–2019 which is considered high compared to other countries. On the other hand, the countries in which SMR in most of the years from 2000–2019 was below the value of the 10th or 20th percentile can be considered low-SMR countries compared to the other countries.

Groups of certain countries have very similar suicide mortality rate (SMR) dynamic trends from 2000 to 2019 which allowed us to use unsupervised agglomerative clustering to cluster their SMR time series. Figure 1 represents age-standardized SMRs per 100,000 population time series of both genders in different countries. This figure is to illustrate the similarity of SMR dynamics in some countries.

The suicide mortality rate time series for each country comprising rates of both genders from 2000–2019 were clustered using hierarchical clustering with Euclidean distance and Ward linkage as implemented in the Orange data mining tool [29]. The rationale for this choice is that Euclidean distance metric is widely used to measure time series similarity. If the time series are very similar in magnitude and trend, then there will be a small distance between them in the Euclidean sense. In Ward linkage in the agglomerative clustering procedure, a pair of clusters is chosen to merge such that there is a minimum increase in total within-cluster variance after the merge. In this way, the most similar SMR time series will be grouped together in the initial stages of the algorithm. Grouping at the lowest level naturally will put together SMR time series that have very similar SMR magnitude and dynamics.

Agglomerative clustering does not determine the number of clusters automatically. An optimal number of clusters is determined from the length of dendrogram branches that represent distances between the clusters. The resulting clustering was assessed by silhouette scores measuring how well a country belongs to its cluster. Different similarity metrics and linkage algorithms were attempted. The agglomerative hierarchical clustering using Euclidean distance and Ward linkage produced the best subdivision of countries into clusters with the highest silhouette scores.

### 2.4. Quantification of Countries into High-, Medium-, and Low-Suicide-Mortality-Rate Groups

No hard threshold exists in determining what is considered a high, medium, or low SMR. This categorization has to be performed using a data-driven comparative approach. In our study, we labeled a country as having a low, medium, or high SMR based on percentiles of age-standardized suicide mortality rate values available for all 183 countries and all years from 2000–2019. We computed each 10th percentile from all SMR values and established SMR intervals spanning from low to high. Table 1 shows intervals and interpretations for each bin.

Regarding intervals, 30% of SMR values are placed into low (low, low medium, and Low high) intervals, 40% of SMR values into medium (ML, M, M, and MH) intervals, and 30% of SMR values into high (HL, HM, and H) intervals. Although this subdivision of SMR is somewhat arbitrary and fixed for the years 2000–2019, it helps to generalize based on the statistical properties of the dataset. Having established intervals, each country is quantified as having a low, medium, or high SMR by counting how many years were in each SMR bin. The final category of country SMR is assigned based on the counts in each group of intervals: high, medium, or low. Country quantification into categories was performed by in a semi-supervised way using curation. A quantification example for Estonia (SMR is shown in Figure 1) is presented in Table 2.

Estonia’s SMR over the period of 20 years was high for 16 of them. Therefore, Estonia can be labeled as a high-SMR country compared to other countries. If a country does not have a very clear unimodal distribution of SMR, such as Estonia, then it is given a composite label of major modes of the SMR distribution. Table 3 represents an example for Qatar.

Qatar’s SMR was in the medium low (ML) interval eight times, in the low medium (LM) interval six times, and in the low high (LH) interval six times, fluctuating time on the boundary between medium and low. Therefore, Qatar can be labeled as a medium-low-SMR country. If SMR spans all low, medium, and high intervals, which is rare, then that country is assumed to have a medium wide-spectrum SMR. See the data availability statement for the quantification of SMR of all countries. Clusters obtained in the agglomerative clustering procedure carry the label of the majority of countries. The labeling is rather qualitative, however, it helps to generalize and assess each country’s SMR with respect to the other countries.

### 2.5. Analysis of Keyword Co-Occurrences Using VOSviewer

Visualization of Similarities viewer (VOSviewer) is a software package used to visualize bibliometric networks. It can be used to find groupings of items linked together by frequent co-occurrences in a subset of articles [30,31]. We analyzed networks of frequent keyword co-occurrences of WoS records to identify semantically distinct leading themes originating from the network of semantically linked keywords in each cluster of countries. Distinction between the themes is achieved by Louvain clustering of network nodes (keywords) by optimizing modularity of the network where a module is a strongly interconnected group of keywords within a group separate from other groups. These modules of keywords highlight different dominant topics existing in analyzed records. Aiming to restrict the number of keywords to a perceptually manageable size, for each analyzed cluster only 35 to 50 top linked keywords were selected to derive the dominant semantically differing topics.

Dominant topics were identified for clusters of high-, medium-, and low-SMR countries in each geographic region, taking only the most significant 5 to 10 representative keywords describing each dominant topic (cluster in VOSviewer terminology). The significance of the keyword in the dominant topic was determined by the frequency of occurrence of this keyword and the number of total links that it formed with other keywords. General, not informative, keywords such as suicide, mortality, risk, epidemiology, prevalence, prevention, health, behavior, suicidal risk, ideation, and similar were omitted. The chosen keywords were used as keyword annotations of high-, medium-, and low-SMR country clusters in tables summarizing countries in each geographical region.

### 2.6. Association Rule Mining

The association rule mining method identifies item sets frequently occurring together in the records. This method is widely used to analyze market transactions data in order to derive rules informing about customer buying preferences. It can be applied to any type of record of items including keywords of sets of articles. We used the Python Mlxtend library to perform association rule mining of frequent keyword sets [32].

### 2.7. Network Visualization

Cytoscape software [33] was used to visualize and analyze relationships between frequent keywords in sets of articles associated with geographic regions.

### 2.8. Deep Learning

Analysis of word neighborhoods using proximity between the word embedding vectors is a very useful technique in exploring and understanding semantic relationships between subsets of words representing specific contexts. The target context of this study was suicide. A recurrent neural network with a word embedding layer was trained to discriminate between 158 countries utilizing TensorFlow and Keras deep learning libraries [34] (pp. 309–363). A single training data point consisted of the keywords assigned to a single article. Each country that had at least one associated article was a class, and in total there were 158 countries/classes that had at least one associated article. The word embedding layer was set to 300 dimensions (used as default in most state-of-the-art word embedding learning algorithms). Training data preprocessing consisted of the creation of a vocabulary including all keywords and conversion of keywords into a numerical representation using their indexes in the vocabulary. A bidirectional long short-term memory (LSTM) layer was used in the network to learn from keyword sequences. The intended use of this network was to derive word embedding vectors that can be explored further including available analysis tools in the TensorFlow embedding projector [35].

### 2.9. Data Analytics Software

Data analysis and visualizations were carried out by the Orange data mining tool v 3.36.2 [29] and custom Python scripts.

## 3. Results

### 3.1. Summary Statistics of Suicide Mortality Rates

Summary statistics of age-standardized suicide mortality rate (SMR) in all 183 countries of all years during the period 2000–2019 show its main intervals of variation. The overall summary statistics for males and females and both genders in all countries and all years from 2000 to 2019 are presented in Table 4.

The 3rd quartile of the SMR from 2000–2019 shows that for both genders it was below 14.9 per 100,000 and this value can be considered as a conditional threshold separating low and medium SMR from high SMR during that time period. The summary statistics also indicate that the SMR in females is almost three-fold lower than the SMR in males overall during that period. The fact that females have a much lower SMR than males is already known.

### 3.2. Clustering of Countries into Groups of Similar SMR Magnitude and Dynamics

**Figure 2:**
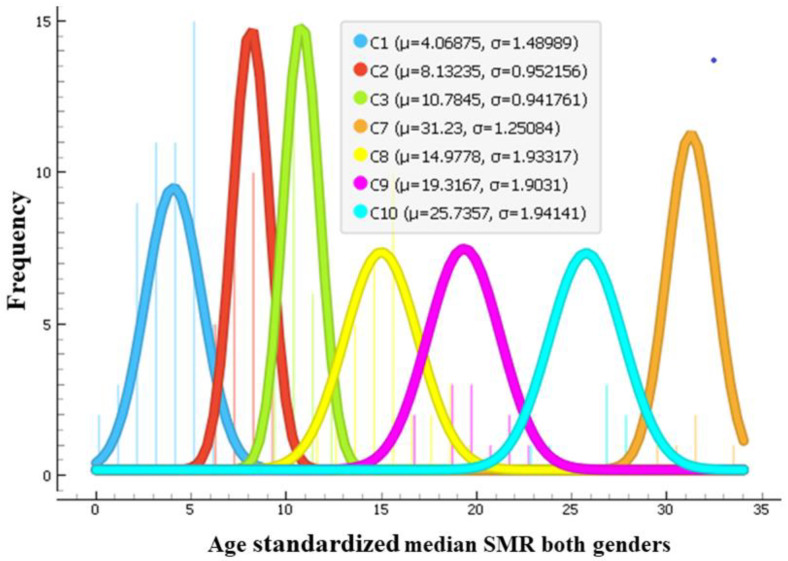
Histograms approximated by normal distribution of age-standardized median SMR values in countries from 2000–2019 in each cluster. Clusters with one or two elements are omitted. Legend shows parameters of the approximation by normal distribution—values of the centroids of median SMR of the countries grouped into each cluster and a standard deviation.

Using age-standardized SMR time series from 2000–2019 of both genders as feature vectors, the 183 countries were clustered by an unsupervised agglomerative hierarchical Ward linkage clustering procedure with Euclidean distance as similarity as implemented in the Orange data mining tool. Based on the height of dendrogram branches, the 10 clusters seemed optimal, since increasing a number of clusters beyond 10 produced many clusters containing a single element. The cluster dendrogram is presented in Supplementary Figure S1. Time series of SMRs in Lesotho and Eswatini (also known as Swaziland) were very different from the rest (see Figure 1). These countries formed clusters of one element. Guyana and Kiribati also formed a cluster of two elements. The median silhouette score of the clustering is 0.435, 1st quartile is 0.257, and 3rd quartile is 0.528, which indicates a fair clustering. However, 12 countries had low scores. Clusters with smaller number of elements were more homogeneous. Clusters with a high number of countries were more variable. Countries close on the dendrogram had very close SMR dynamics, but similarity decreased with the distance between countries on the dendrogram within the assigned cluster. The cluster silhouette plot is presented in Supplementary Figure S2. Nevertheless, the resulting clusters comprised countries generally similar by their SMR magnitude and dynamics from 2000–2019. The median values of age-standardized SMR from 2000–2019 of each country were calculated. Figure 2 shows histograms of those median values in each cluster (except clusters with one or two elements). These histograms are approximated by the normal distribution. In this way, Figure 2 illustrates how far apart the cluster centroids are by the magnitude of median SMRs of countries grouped into these clusters.

**Figure 3:**
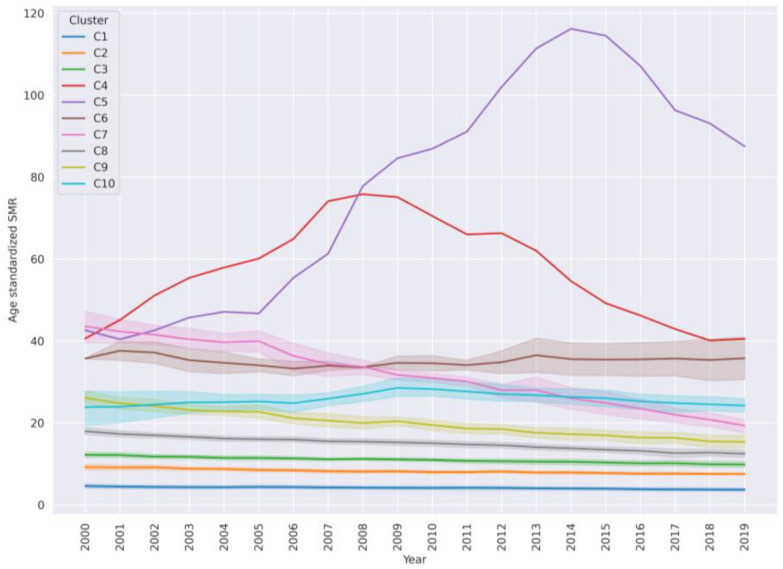
Overall average trends of suicide mortality rate dynamics (with confidence intervals) in each cluster. Cluster C4 is Eswatini, cluster C5 is Lesotho and cluster C6 contains Kiribati and Guyana. Other clusters have at least 5 countries.

In order to discuss clusters in a generalized manner, countries from clusters were labeled as low, medium, or high (or composite) SMR as described in the Materials and Methods. The dynamic of SMR in clusters from 2000–2019 is represented by Figure 3. For each cluster the mean SMR of countries in the cluster is represented by a line. The shadow around the mean represents the confidence interval for the SMR values for each year from 2000–2019. Very-high-SMR clusters are: Eswatini—C4, Lesotho—C5, Kiribati and Guyana—C6, Belarus, Lithuania, Russian Federation, Kazakhstan, and Botswana—C7. High-SMR clusters are C9, comprising 12 countries, and C10, comprising 7 countries. The rest are low- and medium-SMR country clusters. Cluster C8 with 36 countries is in between a high and medium SMR. In this cluster, the SMR was variable during the period 2000–2019. Cluster C2 with 34 countries and C3 with 29 countries fall into the medium-SMR interval with a total median SMR ~10 (Table 4). Finally, cluster C1 with 56 countries comprises low-SMR countries.

In the majority of countries, the SMR over the years is trending downwards. In high-SMR countries (C7 and C9), the downward slope is steep and the SMR changes are quite synchronous. These clusters have many countries from the former USSR. During the first decade from 2000–2010 the SMR overall was higher than in the following decade in many countries. The code to plot and explore these trends is available as a Jupyter notebook [28]. In the following, each cluster will be discussed in turn.

### 3.3. Characteristics of High-, Medium-, and Low-SMR Country Clusters

#### 3.3.1. High- and Mixed-High-and-Medium-Suicide-Mortality-Rate Country Clusters

**Figure 4:**
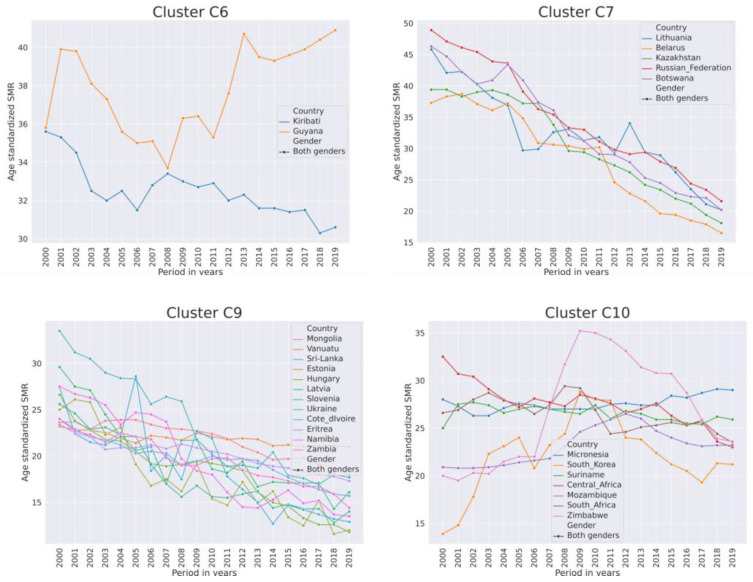
Trends of SMR per 100,000 population of both genders from 2000–2019 in high-SMR country clusters C6, C7, C9, C10.

High-SMR country clusters have a small size compared to other clusters. Clusters subdivided by geographical region are shown in Table 5. The majority of high-SMR countries from 2000–2019 had an SMR above the 3rd quartile (14.9) of the total SMR. The trends in SMR in high-SMR country clusters are shown in Figure 4. Countries with the highest SMR were from the former USSR and Sub-Saharan Africa. Overall, SMR was declining, while it was fluctuating in African countries and it was increasing in Guyana. In African countries and South Korea, the SMR had increasing trend from 2007–2009. Out of 24 high-SMR countries, 19 are the least developed and developing regions (United Nations list). In countries from C7, the SMR was declining more rapidly than in countries from C9.

In Guyana, which had a very high SMR trending upwards, family dysfunction, child maltreatment, and alcoholism were identified as factors affecting the SMR [36].

Another group of countries that had an SMR between high and medium (cluster C8) is presented in Table 6. This group comprises 36 countries, 20 of which are Sub-Saharan Africa countries.

**Figure 5:**
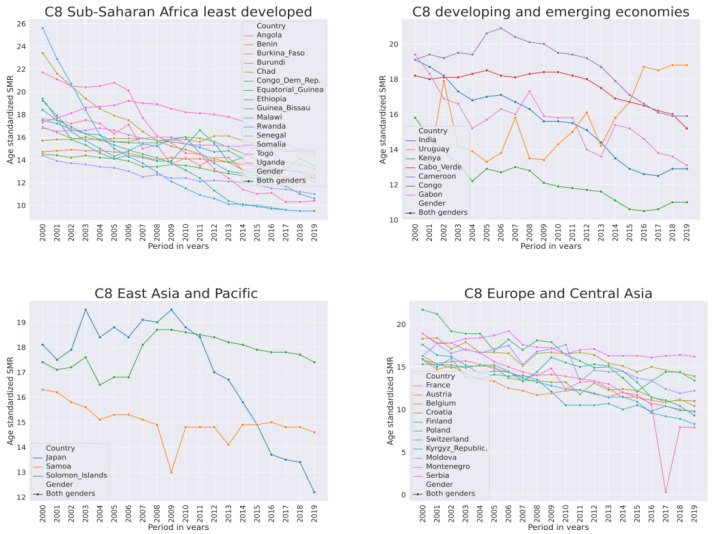
Dynamics of suicide mortality rate per 100,000 population from 2000–2019 for both genders of countries in cluster C8 that had a mix of high and medium SMR.

Countries from Sub-Saharan Africa in this cluster are from the least developed (United Nations list) or developing regions. European countries in this cluster are developed economies and other emerging economies. The SMR dynamics in countries of this cluster are shown in Figure 5.

The least developed and developed European countries in this cluster had similar magnitudes of SMR. In these countries, the SMR was declining from 2000–2019. The decline was more rapid in Sub-Saharan countries than in European ones. In Sub-Saharan Africa, the most rapid decline was in Rwanda and Uganda from an SMR of 26 and 22 in 2000 to 10 in 2019. Of all countries, only in Uruguay did the SMR rise (Figure 5 top right panel “C8 developing and emerging economies”). A quick review of the literature associated with African countries revealed frequent themes of community support, women’s health, terrorism, and psychological autopsy. Psychological autopsy is a suicide research method and was mostly associated with Uganda. Another quick review of the literature associated with developed European countries revealed euthanasia as a leading theme.

#### 3.3.2. Medium-SMR Country Clusters

Medium-SMR countries comprise a total of 63 countries in two clusters: C2 and C3, in which C3 is characterized by a higher SMR. Medium-SMR countries from C2 and C3 are shown in Table 7. Medium-SMR countries are the largest group and are not localized in any geographical region. In this group, there are 14 countries from Sub-Saharan Africa and countries from South and East Asia that were in war conflicts.

**Figure 6:**
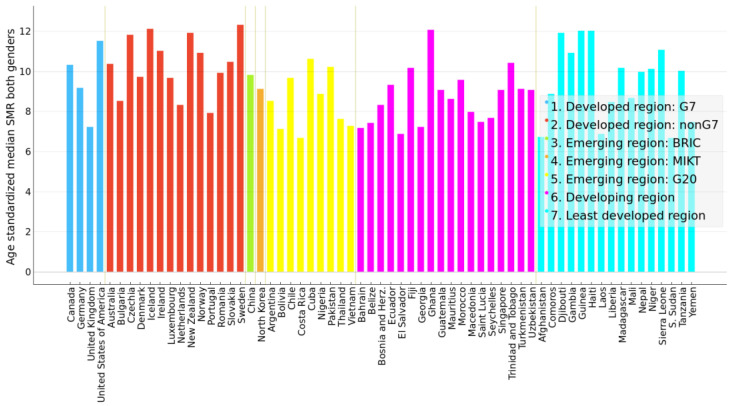
Bar plot of median suicide mortality rate per 100,000 population of years 2000–2019 for both genders marked by economy for medium-SMR countries in clusters C2 and C3.

**Figure 7:**
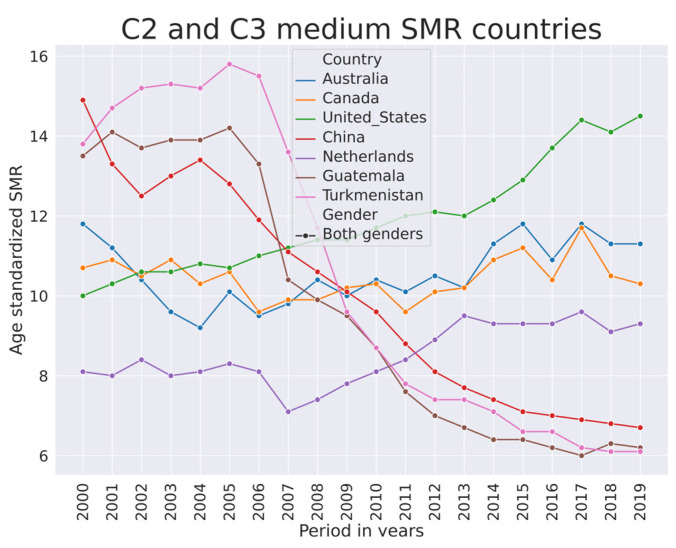
SMR trends in selected countries from clusters C2 and C3.

This group of countries is also a mix of economies, as shown in Figure 6. There does not seem to be a correspondence between economy and magnitude of SMR in this group. While more high-SMR countries had the least developed and developing economies, in the medium-SMR group all economies are represented rather proportionally. Figure 7 shows SMR trends in selected countries.

The median SMR from 2000–2019 is higher in European developed economies. Overall, the SMR is trending down in this group of countries as well. The Netherlands (Figure 7) and Portugal (data in [1]) have fluctuating SMR that did not decline as rapidly as in other European developed countries. In Australia and Canada, the SMR was trending downwards from 2002–2012, but afterwards it started trending upwards. In the US, the SMR was increasing. In China, it was rapidly decreasing. Figure 7 also depicts SMR dynamics in Turkmenistan and Guatemala. This is an illustration of synchronicity of SMR in two countries that geographically are far apart.

In the literature associated with C2 and C3 countries, various heterogeneous topics emerged. Mostly, they were depression, mental disorders, addictions, euthanasia, euthanasia law, sedation, war, veterans, and unemployment.

#### 3.3.3. Low-Suicide-Mortality-Rate Country Clusters

Low-suicide-mortality-rate countries from cluster C1 are shown in Table 8. This is the largest single cluster comprising 56 countries.

**Figure 8:**
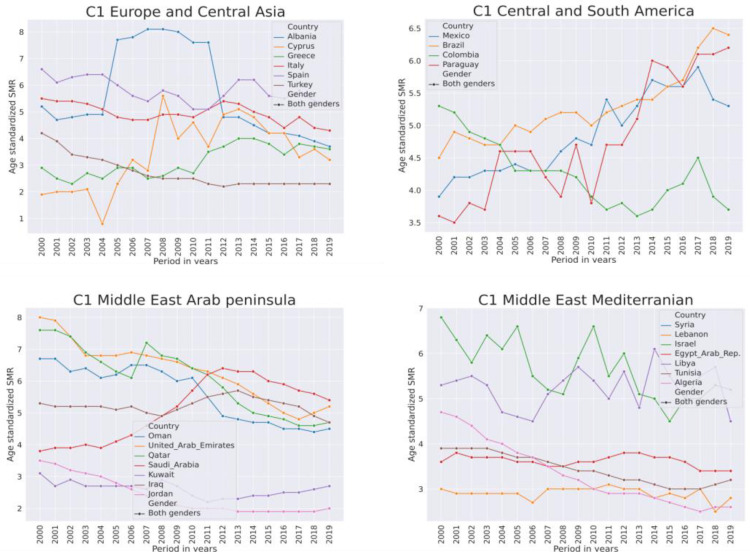
Trends of suicide mortality rate per 100,000 population in years 2000–2019 for both genders in low-SMR countries from cluster C1.

The largest proportion of countries in this group are from the Middle East and North Africa and also from Latin America and the Caribbean region. In the majority of countries, but not in all, in this cluster their SMR frrom 2000–2019 was below the first SMR quartile (SMR < 5.6). Countries in C1 from the Eurasian continent are situated around the Mediterranean Sea. Figure 8 shows SMR trends in several selected countries from C1.

In this cluster, the SMR trends in the developed European countries are stable but tended to increase in Greece. In the Middle East and North Africa, the SMR in general was declining. It was fluctuating in Israel and Libya. It jumped in Saudi Arabia from 2006–2011. In Central and South America, in general, the SMR was variable but declining. However, in the emerging economies of Brazil, Mexico, and Paraguay, the SMR is increasing. In South Asian and Sub-Saharan African regions, the SMR was decreasing in this group, except Bangladesh and Malaysia were trending up slightly in the last years. In the literature associated with low-SMR countries the most frequent topic was war.

#### 3.3.4. Increasing SMR

**Figure 9:**
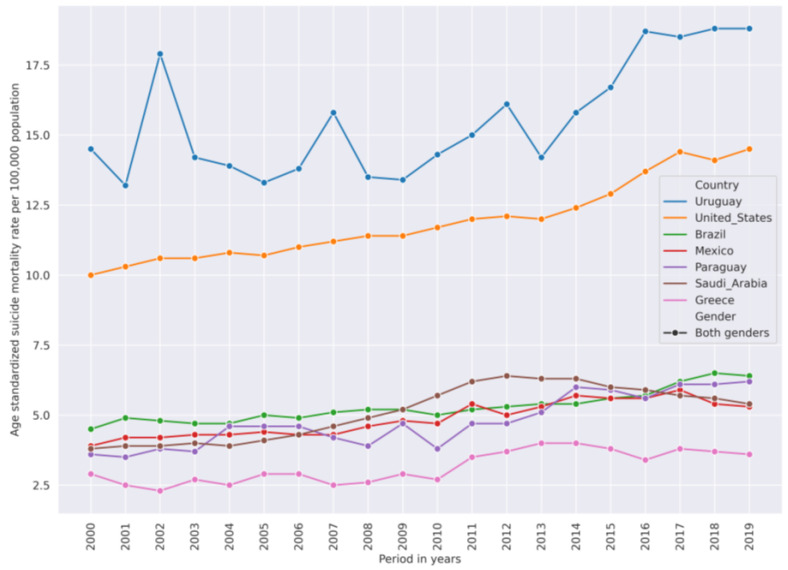
Countries that have increasing SMR trend from 2000–2019. Uruguay comes from a high-SMR cluster. United States is from a medium-SMR cluster. Other countries are from a low-SMR cluster.

The overall suicide mortality rate was declining from 2000–2019 but not in all countries. In the US, the SMR was going up: in 2000 it was 10 per 100,000 population, but in 2019 it increased up to 14. Figure 9 shows countries with increasing SMR.

### 3.4. Results of Association Rule Mining

A quick analysis of the literature associated with the countries in high-, medium-, and low-SMR groups showed similar themes repeating in all groups of countries. We could not discern specific topics in suicide research attributable to the groups of countries or regions without a more detailed analysis. We further investigated themes frequent across geographical regions by performing association rule mining on keywords in articles associated with individual countries in each geographic region.

**Figure 10:**
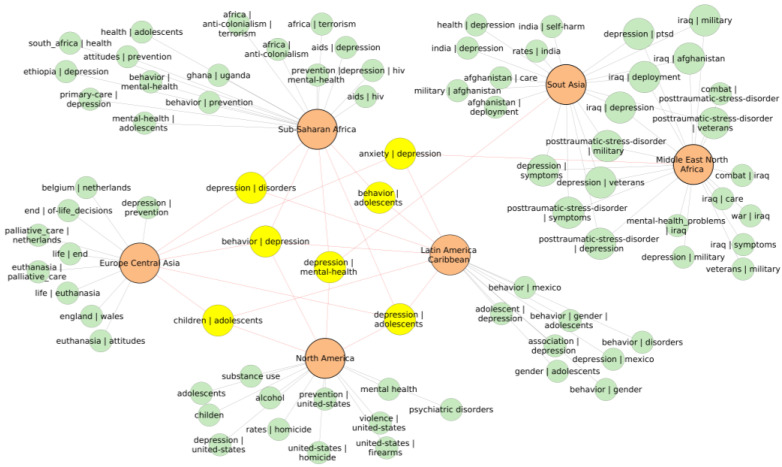
Network representation of the top 15 most frequent keywords and phrases (item sets) resulting from association rule mining algorithm applied on keywords of articles associated with countries in each geographical region. Highlighted are the most frequent phrases common between the regions. Depression emerges as the central theme in all regions.

Association rule mining helps to identify items frequently occurring together in a collection of records. Mostly, it is used in marketing to identify products frequently bought together [32]. Here, the record was represented by a list of keywords associated with one article associated with one country. Collections of the records consisted of records of all countries in the particular geographic region. Frequent combinations of phrases were extracted from the collections of the records. The top 15 phrase combinations with highest support were selected. Values of support were different for each region since they depend on the number of records. Therefore, the selection was based on a fixed number of the top-ranking phrase combinations. From the selected phrases, the repetitive and uninformative frequent phrases containing suicide, mortality, prevalence, and similar were filtered out. From the selected phrases, a network was created in which nodes consisted of regions and the phrases. The purpose was to identify and visualize themes connecting the regions and the specific themes. The simplified pruned network is shown in Figure 10. We limited the number of nodes to only the most frequent phrases to make the network manageable.

Overarching themes connecting all regions were depression and adolescents. The term “depression” frequently occurred with other phrases such as behavior, disorders, anxiety, mental health, and also adolescents. This analysis revealed contexts frequently researched in suicide literature together with depression.

Each geographical region, however, had its own very frequent specific themes. In Sub-Saharan Africa, the themes were HIV/AIDS, anticolonialism, terrorism, mental health, Ghana, Uganda, and Ethiopia. In Europe and Central Asia, the prevailing theme was euthanasia, Netherlands, and Belgium. In North America, the most frequent topics were related to substance use and violence. In Latin America and the Caribbean, the most frequent topics were adolescents and gender. In the Middle East, North Africa, and South Asia, the themes were related to war, military, and post-traumatic stress disorder. In South Asia, India and self-harm were frequent. Such simple analysis of frequent phrases in articles associated with suicide and a particular country allowed us to highlight themes that are common and also specific themes that gained more attention in different geographical regions. The themes identified by association rule mining were in good correspondence with themes found by VOSviewer that will be discussed in the following section.

Association rule mining revealed the most frequent and common themes of interest in the context of suicide in different regions. The source for this analysis was a textual corpus of keywords of ~24,000 expressions originating from bibliographic records of 19,000 articles. Frequent combinations of phrases revealed a few dominant themes associated with each geographic region. To refine these results in groups of high-, medium-, and low-SMR countries in a geographic region, we analyzed subsets of bibliographic records associated with those groups of countries using the VOSviewer tool. In addition, we explored frequent keyword occurrences repeating across multiple countries in the group as well as frequent keywords specific to only one particular country.

### 3.5. Descriptive Analysis of High-, Medium-, and Low-SMR Country Clusters Across Geographic Regions Using Visualization of Similarities Viewer (VOSviewer) Tool and Frequent Keyword Occurrences

To perform VOSviewer analysis [30] we used Web of Science database bibliographic records of 19,000 articles published from 2000–2019. Selection criteria were such that the term suicide and a country name had to be in the title or keywords or abstract. Among those selected, the US had the most associated articles published during that period—3563. Ireland was in second place—1258 articles. In third place was Australia—1070 articles. Other countries that had many associated articles were China—896, Japan—770, Canada—730, the Netherlands—639, India—570, Sweden—558, and Germany—462 articles. The number of associated articles does not correlate with the suicide mortality rate across countries. However, European countries were in a higher suicide mortality rate bracket compared to Middle Eastern countries and, statistically, had more associated articles compared to the low-SMR Middle Eastern countries.

In this section, we summarize details about each geographic region with regard to groups of high-, medium-, and low-SMR countries in this region. We annotated clusters of countries using top keywords inferred by VOSviewer and reviewed and summarized the most frequent keywords repeating across multiple countries in the group. For the most part, keywords repeating across many countries agree with the top keyword annotations derived by VOSviewer. In addition, we summarized country-specific keywords that highlight uniqueness.

#### 3.5.1. Europe and Central Asia

There are 48 countries from the Europe and Central Asia region listed in Table 9. For each country, the first, median, and third quartiles of SMR, the SMR category, the number of bibliographic records associated with the country, and top keyword annotations derived by VOSviewer are presented in the table. Countries from the Europe and Central Asia region predominantly have medium and high SMR except for Turkey, Albania, Cyprus, Greece, Italy, and Spain. The low-SMR European countries are from the Mediterranean region. Western, Eastern, and Northern European countries had rather high SMRs. The former USSR had an especially high SMR compared to the rest. Even though it is decreasing, its magnitude was still above the third quartile of the total SMR of 14.9 per 100,000 population for both genders.

Frequent keywords associated with Europe and Central Asia point to several themes. The first are euthanasia and assisted suicide. Other frequent themes in many countries comprise mental disorders, parasuicide, and psychiatric disorders. Parasuicide is a form of self-harm in which someone mimics the act of suicide without the intent to kill (MedGen Concept ID: C0595861). Lastly, in many countries, frequent mentions are made to adverse childhood experiences, youth, and social support. Former USSR countries are marked by alcohol consumption and crime. Other keywords repeating across countries are unemployment, economic crisis, financial crisis, hopelessness, elderly, hospitalization, psychological distress, and substance use. There are mentions of seasonality and weather. Economic and financial crisis themes were most frequent in Greece. Euthanasia frequently occurred with the Netherlands and Belgium with no mentions among keywords associated with countries from cluster C7 (Table 9).

Some country-specific unique keywords in relation to suicide were as follows. *Belgium*—non-voluntary euthanasia and continuous palliative sedation; *Hungary*—congenital abnormalities and suicide attempt during pregnancy; *Slovenia*—heroin use and social and economic consequences of gambling; *Belarus*—alcohol psychosis; *Lithuania*—relation with authority and cosmic ray; *Russian Federation*—migrations, migratory politics, depopulation, programs for prevention of suicides among minors, native peoples of Siberia; *Ukraine*—strategy of eradicating female terrorism; *Austria*—rugged individualism, film effects; *Croatia*—heavy metals; *Finland*—global solar radiation, adolescent parricide; *Poland*—political protest, exile, Polish Catholicism, Satanism; *Serbia*—responsible media reporting, invisible victims, code of ethics of Serbian journalists, prison environment, plant toxins; *Switzerland*—suicide regulation, maintaining hope, unassisted suicide; *Bosnia and Herzegovina*—emo subculture (lifestyle based on emotional hardcore punk rock music); *Denmark*—life mission theory, shelters, radicalization awareness; *Germany*—inpatient suicide; dialectical behavioral therapy for adolescents; *Iceland*—friendship networks; *Ireland*—ganciclovir, planned complex suicide, Iowa gambling task, bystander effect; *Norway*—tension type headache; *Portugal*—water supply, children depression, drinking water lithium; *Romania*—intimate partner homicide suicide, children left behind; *Sweden*—international adoption; *Italy*—illicit drug overdose; *Netherlands*—train the trainer, e-learning; *United Kingdom*—genderqueer, political terrorism, pastoral care; *Turkey*—shelter, chronotype, authoritarian, incest, mobbing; *Armenia*—personality peculiarities; *Azerbaijan*—electronic device; *Cyprus*—workplace empowerment, colonial policies, goats; *Greece*—Greek crisis, marital quality, lunar periods, cyclicity, female aborigines.

#### 3.5.2. Sub-Saharan Africa

The Sub-Saharan Africa region has 48 countries that are shown in Table 10. Several African countries, such as Lesotho and Eswatini, had the highest SMR from 2000–2019 and no published articles in relation to suicide. Not many countries in this region had associated publications except of South Africa which had 232 publications. Keywords that did not appear in VOSviewer top keyword annotations but did appear in no less than five countries were anticolonialism and CIA, mentioned in articles associated with Guinea, Cameroon, Congo, Togo, Mozambique, Zimbabwe, and Kenya. In addition to these countries, terrorism was frequently mentioned in articles associated with Mali, Chad, Niger, Nigeria, Ghana, and Kenya. Women and HIV were frequent topics in almost all countries. Other frequent topics were nurses, students, education, disability, injury, alcohol, gender differences, social support, low income, domestic violence, sexual violence, men, and war. Less frequent were mentions of suicide bombing, Boko Haram (jihadist group), corporal punishment, and terms from virology, molecular biology, and plant toxicity.

Some unique country-specific keywords: *South Africa*—non-fatal suicidal behavior; *Zimbabwe*—lysosomes, protein crystal structures; *Ethiopia*—neglected tropical disease, non-filarial elephantiasis; *Ghana*—funeral rites, moral infraction, disabled women, sleep disruption; *Somalia*—UN peacekeepers; *Uganda*—post-conflict northern Uganda, qualitative psychological autopsy, African refugee settlement, women suicide; *Guinea*—Guinea pig, ototoxicity; *Kenya*—life questionnaire, emotional loneliness scale, family discord, faith healer; *Tanzania*—low-income country, school bullying.

#### 3.5.3. Middle East and North Africa

The Middle East and North Africa region has 20 countries shown in Table 11. All countries except a few are low-SMR countries. Three countries had much more articles associated with them than the rest: Iran—259 articles, Iraq—379 articles, and Israel—248 articles. That makes VOSviewer keyword annotations somewhat biased.

We summarized keywords in this category that repeated in more than three countries and were not highlighted by VOSviewer. The theme of euthanasia had mentions in Israel, Iran, Kuwait, and Malta. Post-traumatic stress disorder was frequent in Egypt, Iraq, Israel, and Lebanon. Conflict and disability were mentioned in association with Yemen, Syria, Libya, Algeria, and Iraq. In addition to the mentioned countries, terrorism was also associated with Morocco. Other group of keywords in Libya, Algeria, Egypt, and Morocco included maternal mortality, maternal and child health, deadly traffic injuries, and non-communicable diseases. Another big group of common shared keywords consisted of suicide terrorism, suicide bombings, childhood sexual abuse, addiction, politics of education, cannabis, pregnancy, use disorders, social media, politics, jihadism, parental death, cross-cultural comparisons, high school students, Gulf War, war, refugees, trauma, stigma, shame, political violence, emergency department, insurgency, and suicide attacks.

Some unique country-specific keywords included: *Algeria*—agency theory; authoritarian institutions; *Egypt*—addict, autoaggression; *Jordan*—seaside resort, contemporary Irish fiction; *Kuwait*—parental smoking; *Lebanon*—Syrian crisis, trans feminine; *Iran*—Khozestan Province, Iranian Kurds; *Iraq*—focused attention, mass casualty response, Pacific Rim; *Israel*—Hamas, Second Intifada, Arab, assassinations; *Oman*—antiepileptic drug; *Qatar*—Qatari women, suicide scales, menopause; *Saudi Arabia*—skin disease; Umrah worship, psychology of worship.

#### 3.5.4. East Asia and Pacific

The East Asia and Pacific region has 22 countries shown in Table 12. More countries from this group belong to the low-SMR category than others. Most articles published in relation to suicide were associated with Australia, China, Japan, South Korea, and Vietnam.

We reviewed keywords that repeated in no less than eight countries in addition to VOSviewer annotations. One keyword that was characteristic to only this region was “community psychiatry”. It appeared in keywords associated with Vanuatu, Solomon Islands, Kiribati, Tonga, Samoa, Papua New Guinea, and Micronesia. Community psychiatry is a study and treatment of individuals with complex mental illness in the community rather than in psychiatric hospitals. Interestingly, the mentioned countries had rather high SMRs. Other frequent common themes related to suicide in this region were: homicide, self-harm, hopelessness, unemployment, social support, psychological autopsy, quality of life, global burden, university students, older adults, migration, physical activity, HIV, gender differences, internet, stress, intimate partner violence, women, students, young people, loneliness, distress, smoking, alcohol, and substance use.

Mentions of country-specific unique keywords: *Japan*—Mie and Akita prefectures, medical residents, depressive state, electromagnetic field, geomagnetic storm, defeat depression campaign; *South Korea*—solar radiation, Korea Youth Risk Behavior Web-based Survey (KYRBWS), game addiction; *Australia*—Aboriginal and Torres Strait Islander, remoteness, psychiatric injury, veterinarians, chronic callers; *China*—psychological strain, negative life event, micro-blog, one child policy, celebrities, strain theory of suicide; *Fiji*—trauma registry, condoms, social learning theory, motor-vehicle-assisted suicide; *North Korea*—Cold War, Czechoslovakia, national missile defense, Korean nuclear crisis, US foreign policy, national and extended deterrence, liberal internationalism; *Singapore*—academic stress, adolescent outpatients, catatonia, train suicide, meaning-centered ethnography; *Thailand*—Thai adolescents, cardiotoxic ornamental plant species, Bangkok, suicide tree; *Indonesia*—Islamist, emotional loneliness, terrorism and social media, youth and terrorism, lone wolf terrorism, online radicalization, female jihadist, spiritual messages; *Philippines*—levodopa equivalent dose, Parkinson disease; *Cambodia*—women’s health, key somatic complaints, cultural syndromes, alcohol advertising, media exposure; *Malaysia*—suicide registry, Chrysomya megacephala, human–robot interaction, cyber aggression and victimization; *Vietnam*—post-service mortality, Vietnam theater veterans, academic pressure, low mood, prisoners of war.

#### 3.5.5. South Asia

The South Asia region has eight countries shown in Table 13. Countries in this region mostly are mix of medium- and low-SMR countries.

We summarized keywords repeated in three or more countries, some not captured by VOSviewer. In this group of countries, frequent keywords comprise education, mothers, post-natal depression, inflicted burns, farmers, policy, substance use, anxiety disorders, sleep, psychiatric disorders, religion, family, college, discrimination, culture, global health, interpersonal violence, organophosphates, post-partum depression, developing countries.

In articles associated with Pakistan, Afghanistan, and Sri Lanka, there was a group of keywords including social mechanisms, social networks, martyrdom, culture of martyrdom, axiological rationality, radicalization, jihadism, spirituality, suicide bombers, suicide terrorism, political conflict, civil war, and green revolution.

Keywords that stood in association with Pakistan, India, Afghanistan, Sri Lanka, and Bangladesh were “self-immolation” and “young married illiterate women”. These keywords originated from the article “The geographical belt of self-immolation” [37] which discusses the strictly geographically localized phenomenon of self-immolation that is specific to countries in Asia, mostly South Asia.

Country-specific unique keywords: *Sri Lanka*—yellow oleander, Kataragama, militarization, female militant, food production, suicide-like acts; *India*—Maharashtra, agrarian crisis, farmer suicides, marginal farmers, Kerala, Jainism; *Nepal*—Edinburgh Postnatal Depression Scale, thinking healthy program, mother–child interaction; *Pakistan*—chemical burn, caliphate, sexual infidelity, divorcee individuals, Sunni–Shia sectarianism; *Afghanistan*—survey sampling, chronic pain, US army, behavioral activation, symptom clusters, induced traumatic stress; *Bangladesh*—tetanus, micro-credit, women with disabilities, female garment workers, psychosocial well-being; *Bhutan*—parental engagement.

#### 3.5.6. Latin America and Caribbean

The Latin America and Caribbean region has 31 countries shown in Table 14. In this region, countries are low- and medium-SMR countries except for Guyana, Suriname, and Uruguay. The SMR in these countries was quite high. Countries in this region did not have many associated publications except Brazil and Mexico. For this region, in addition to VOSviewer annotations, we reviewed keywords that repeated in four or more countries. In high-SMR countries of this region, the most important themes were suicide in young people, middle and high school students, teenagers, depression, and alcohol drinking. Other common themes repeating across countries were mental heath, public health, stressful life events, sexual abuse, smoking, drugs, trauma, spirituality, accidents, risk behaviors, self-esteem, thyroid abnormalities, spectrum disorders, victimization, gender differences, guns, and school.

Euthanasia had a few mentions in Chile, Mexico, Uruguay, and Colombia. Obesity and its relationship to depression, mental health, and suicide was studied in Brazil, Mexico, Chile, and Costa Rica. The topic of obesity in the context of suicide also appears in other regions except Sub-Saharan Africa.

Country-specific unique keywords in this region: *Guyana*—toxic plants, catharsis, mass suicide; *Suriname*—global health governance, multi-lateral development banks, mercury exposure; *Uruguay*—cocaine base post, historical evolution; *Chile*—dangerous behavior, cocaine-related disorders, hidden populations; *Cuba*—epidemiology descriptive, refractory temporal lobe epilepsy, deaths from epilepsy; *Argentina*—Maria Ines Krimer, thyroid-stimulating hormone, iodine, mental health policies, sex workers, aggressors, cyberbullying perpetration; *Guatemala*—school health survey, sexual initiation; *Haiti*—community health workers, rural Haiti, adolescent depression; *Jamaica*—sickle cell disease, underachievement, packer syndrome, internalizing distress; *Peru*—subjective constitution, fatherhood, depressive symptoms, antepartum depressive symptoms; *Brazil*—the elderly, psychosocial autopsy, suicide among elderly; *Colombia*—war crimes, sexual dissidence, persons deprived of freedom, unconstitutional state of things, rights of the prisoners, pesticide toxicity; *Mexico*—international consortium, school population survey, amyloid plaques, addiction, attitude towards institutional authority, child suicide; *Nicaragua*—family of origin, hospital surveillance, seasonal pattern; *Paraguay*—scopadulcic acid b, traditional herbal medicines of guanary indio, bone resorption; *Venezuela*—antibiotics, Aeromonas spp.

#### 3.5.7. North America

The North America region includes the US (3563 articles) and Canada (730 articles) shown in Table 15. Both represent medium-SMR countries. Together, the US and Canada surpass all other countries by the number of articles on suicide. Top-ranking topics by the frequency of mentions in articles associated with both countries comprise depression, euthanasia, mental and psychiatric disorders, adolescents, children, gender, Inuit, substance use, firearms, and violence in general.

It is difficult to comprehensively review very numerous unique keywords associated with North American countries. We mention only a few of the most frequent. For a full list, see the data availability statement. Unique country-specific keywords associated with North American countries: *US*—non-Hispanic whites, reporting system, mass shootings, youth violence, DSM-IV disorders (mental retardation and intellectual disability disorders), multiple births, households, rampage shootings, decedents; *Canada*—Nunavut, Metis, home care, CAG repeats, Alberta, marijuana use evidence, British Columbia, narrative research, body donation, aboriginal young people, Edmonton, correctional service of Canada, interpersonal trauma, hospital pharmacists, Dutch program.

### 3.6. Word Embedding of Suicide-Related Keywords

Having such a large corpus of expressions related to suicide, an exploration of contexts and semantic neighborhoods of keywords of interest is not possible through analysis of just keyword co-occurrences. We attempted to carry out word embedding for all suicide-related keywords that we collected from 19,000 articles. To do that, we trained a recurrent neural network to discriminate countries based on the keywords in the suicide-related articles for a particular country. The main aim of the network training was to derive word embedding vectors rather than classification of countries by keywords.

**Figure 11:**
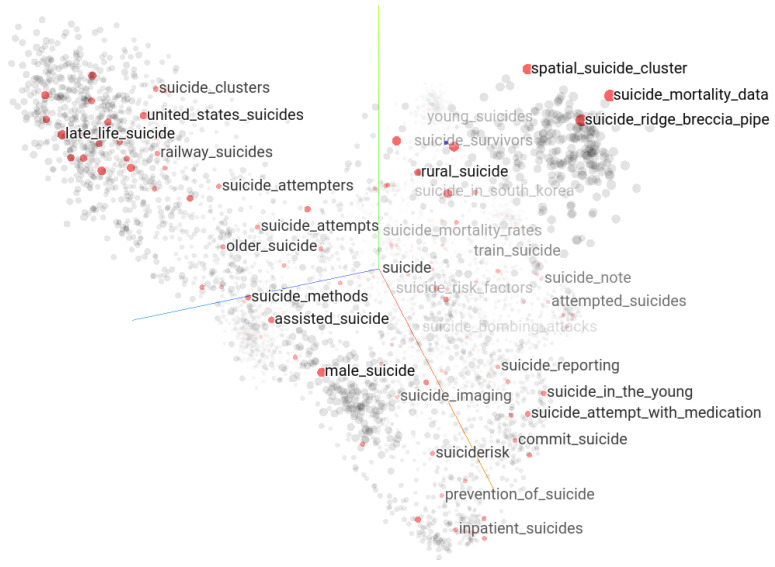
Suicide vocabulary mapped in Keras embedding projector tool in 3D UMAP projection in which the string “suicide” is highlighted (685 matches).

Semantic proximity between keywords in the suicide context can be explored as a neighborhood of the word embedding vectors in their high-dimensional Euclidean space. The keywords whose embedding vectors are close in their original high-dimensional space must be close semantically in that context from which the embedding vectors were derived. The lower-dimensional representation of the word embedding vectors corresponding to the 23,826 suicide-related keywords and expressions in 3D UMAP projection using the Keras embedding projector tool [35] shows a discernible structure presented in Figure 11. The expression “suic” highlights 687 keyword matches of which any matching keyword can be selected to explore its neighborhoods further.

**Figure 12:**
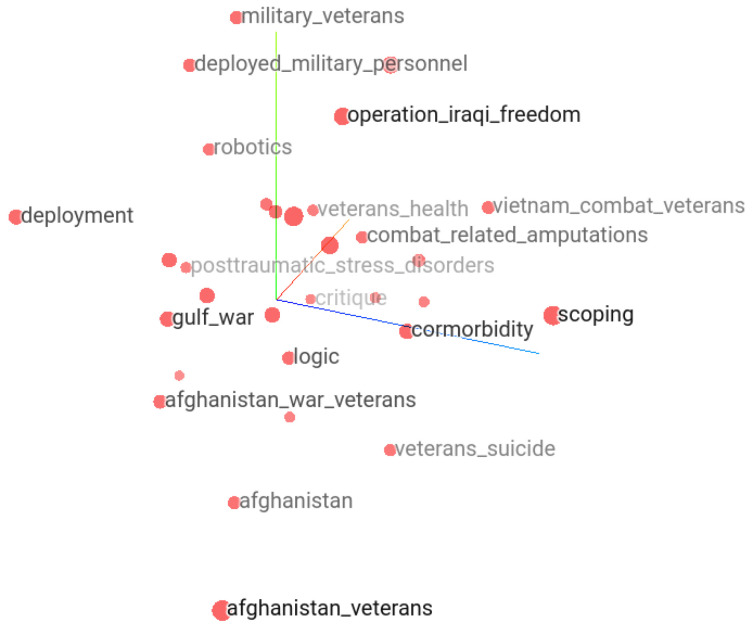
Closest neighbors of “Afghanistan” keyword are “Afghanistan war veterans”, “Afghanistan veterans”, “veterans’ suicide”.

In the following, we attempt to demonstrate how we can ask questions and explore neighborhoods of topics semantically related to the keywords of interest by the help of keyword embedding vectors. Besides the Keras embedding projector, selected groups of keyword embedding vectors can also be explored for proximity by other mappings such as principal component analysis or multi-dimensional scaling. Here, using word embedding vectors, we attempt to demonstrate how a single keyword surrounded by other semantically close keywords helps reasoning. For example, Figure 12 shows the 30 closest keywords to “Afghanistan” in the TensorFlow embedding projector. Each keyword is at a certain distance from “Afghanistan”. In the context of suicide, the “Afghanistan” keyword is surrounded by terms indicating war veterans, veteran suicide, post-traumatic stress disorder, veteran’s health, and combat-related amputations that reflect topics in papers related to Afghanistan and suicide.

The most discussed topics on suicide and Afghanistan include war veterans and traumatic health events. Themes from the corpus of keywords and expressions in the context of suicide can be studied using word embedding. It reveals possibly semantically close keywords in the selected topic. For example, similar analysis of “children left behind” showed Balkans, intimate partner homicide suicide, obsessive compulsive symptoms, Groningen Protocol, Perestroika, migrant parents, and weight control behaviors as the closest keywords. The keywords “migrant parents” and/or “intimate partner homicide suicide” semantically relate to “children left behind”.

### 3.7. How Keyword Analysis and Word Embedding Helps: Use Case about Loneliness

Loneliness is one of the suicide risk factors strongly linked to depression. Using collected keywords, we computed co-occurrences of the term “loneliness” with other keyword phrases in each region. Phrases frequently coming together were aggregated across regions. Some repeating phrases were seen in multiple regions. Nevertheless, there were terms specific to a particular region. Here, we summarize some of the observed regularities.

The most frequent expressions coming together were “suicide” and “loneliness”. This combination was frequently seen in all regions except Sub-Saharan Africa (SSA). The combination of “loneliness” and “depression” was also found. The combination of “prevalence” and “loneliness” was frequently seen in all regions except for the Middle East and North Africa (MENA). In the MENA region, appearances of “loneliness” were quite rare compared to other regions.

We may hypothesize that, in in the SSA region, loneliness does not have a strong connection to suicide. The most frequent SSA-region-specific phrases paired with loneliness were child, collective self-esteem, attachment theory, homeless youth, Kampala lifestyle, abuse, and alcohol consumption. Especially frequent in this context were parental neglect, sadness, school-going adolescents, street youth, trading sex, and vulnerable youth. In the North America (NA) region, the frequent contexts in which loneliness was found were minority stress, Aboriginals, social loneliness, parasuicide, perceived burden, poor health, sexual orientation, sleep disturbances, social engagement, thwarted belonging, veterans, ethnicity, eventual suicide, anxiety sensitivity, bariatric surgery, and weight. The contexts of loneliness specific to Europe and Central Asia differed from those previously mentioned and they were mental disorders, older people, poverty, shyness, social factors, social exclusion, social recognition, cyberbullying, cyber victimization, death wishes, impulsivity, and interpersonal relations. The East Asia and Pacific region had the largest number of phrases paired with loneliness. Since there were many, we mention only a few here: migration, Muslim, physical activity, prolonged social withdrawal, public holidays, religion, resilience, self-esteem, substance use, unemployment, and urbanization.

From the observed relationships between phrases frequently occurring and co-occurring in the literature, we gained a practical insight into different topics surrounding loneliness in the context of suicide in different regions. In Sub-Saharan Africa, it points to child abuse, in North America it highlights obesity problems, in Europe it points to social problems and cyberspace, and in East Asia and the Pacific it points to rural life. The code to extract co-occurrences is available on GitHub [28].

Word embedding space exploration of “loneliness” as the closest neighbor gave the semantically close phrase “children left behind”. This phrase was not in the matrix of co-occurrences and word embedding in this case brought into focus a semantically related topic.

## 4. Discussion

Recent work [38] analyzed the disparity in publishing on suicide across countries by using the Scopus bibliographic database. It revealed the fact that about 78% of articles were published from two regions—Europe and the Americas. From low- and middle-income countries, only China and India were highly published countries. Our collected bibliographical information results from the Web of Science database were in agreement with those findings. We also identified that Australia had many publications on suicide research.

Retrospective analysis of suicide that focused on the causes and determinants of suicide [39,40,41] showed that political factors such as laws restricting access to alcohol and firearms and social welfare expenditures and social factors such as marriage, parenting, and religiousness were found as preventive against suicide, while media coverage of celebrity suicide could have negatively affected vulnerable persons. Economic factors such as unemployment, debt, low income, and economic recessions were identified as strong predictive factors of suicide behavior. Keyword analysis allowed us to notice that economic recession as related to suicide has gained more focus in Greece.

Socioeconomic factors, unemployment, unhealthy lifestyle, and mental disorders were highlighted [16,17,18] as factors of high risk of suicide. Our study does not provide tools for causal inference. Nevertheless, its data can aid in understanding the wider scope and regional specificity of the risk factors. We demonstrated that regionally specific phrases occurring together with “loneliness” are different for each region. From this, we could hypothesize about factors strongly linked to loneliness in the suicide context in different regions: child neglect emerged in the Sub-Saharan Africa region, obesity in North America (complements unhealthy lifestyle), and social exclusion in Europe. The overall results of our study in a systematic way address suicide research topics and directions that gained the most attention in the literature from 2000–2019 across groups of high-, medium-, and low-SMR countries and geographic regions. For example, HIV/AIDS in the African continent, war-related subjects in the Middle East region, farmer suicides in India, and euthanasia in European countries and North America.

Depression is a central theme in the literature associated with suicide across all countries and all regions, as well as other prevailing topics related to depression such as major depressive disorder, suicide in youth, euthanasia, alcohol usage, hopelessness, loneliness, and unemployment. It is no surprise that genetic, genomic, molecular, and neurochemical underpinnings [42,43,44] and neurobiology of suicide [45] are rapidly evolving areas overlapping significantly with research into molecular mechanisms of depression [46]. Even in this area, keyword embedding may help to discover close semantic neighbors. For example, the *SLC6A4* gene (associated with depression) is in close proximity to “Vietnam veterans”, “mental health policies”, and “mushroom”. Specifically, the *SLC6A4* gene was investigated in Vietnam veterans suffering from depression and some studies showed that psilocybin present in some mushroom species has antidepressant potential. Indeed, not all proximal terms are meaningful, but some are informative. A search using word embedding has very practical utility. It helps to overcome information explosion if one is looking to make a meaningful inference from tens of thousands of interrelated keywords.

Highlighted country-specific keywords and maps of interconnected keywords may be useful as exploratory tools in the development of prevention strategies. They can help to notice important themes and to understand a target population to adopt better practices. For example, in South Asia there is a phenomenon of self-immolation in young married women that occurs in a strict geographic area [37]. In Iceland, friendship networks have been shown to support peer delinquency [47]. The preventive strategies in both examples may consider factors pertaining to these phenomena.

Text mining in suicide research has been applied to mine articles, social media, and electronic health records and helped to identify risk factors and track trends [48]. In suicide research, document classification and natural language processing are the most widely used methods [49]. Our work differs from conventional text mining applications in that it uses a semi-supervised analysis of keywords in articles in an attempt to reveal differences and similarities between countries and regions with regard to suicide research rather than to design an algorithm to predict the risk of a suicide. As such, results of our work may enrich, complement, and facilitate text mining algorithms aimed at suicide risk prediction from textual data.

Our study has certain limitations. First, there could be errors in statistical data of suicide mortality rates. These data are usually collected from death certificates and in some countries there might be omissions and reported wrong reasons. Second, it is very difficult to establish hard criteria for N in the analysis of the top N keywords and keyword rankings. Usually, the very top keywords are not informative and there is a tradeoff between imposing a limit on the number of top keywords and selecting informative keywords when using VOSviewer and other approaches. Third, there was no reference to the assignment of country into high-, medium-, and low-SMR group. The assignment is data-driven, determined by the limits of summary statistics and tied to the period 2000–2019. If we wanted to include more years and SMR went higher than the existing maximum, or there was a massive drop in SMR in many countries, then the thresholds for high, medium and low would shift.

## 5. Conclusions

In this exploratory study of the age-standardized suicide mortality rate (SMR) dynamics from 2000–2019 worldwide using machine learning, we grouped countries into high-, medium-, and low-SMR groups and performed detailed analysis of keywords tagging articles on suicide in each country.

Our study made two novel contributions. First, we summarized total SMRs in 183 countries from 2000–2019 statistically and, using data-driven percentile intervals of age-standardized SMR statistical distribution, we grouped countries into high-, medium-, and low-SMR groups across geographic regions. We numerically highlighted some regional differences. MENA countries were found in a low-SMR category compared to others. Former USSR countries and some African countries were found in a high-SMR category. We demonstrated that countries formed naturally different groups in terms of their SMR magnitude and dynamics from 2000–2019. Second, aiming to understand and explain SMR disparities between groups of countries and geographic regions, we collected 19,000 bibliographic records and the analyzed authors and extended keywords of articles associated with suicide and a particular country. The analysis was focused on frequently occurring keywords and their combinations in high-, medium-, and low-SMR country groups and across geographic regions. During this process, we collected a corpus of phrases related to suicide and made word embedding vector representations of these keywords that allowed us to find semantic relationships between keywords to enrich other simpler analyses. To our knowledge, this is a first attempt to create a corpus and subsequently a map of topic relationships for suicide research.

Through keyword analysis, we were able to at least partially address disparities between high-, medium-, and low-suicide-mortality-rate country groups and geographic regions through keyword analysis. In addition, we provided a breakdown of repeating and shared keywords in each group of countries in separate geographic regions. It aimed at understanding what might be common or specific between low-, medium-, and high-suicide-mortality-rate countries across different geographic regions. We used an algorithm to find word embedding vectors and metadata using a corpus of keywords and phrases extracted from suicide-related papers published with reference to a specific country. This allowed us to make logical inferences and analyze the semantic scope in which these keywords were used in the context of suicide.

Amount of research works addressing suicide is vast. We attempted to navigate through it in a systematic way utilizing analysis of keywords. It revealed variety of most frequent themes associated with suicide raising awareness of existing problems that may positively impact prevention and literacy campaigns. 

The methodology used in our study can be adopted to perform other similar analysis of different indicator datasets and situations where quantitative analysis revealed objective regularities that are difficult to explain. We advocate using keyword analysis, machine learning, and neural-net-enabled bibliographic text mining to study poorly characterized phenomena.

## Figures and Tables

**Table 1:** Percentile intervals of age-standardized suicide mortality rate corresponding to SMR categories spanning from low to high.

Interval of Percentiles	Label of the SMR Level	Abbreviation	Corresponding Interval of Age-Standardized Suicide Mortality Rate per 100,000 Population
[0, 10)	Low	L	[0, 3.4)
[10, 20)	Low medium	LM	[3.4, 5.1)
[20, 30)	Low high	LH	[5.1, 6.6)
[30, 40)	Medium low	ML	[6.6, 8.119)
[40, 50) ^1^	Medium	M	[8.119, 9.9)
[50, 60)	Medium	M	[9.9, 11.5)
[60, 70)	Medium high	MH	[11.5, 13.7)
[70, 80)	High low	HL	[13.7, 16.6)
[80, 90)	High medium	HM	[16.6, 22.8)
[90, 100]	High	H	[22.8, 117]

^1^ 50th percentile represents median value. Interval around median value selected as medium SMR.

**Table 2:** Example of quantification and labeling of Estonia’s SMR category.

Year	2000	2001	2002	2003	2004	2005	2006	2007	2008	2009	2010	2011	2012	2013	2014	2015	2016	2017	2018	2019
SMR Estonia	25	26.1	25.8	22.3	23.1	19.1	16.8	17.5	16.2	19.2	15.4	14.7	17.2	14.9	16.2	13.4	12.5	15.2	11.6	12
Percentile interval	[90, 100]	[90, 100]	[90, 100]	[80, 90)	[90, 100]	[80, 90)	[80, 90)	[80, 90)	[70, 80)	[80, 90)	[70, 80)	[70, 80)	[80, 90)	[70, 80)	[70, 80)	[60, 70)	[60, 70)	[70, 80)	[60, 70)	[60, 70)
Bin	[22.8, 117)	[22.8, 117)	[22.8, 117)	[16.6, 22.8)	[22.8, 117)	[16.6, 22.8)	[16.6, 22.8)	[16.6, 22.8)	[13.7, 16.6)	[16.6, 22.8)	[13.7, 16.6)	[13.7, 16.6)	[16.6, 22.8)	[13.7, 16.6)	[13.7, 16.6)	[11.5, 13.7)	[11.5, 13.7)	[13.7, 16.6)	[11.5, 13.7)	[11.5, 13.7)
Label	H	H	H	HM	H	HM	HM	HM	HL	HM	HL	HL	HM	HL	HL	MH	MH	HL	MH	MH

**Table 3:** Example of quantification and labeling of Qatar’s SMR category.

Year	2000	2001	2002	2003	2004	2005	2006	2007	2008	2009	2010	2011	2012	2013	2014	2015	2016	2017	2018	2019
SMR Qatar	7.6	7.6	7.4	6.9	6.6	6.3	6.1	7.2	6.8	6.7	6.4	6.2	5.8	5.3	5	4.9	4.8	4.6	4.6	4.7
Label	ML	ML	ML	ML	ML	LH	LH	ML	ML	ML	LH	LH	LH	LH	LM	LM	LM	LM	LM	LM

**Table 4:** Summary statistics of age-standardized SMR per 100,000 population in 183 countries from 2000–2019.

Statistics	Males	Females	Both Genders
Minimum	0	0	0
1st Quartile (Q25)	8.3	2.5	5.6
Median (Q50)	14.5	4.75	9.8
3rd Quartile (Q75)	23.9	7.2	14.9
Maximum	195.2	46.3	116.2
Mean	18.75	5.4	9.8
Standard Deviation	16.99	4.2	11.7

**Table 5:** High-SMR country clusters stratified by geographic regions, C4, C5, C6, C7, C9, C10.

Cluster	Geographic Region	Country
C4, Very high SMR N = 1	Sub-Saharan Africa n = 1	Eswatini
C5, Very high SMR N = 1	Sub-Saharan Africa n = 1	Lesotho
C6, High SMR N = 2	East Asia and Pacific n = 1	Kiribati
	Latin America and Caribbean n = 1	Guyana
C7, High SMR N = 5	Europe and Central Asia n = 4	Belarus, Kazakhstan, Lithuania, Russian Federation
	Sub-Saharan Africa n = 1	Botswana
C9, High SMR N = 12	East Asia and Pacific n = 2	Mongolia, Vanuatu
	Europe and Central Asia n = 5	Estonia, Hungary, Latvia, Slovenia, Ukraine
	South Asia n = 1	Sri Lanka
	Sub-Saharan Africa n = 4	Cote d’Ivoire, Eritrea, Namibia, Zambia
C10, High SMR N = 7	East Asia and Pacific n = 2	Micronesia, South Korea
	Latin America and Caribbean n = 2	Suriname
	Sub-Saharan Africa n = 4	Central African Republic, Mozambique, South Africa, Zimbabwe

**Table 6:** Mixed medium- and high-SMR country cluster stratified by geographic region, C8 (N = 36).

Geographic Region	Country
East Asia and Pacific n = 3	Japan, Samoa, Solomon Islands
Europe and Central Asia n = 11	Austria, Belgium, Croatia, Finland, France, Kyrgyzstan, Moldova, Montenegro, Poland, Serbia, Switzerland
Latin America and Caribbean n = 1	Uruguay
South Asia n = 1	India
Sub-Saharan Africa n = 20	Angola, Benin, Burkina Faso, Burundi, Cabo Verde, Cameroon, Chad, Congo, Dem. Rep. Congo, Equatorial Guinea, Ethiopia, Gabon, Guinea-Bissau, Kenya, Malawi, Rwanda, Senegal, Somalia, Togo, Uganda

**Table 7:** Medium-SMR country clusters stratified by geographic regions in C2 and C3, total N_countries_ = 63 countries.

Cluster	Geographic Region	Country
Cluster C2 Medium SMR, N = 34	East Asia and Pacific n = 5	Laos, North Korea, Singapore, Thailand, Vietnam
	Europe and Central Asia n = 11	Bosnia and Herzegovina, Bulgaria, Denmark, Georgia, Germany, Macedonia, Netherlands, Portugal, Romania, United Kingdom, Uzbekistan
	Latin America and Caribbean n = 7	Argentina, Belize, Bolivia, Costa Rica, Ecuador, El Salvador, St. Lucia
	Middle East and North Africa n = 3	Bahrain, Morocco, Yemen
	South Asia n = 1	Afghanistan
	Sub-Saharan Africa n = 7	Comoros, Liberia, Mali, Mauritius, Nigeria, South Sudan, Seychelles
Cluster C3 Medium High SMR N = 29	East Asia and Pacific n = 4	Australia, China, Fiji, New Zealand
	Europe and Central Asia n = 8	Czechia, Iceland, Ireland, Luxembourg, Norway, Slovakia, Sweden, Turkmenistan
	Latin America and Caribbean n = 5	Chile, Cuba, Guatemala, Haiti, Trinidad and Tobago
	Middle East and North Africa n = 1	Djibouti
	North America n = 2	United States, Canada
	South Asia n = 2	Nepal, Pakistan
	Sub-Saharan Africa n = 7	Gambia, Ghana, Guinea, Madagascar, Niger, Sierra Leone, Tanzania

**Table 8:** Low-SMR country clusters stratified by geographic regions, C1, N = 56 countries.

Geographic Region	Country
East Asia and Pacific n = 9	Brunei Darussalam, Cambodia, Indonesia, Malaysia, Myanmar, Papua New Guinea, Philippines, Timor Leste, Tonga
Europe and Central Asia n = 9	Albania, Armenia, Azerbaijan, Cyprus, Greece, Italy, Spain, Tajikistan, Turkey
Latin America and Caribbean n = 16	Antigua and Barbuda, Bahamas, Barbados, Brazil, Colombia, Dominican Republic, Grenada, Honduras, Jamaica, Mexico, Nicaragua, Panama, Paraguay, Peru, St. Vincent and the Grenadines, Venezuela
Middle East and North Africa n = 16	Algeria, Egypt, Iran, Iraq, Israel, Jordan, Kuwait, Lebanon, Libya, Malta, Oman, Qatar, Saudi Arabia, Syria, Tunisia, United Arab Emirates
South Asia n = 3	Bangladesh, Bhutan, Maldives
Sub-Saharan Africa n = 3	Mauritania, Sao Tome and Principe, Sudan

**Table 9:** SMR in Europe and Central Asia geographic region.

Cluster, SMR Category	Country	[SMR Q25, SMR Median, SMR Q75]	SMR Label	WoS Records	VOSviewer Keyword Annotations
C1, Low SMR N = 56 n = 9	Albania	[4.425, 4.85, 7.625]	^1^ Low Med.	12	Abuse, adolescents, schizophrenia, alcohol, depression, hopelessness, children, unemployment, financial crisis, economic crisis, mental health, care, euthanasia.
	Armenia	[3.1, 4.15, 5.075]	Low	2	
	Azerbaijan	[3.9, 4.15, 4.525]	Low	4	
	Cyprus	[2.25, 3.45, 4.3]	Low	9	
	Greece	[2.675, 2.9, 3.7]	Low	146	
	Italy	[4.775, 4.9, 5.3]	Low	296	
	Spain	[5.475,5.7,6.2]	Low	298	
	Tajikistan	[4.775, 5, 5.125]	Low	2	
	Turkey	[2.3, 2.5,3.05]	Low	324	
C2, Medium SMR N = 34 n = 11	Bosnia and Herzegovina	[8.2, 8.35, 8.5]	Medium	6	Adolescents, children, depression, schizophrenia, self-harm, assisted suicide, cancer, care, euthanasia, end-of-life decisions, palliative care, physicians.
	Bulgaria	[7.025, 8.55, 9.825]	Medium Wi.	13	
	Denmark	[8.75, 9.75, 11.1]	Medium	340	
	Georgia	[6.325, 7.25, 7.95]	Medium	32	
	Germany	[8.975, 9.2, 9.95]	Medium	462	
	Macedonia	[7.275, 8, 8.35]	Medium Wi.	0	
	Netherlands	[8.075, 8.35, 9.3]	Medium	639	
	Portugal	[7.5, 7.95, 8.625]	Medium	66	
	Romania	[9.075, 9.95, 10.725]	Medium	66	
	United Kingdom	[7, 7.25, 7.525]	Medium	253	
	Uzbekistan	[8.85, 9.1, 9.9]	Medium	2	
C3, Medium high SMR N = 29 n = 8	Czechia	[11.2, 11.85, 12.95]	Medium Hi.	18	Adolescents, bipolar disorder, children, depression, major depression, mental disorders, schizophrenia, self-harm, alcohol, antidepressants, gender, homicide, prevention, abuse, women, parasuicide.
	Iceland	[11.45, 12.15, 12.75]	Medium Hi.	32	
	Ireland	[10, 11.05, 11.55]	Medium	1258	
	Luxembourg	[8.875, 9.7, 11.6]	Medium	18	
	Norway	[10.37, 10.95, 11.45]	Medium	238	
	Slovakia	[9.875, 10.5, 11.975]	Medium	5	
	Sweden	[12.075, 12.35, 12.7]	Medium Hi.	558	
	Turkmenistan	[6.975, 9.15, 14.825]	Medium Wi.	0	
C7, High SMR N = 5 n = 4	Belarus	[21.1, 30.3, 36.35]	High	36	Adolescents, children, depression, mental health, alcohol consumption, homicide, former USSR, inequalities, men, behavior.
	Kazakhstan	[24, 29.5, 38.375]	High	9	
	Lithuania	[29.125, 31.55, 37.2]	High	67	
	Russian Federation	[28.8, 33.15, 43.675]	High	28	
C8, Mixed medium and high SMR N = 36 n = 11	Austria	[11.475, 12.1, 13.375]	Medium Hi.	158	Children, depression, Finland, gender, mental disorders, schizophrenia, assisted suicide, Belgium, care, euthanasia, Netherlands, end-of-life decisions, palliative care, Switzerland, Austria, homicide, prevention.
	Belgium	[15.075, 16.55, 16.7]	High	264	
	Croatia	[12.1, 13.2, 14.925]	Medium Hi.	52	
	Finland	[14.4, 16.65, 18.375]	High	380	
	France	[11.475, 13.85, 14.925]	High Lo.	415	
	Kyrgyzstan	[11.275, 12.6, 14.425]	Medium Hi.	1	
	Moldova	[13.625, 15.8, 17.1]	High	3	
	Montenegro	[16.3, 17.15, 17.925]	High	6	
	Poland	[13.275, 14.95, 15.225]	High Lo.	90	
	Serbia	[11.925, 13.65, 15.875]	High Lo.	35	
	Switzerland	[10.475, 11.4, 13.925]	Medium Hi.	284	
C9, High SMR N = 12 n = 5	Estonia	[14.85, 16.5, 19.975]	High	48	Adolescence, deliberate self-harm, gender differences, life events, mental disorders, parasuicide, schizophrenia, seasonality, age, alcohol, Europe, childhood, depression, general population, help seeking, symptoms, women.
	Hungary	[14.9, 19, 20.775]	High	128	
	Latvia	[17.1, 18.85, 22.1]	High	10	
	Slovenia	[14.7, 16.05, 21.325]	High	70	
	Ukraine	[18.525, 21.1, 28.325]	High	24	

^1^ Meaning of composite labels with respect to falling into intervals defined by Table 1: Low Hi.—SMR is concentrated mostly in low high interval; Low Med.—SMR is frequently on the borderline of low and medium intervals; Medium Lo.—SMR concentrated in medium low interval; Medium Wi.—SMR is wide spectrum, it is very variable and spans many intervals; Medium Hi.—SMR is frequent in medium high and high low intervals; High Lo.—SMR is concentrated in high low interval.

**Table 10:** SMR in Sub-Saharan Africa geographic region.

Cluster, SMR Category	Country	[SMR Q25, SMR Median, SMR Q75]	SMR Label	WoS Records	VOSviewer Keyword Annotations
C1, Low SMR N = 56 n = 2	Mauritania	[5.4, 5.55, 5.825]	Low Hi.	1	Adolescent suicide, ingestion, deadly traffic injuries, maternal and child health, maternal mortality, non-communicable diseases, sustainable development, epidemic, pandemic strains, transcriptional activator.
	Sao Tome and Principe	[2.2, 2.2, 2.225]	Low	0	
	Sudan	[4.975, 5.2, 5.325]	Low Hi.	5	
C2, Medium SMR N = 34 n = 7	Comoros	[8.6, 8.9, 9.15]	Medium	0	Depression, disorders, population, schizophrenia, Boko Haram, gender, health, terrorism, women, HIV/AIDS, stigma, suicidality, adolescents, homicide, Nigeria, psychiatric disorders.
	Liberia	[8.15, 8.5, 8.625]	Medium	2	
	Mali	[8.575, 8.7, 8.725]	Medium	4	
	Mauritius	[7.75, 8.65, 9.65]	Medium	0	
	Nigeria	[7.525, 8.9, 9.2]	Medium	52	
	South Sudan	[6.575, 6.7, 7.125]	Medium Lo.	0	
	Seychelles	[7.5, 7.7, 8.5]	Low Hi.	1	
C3, Medium high SMR N = 29 n = 7	Gambia	[10.525, 10.95, 11.32]	Medium	1	Contemporary Ghana, nurses, prevention, self-harm, students, Uganda, children, community, mental health, adolescents, depression, gender differences, homicide, women, psychology students, stigma, South Africa, Tanzania.
	Ghana	[11.225, 12.1, 12.825]	Medium	53	
	Guinea	[10.4, 12.05, 12.8]	Medium Hi.	30	
	Madagascar	[9.725, 10.2, 10.625]	Medium	3	
	Niger	[10.075, 10.15, 10.4]	Medium	3	
	Sierra Leone	[10.875, 11.1, 11.7]	Medium	1	
	Tanzania	[8.3, 10.05, 10.925]	Medium	14	
C4, Very high SMR N = 1	Eswatini	[45.92, 56.65, 66.075]	High	0	NA
C5, Very high SMR N = 1	Lesotho	[47, 85.75, 97.725]	High	0	NA
C7, High SMR N = 5	Botswana	[25.1, 31.65, 40.9]	High	0	NA
C8, Mixed medium and high SMR N = 36 n = 20	Angola	[13.25, 14.2, 17.125]	High	0	Abuse, adolescents, care, community, gender, HIV/AIDS, Uganda, women, children, mental health, post-traumatic stress, trauma, war, deliberate self-harm, psychological autopsy, schizophrenia, Sub-Saharan Africa, anxiety, depression, health, Kenya, primary care, stress, validation.
	Benin	[13.55, 14.1, 14.7]	High Lo.	7	
	Burkina Faso	[15.55, 15.8, 15.9]	High	2	
	Burundi	[13.025, 15.05, 18.05]	High	0	
	Cabo Verde	[16.85, 18.1, 18.225]	High	0	
	Cameroon	[17.7, 19.3, 19.625]	High	9	
	Chad	[14.45, 15.65, 15.8]	High Lo.	2	
	Congo	[13.25, 15.9, 17.625]	High	10	
	Dem. Rep. Congo	[12.55, 13.4, 14.125]	Medium Hi.	1	
	Equatorial Guinea	[14.05, 14.5, 15.35]	High	0	
	Ethiopia	[10. 13.4, 15.425]	Medium Hi.	52	
	Gabon	[14.45, 15.75, 16.375]	High	0	
	Guinea-Bissau	[13.85, 15.45, 15.75]	High Lo.	1	
	Kenya	[11.075, 12, 12.925]	Medium	23	
	Malawi	[12.75, 14.7, 15.75]	High Lo.	12	
	Rwanda	[10.05, 11.8, 15.3]	Medium Hi.	6	
	Senegal	[11.95, 12.4, 13.325]	Medium	1	
	Somalia	[15.15, 15.65, 16.525]	High	7	
	Togo	[17.15, 18.05, 18.625]	High	3	
	Uganda	[11.32, 15.05, 20.425]	High Lo.	70	
C9, High SMR N = 12 n = 4	Cote d’Ivoire	[18.3, 19.65, 20.675]	High	0	Adolescents, anxiety, care, criminalization, gay, heteronormativity, HIV, sexual and gender minorities, stigma, abuse, alcohol use, asylum applicants.
	Eritrea	[18.85, 20.6, 20.975]	High	1	
	Namibia	[15.125, 18.2, 24.55]	High	5	
	Zambia	[17.6, 19.35, 21.875]	High	0	
C10, High SMR N = 7 n = 4	Central African Rep.	[26.525, 27.6, 28.2]	High	1	Alcohol use, homicide, women, care, depression, HIV/AIDS, stress, adolescents, gender, men, students, violence, youth, post-traumatic stress, suicide prevention.
	Mozambique	[21.325, 23.15, 24.625]	High	9	
	South Africa	[25.25, 26.55, 27.6]	High	232	
	Zimbabwe	[21.87, 26.55, 31.475]	High	21	

**Table 11:** SMR in Middle East and North Africa geographic region.

Cluster, SMR Category	Country	[SMR Q25, SMR Median, SMR Q75]	SMR Label	WoS Records	VOSviewer Keyword Annotations
C1, Low SMR N = 56 n = 16	Algeria	[2.775, 3.1, 3.85]	Low	11	Adolescents, children, injuries, Iran, Israel, population, self-immolation, terrorism, women, Afghanistan, care, combat, deployment, mental health problems, military, military personnel, post-traumatic stress disorder, resilience, trauma, veterans, war, depression, mental health, social support.
	Egypt	[3.575, 3.6, 3.7]	Low	24	
	Iran	[5.775, 6, 6.275]	Low	259	
	Iraq	[5.1, 5.2, 5.325]	Low	397	
	Israel	[5.1, 5.5, 6.15]	Low	248	
	Jordan	[1.9, 2.05, 2.85]	Low	11	
	Kuwait	[2.4, 2.65, 2.7]	Low	12	
	Lebanon	[2.875, 2.9, 3]	Low	35	
	Libya	[4.95, 5.35, 5.5]	Low	6	
	Malta	[4.9, 5.45, 6.025]	Low	3	
	Oman	[4.7, 6.05, 6.325]	Low	4	
	Qatar	[4.975, 6.25, 6.825]	Medium Lo.	4	
	Saudi Arabia	[4.075, 5.3, 5.925]	Low	31	
	Syria	[1.8, 1.85, 2]	Low	18	
	Tunisia	[3.175, 3.4, 3.725]	Low	0	
	United Arab Emir.	[5.525, 6.5, 6.8]	Medium Lo.	7	
C2, Medium SMR N = 34 n = 3	Bahrain	[6.65, 7.2, 8.75]	Medium	3	Terrorism, comorbidity, gender, schizophrenia, community, depression, liaison psychiatry, children, Morocco, poisoning, risk factors, violence.
	Morocco	[7.825, 9.6, 10.125]	Medium	24	
	Yemen	[7.1, 7.5, 8.025]	Medium	6	
C3, Medium high SMR N = 29 n = 1	Djibouti	[11.07, 11.95, 12.1]	Medium Hi.	0	NA

**Table 12:** SMR in East Asia and Pacific geographic region.

Cluster, SMR Category	Country	[SMR Q25, SMR Median, SMR Q75]	SMR Label	WoS Records	Keyword Annotations
C1, Low SMR N = 56 n = 9	Brunei Darussalam	[1.6, 1.8, 2.25]	Low	1	Adolescents, children, depressive symptoms, mental health, psychological distress, religion, youth, drug, gender, hopelessness, behavior, college students, self-harm, social support, depression, prevention, reliability, students, terrorism, validity.
	Cambodia	[5.8, 6.35, 6.725]	Low Med.	19	
	Indonesia	[2.6, 2.95, 3.35]	Low	32	
	Malaysia	[5.275, 5.6, 5.8]	Low	77	
	Myanmar	[3.375, 3.85, 4.525]	Low	7	
	Papua New Guinea	[3.2, 3.3, 3.5]	Low	6	
	Philippines	[2.3, 2.55, 2.725]	Low	28	
	Timor Leste	[4.2, 4.4, 4.5]	Low	0	
	Tonga	[4.8, 5.05, 5.225]	Low	2	
C2, Medium SMR N = 34	Laos	[6.2, 6.9, 7.725]	Medium	0	Gender, mental disorders, population, predictors, prevention, risk factors, combat veteran, military, post-traumatic stress, trauma, veterans, violence, anxiety, children, depression, care, war.
	North Korea	[9, 9.15, 9.25]	Medium	15	
	Singapore	[8.125, 9.1, 10.075]	Medium	83	
	Thailand	[7.275, 7.65, 8.85]	Medium	52	
	Vietnam	[7.075, 7.3, 7.5]	Medium	145	
C3, Medium high SMR N = 29	Australia	[10.075, 10.4, 11.3]	Medium	1070	Age, alcohol, women, adolescents, children, prevalence, self-harm, anxiety, care, community, depression, population, symptoms, China, mental disorders, psychological autopsy, schizophrenia, reliability, validity.
	China	[7.325, 9.85, 12.575]	Medium	896	
	Fiji	[10, 10.2, 10.5]	Medium	18	
	New Zealand	[11.075, 11.95, 12.3]	Medium Hi.	0	
C6, High SMR N = 2	Kiribati	[31.575, 32.4, 32.925]	High	3	Risk, students, alcohol use initiation, cannabis, cigarette smoking, early substance use, epidemic suicide.
C8, Mixed medium and high SMR N = 36	Japan	[15.575, 18, 18.8]	High	770	Age, gender, unemployment, depression, health, internet, population, social support, care, community, education, elderly, self-harm, euthanasia, people, schizophrenia.
	Samoa	[14.8, 14.9, 15.3]	High	10	
	Solomon Islands	[17.35, 17.8, 18.25].	High	3	
C9, High SMR N = 12	Mongolia	[19.775, 22.55, 23.45]	High	5	Risk, students, alcohol, cannabis, loneliness, substance use, anxiety disorders, depression.
	Vanuatu	[21.175, 21.85, 22.275]	High	4	
C10, High SMR N = 7	Micronesia	[27, 27.4, 28.05]	High	6	Adolescents, children, depression, youth, age, economic crisis, gender, inequalities, unemployment, mental disorders, predictors.
	South Korea	[20.725, 22.35, 24]	High	360	

**Table 13:** SMR in South Asia geographic region.

Cluster, SMR Category	Country	[SMR Q25, SMR Median, SMR Q75]	SMR Label	WoS Records	VOSviewer Keyword Annotations
C1, Low SMR N = 56 n = 3	Bangladesh	[3.8, 4.95, 5.6]	Low	52	Asia, children, injury, verbal autopsy, violence, women, assault, domestic violence, intimate partner violence, pregnancy, anxiety, depression, demography, prevention, risk factors.
	Bhutan	[5.2, 5.3, 6.025]	Low Hi.	3	
	Maldives	[2.9, 3.25, 3.8]	Low	0	
C2, Medium SMR N = 34 n = 1	Afghanistan	[6, 6.75, 7.625]	Medium Lo.	303	Afghanistan war veterans, depression, PTSD, symptoms, US veterans, active duty, combat, deployment, mental health, military, resilience, soldiers, traumatic brain injury, terrorism, war, trauma, veterans.
C3, Medium high SMR N = 29 n = 2	Nepal	[9.8, 10, 10.425]	Medium	41	Attitudes, deliberate self-harm, poisoning, suicide bombing, terrorism, violence, mental health, stigma, women, anxiety, depression, medical students, stress, abuse, health, gender, low income, risk factors.
	Pakistan	[10, 10.25, 11.025]	Medium	111	
C8, Mixed medium and high SMR N = 36 n = 1	India	[13.35, 15.6, 17.025]	High Lo.	570	Agriculture, autopsy, Kaniyambadi block, pesticides, poisoning, rural India, adolescents, children, mental health, poverty, self-harm, violence, care, depression, domestic violence, gender, risk factors, schizophrenia, women.
C9, High SMR N = 12 n = 1	Sri Lanka	[14.575, 18.1, 21.95]	High	190	Asia, depression, women, developing world, pesticide poisoning, health, impact, ingestion, rural Sri Lanka, deliberate self-harm, pesticide, suicide prevention.

**Table 14:** SMR in Latin America and Caribbean geographic region.

Cluster, SMR Category	Country	[SMR Q25, SMR Median, SMR Q75]	SMR Label	WoS Records	VOSviewer Keyword Annotations
C1, Low SMR N = 56 n = 16	Antigua and Barbu.	[ 0.175, 0.35, 1.3]	Low	0	Alcohol, homicide, violence, women, care, mental disorders, population, prevention, abuse, adolescents, children, students, substance use, youth, anxiety, bipolar disorder, depression, prevalence.
	Bahamas	[3.2, 3.35, 3.42]	Low	1	
	Barbados	[0.3, 0.65, 1.225]	Low	1	
	Brazil	[4.9, 5.2, 5.45]	Low	300	
	Colombia	[3.875, 4.25, 4.55]	Low	54	
	Dominican Republic	[5.3, 5.5, 5.85]	Low	4	
	Grenada	[0.6, 1.35, 2.225]	Low	0	
	Honduras	[2.7, 3, 3]	Low	3	
	Jamaica	[1.975, 2.1, 2.2]	Low	21	
	Mexico	[4.3, 4.75, 5.4]	Low	282	
	Nicaragua	[4.975, 5.45, 5.95]	Low Med.	11	
	Panama	[3.475, 4.7, 5.9]	Low	1	
	Paraguay	[3.875, 4.65, 5.675]	Low	2	
	Peru	[2.8, 3.05, 3.225]	Low	21	
	St. Vincent and the Grenadines	[2.375, 4.8, 6.25]	Low	1	
	Venezuela	[2.375, 3.65, 4.875]	Low	12	
C2, Medium SMR N = 34 n = 7	Argentina	[8.4, 8.55, 9.05]	Medium	30	Behavior, bipolar disorder, depression, HIV, lithium, psychopathology, women, adolescents, aggression, gender, mental health, firearms, homicide, violence, health, impact.
	Belize	[6.9, 7.45, 8.125]	Medium	0	
	Bolivia	[7, 7.15, 7.425]	Medium	4	
	Costa Rica	[5.75, 6.7, 7.3]	Medium	6	
	Ecuador	[8.775, 9.35, 10.3]	Medium	12	
	El Salvador	[6.175, 6.9, 7.325]	Medium	8	
	St. Lucia	[7.1, 7.5, 7.9]	Medium	1	
C3, Medium high SMR N = 29 n = 5	Chile	[9.2, 9.7, 10.525]	Medium	74	Adolescents, behavior, care, health, age, mental health, euthanasia, life, mental health, depression, mental disorders, psychiatric disorders, women, children, predictors, violence.
	Cuba	[10.35, 10.65, 11.55]	Medium	23	
	Guatemala	[6.4, 9.1, 13.55]	Medium Wi.	4	
	Haiti	[11.925, 12.05, 12.525]	Medium Hi.	4	
	Trinidad and Tob.	[9.2, 10.45, 12.65]	Medium	8	
C6, High SMR N = 2 n = 1	Guyana	[35.75, 37.85, 39.825]	High	18	Adolescents, depression, forensic science, Guyana, Johnstown.
C8, Mixed medium and high SMR N = 36 n = 1	Uruguay	[13.875, 14.75, 17]	High	10	Aggression, alcohol drinking, bullying, childhood, middle school students, school health, substance use, criminal law, assisted suicide, cocaine base post, elderly, human rights, depression, intimate partner violence.
C10, High SMR N = 7 n = 1	Suriname	[25.9, 26.55, 27.075]	High	4	Teenagers, youth suicide, pesticides, Hindustan culture, psychological autopsy, economic policy, indigenous health, global health governance, mercury exposure.

**Table 15:** SMR in North America geographic region.

Cluster, SMR Category	Country	[SMR Q25, SMR Median, SMR Q75]	SMR Label	WoS Records	Keyword Annotations
C3, Medium high SMR N = 29 n = 2	United States	[10.775, 11.55, 12.525]	Medium	730	Alcohol, depression, mental disorders, population, substance use, adolescent suicide, firearms, gender, health, homicide, prevention, rates, violence, women, attitudes, Canada, care, euthanasia, life, children, stress, youth.
	Canada	[10.175, 10.35, 10.75]	Medium	3563	

## Data Availability

Data collected for this study and Python code used to analyze the collected data are publicly available on GitHub [28].

## Supplementary Materials

The following supporting information can be downloaded at: https://www.mdpi.com/article/10.3390/ijerph21091202/s1, Figure S1: Country clusters; Figure S2: Country clusters silhouette plot.
