# Supplementary material for: Global Suicide Mortality Rates (2000–2019): Clustering, Themes, and Causes Analyzed through Machine Learning and Bibliographic Data
Source: Int J Environ Res Public Health. 2024 Sep 10;21(9):1202. doi: 10.3390/ijerph21091202 (PMC11431541; doi:10.3390/ijerph21091202)
Supplement: Supplementary file 1 [file ijerph-21-01202-s001.zip › ijerph-3110525-supplementary/SUPPLEMENTARY/Supplementary_Document_S1_Keywords_across_gegographic_regions.pdf]

Keywords by region.

Europe and Central Asia region.

Table S5. Expanded outline of country-shared keywords in clusters of countries from Europe and Central Asia.

Cluster	Country shared most common keywords in the cluster
C2	Euthanasia; para suicide; Europe; mental disorders; age; seyle; religion; program; cohort; consumption; former USSR; alcohol consumption; schizophrenia; suicide attempts; general practitioners; drugs; abuse; neglect; Belgium; physician assisted suicide; Netherlands; palliative care; assisted suicide; of life decisions; Flanders; medical practice; law; ethics; of life practitioners; nurses; life; decision making; end of life care; Estonia; life events; smoking; suicide prevention; anxiety; youth suicide; depressive symptoms; psychopathology; Hungary; comorbidity; major depression; seasonality; help seeking; adolescents; birth weight; childhood; harm; perestroika; Baltic states; household dysfunction; chronic pain; mental disorder; unemployment; sex; stigma; overdose; delinquency;
C5	Russia; former USSR; Belarus; alcohol consumption; quality; vital statistics; classification; inequalities; age; suicides; Baltic states; smoking; time series analysis; all cause mortality; IQ; crisis; national intelligence; drinking; accidents; eastern Europe; central Asia; internet; heat; salafism; suicidal thoughts; suicide terrorism; temperature; infection; violent; weather; common mental disorders; developing world; HIV; pesticides; seasonality; sexual abuse; bullying; hanging; solar activity; exposure; students; record linkage; religion; diagnosis; Siberia; minors; crime; indigenous peoples; life expectancy; psychopathology; suicide rate; stress; abortion; international security; counter terrorism; national security; etno psychology; newspaper surveillance; anxiety; tryptophan
C6	Age; euthanasia; meta analysis; Europe; unemployment; self harm; sexual abuse; mental disorders; physician assisted suicide; patterns; stress; schizophrenia; follow up; smoking; psychiatric disorders; religion; suicide prevention; custody; prison suicide; national intelligence; genetics; sex; hypothesis; media; inheritance; inmates; war; pt sd; forensic science; deliberate self harm; southwestern Croatia; trace elements; DNA; forensic medicine; trauma; drinking water; personality; quality of life; health care; suicidality; drowning; intoxication; autopsy; GDP; illness; welfare state; adolescent; anxiety; mental health care; left behind children; behavioural problems; emotional problems; psychosocial health; parental migration; economic migration; alcohol dependence; eastern Europe; premature mortality; transition; seasonal variation; gender identity; delinquency; army; intimate partner violence; post traumatic stress disorder; sexual violence; serum cholesterol; environment; weather conditions; demography; assisted suicide; palliative care; right to die; firearms; of life decisions;
C7	Euthanasia; mental disorders; schizophrenia; unemployment; self harm; age; men; cohort; Sweden; cancer; bipolar disorder; major depression; young people; child abuse and neglect; violence against children; icast ch; eastern Europe; Russia; mental illness; palliative care; antidepressants; borderline personality disorder; economic crisis; overdose; abuse; para suicide; alliance; Europe; bereavement; war; delinquency; religion; dialysis; teenagers; withdrawal; child maltreatment; hellfire; chronic renal failure; problem behaviours; youth suicide; road deaths; electrocution; teachers; Muslims; completed suicide; quality of life; postnatal depression;

	recession; anger; austerity; Nordic countries; great recession; depressed mood; epilepsy; pt sd; crime; Durkheim; income; serotonin; impulsivity; of life practices; Oregon death; euthanasia law; cancer patients; bullying; slippery slope; terminally ill; hopelessness; social inequalities; deliberate self harm; stress; psychological autopsy; forensic science; inheritance; maltreatment; childhood; blood serotonin; parents;
C8	Euthanasia; age; schizophrenia; follow up; patterns; unemployment; mental disorders; assisted suicide; Europe; suicide attempt; palliative care; self harm; victimization; youth suicide; substance use; African American suicide; distress; accident; intimate partner violence; injury; social indicators; suicide prevention; interventions; verbal autopsy; stress; homicides; hopelessness; validation; forensic science; abuse; bipolar disorder; elderly; law; ethics; economic crisis; financial crisis; recession; legislation; antidepressants; paracetamol; para suicide; young people; adults; overdose; income inequality; natural resources; party systems; cross national prevalence; Taliban; transnational terrorism; narcotics; civil war ; suicide terrorism; conflict; violent death; socioeconomic factors; drugs; states; adolescence;
C9	
C10	Meta analysis, euthanasia, physician assisted suicide, Russia, neglect, perestroika, pesticides, eastern Europe, alcohol consumption, former USSR, maltreatment, forensic, physical abuse, refugees, pathways, affective temperaments, convicts, self immolation, young adults, burns, Muslim world, suicidal motives, childhood household dysfunction, cancer mortality, premature mortality, maternal mortality, cohort. Life expectancy, non suicidal self injury, individuals, smoking, college students, depression severity, job satisfaction, occupational stress, social policy, socio economic indicators, psychiatric nurses, hipa axis, healthcare reforms, cultural, nightmare, cerebrospinal fluid, tragedy, economic crisis, financial crisis, unemployment, austerity, crisis, recession, mental disorders, psychological autopsy, great recession, domestic violence, pesticide, opinion, persuasion, central Asia, political messages.

Table S6. Expanded Outline of country-specific keywords in clusters of countries from Europe and Central Asia.

Cluster	Country	iso3c	Frequent country specific keywords	Web of Science records	Female mean SMR	Male mean SMR
	**High SMR**	**countries**				
	Belgium	BEL	Health care institutions; epa; non voluntary euthanasia; cognitive validity; treatment planning; influence of religions; induced myocarditis; radical Muslim doctors; attack; continuous palliative sedation; voluntary assisted dying;	264	11.85	28.71
**C2**	Estonia	EST	Thyroid cancer; religious/spiritual aspects; internalized homo negativity; emergency workers; Baltic countries; severity of suicide attempt; pierce suicidal intent scale; Chernobyl nuclear accident; minisatelate mutation rate; urinary ethyl glucuronide; bio dosimetry components of suicidal intent; liquidators; gender and age differences; phosphatidylethanol;	48	8	36.57
	Hungary	HUN	Congenital abnormalities; Gabriel Tarde; female physicians; beck hopelessness scale; suicide attempt during pregnancy; abc schizophrenia; subthreshold diagnosis; pregnancy age; congenital toxoplasmosis; cognitive and behavioural development; latent toxoplasmosis; cats; self poisoning suicide attempt; subsyndromal depressive symptoms; very high doses of drugs; meteorological	128	11.04	38.64

			conditions; promethazine; suicide attempt during pregnancy;			
	Latvia	LVA	Latvia; suicide behaviours;	10	8.58	43.33
	Montenegro	MNE		6	10.89	31.17
	Slovenia	SVN	Heroin use disorder patients; register of intoxication; partial hanging; medical records; incomplete hanging; agonist treatment; heroin use disorder; violent suicide methods; primary care physicians; marital status change; heroin use; opioid agonist treatment; intentional self poisoning by drugs; cognitive functions; social and economic consequences of gambling;	70	10.17	37.78
	Belarus	BLR	1970-2005; alcohol psychosis; distributed lag analysis; rural urban population; direct evidence; inadmissible evidence; sources of evidence; alcohol psychosis rate; fatal alcohol poisoning; regional mortality; legal force of evidence; key evidence; para suicide, circumstantial evidence; assessment of evidence; USSR;	36	10.69	60.76
	Kazakhstan	KAZ	Kazakhstan; cognitive behavioural approach; positive women; sava syndemic; irrational attitudes; apparent temperature;	9	10.27	52.63
**C5**	Lithuania	LTU	Parties; quin tana; empirical Bayesian; maximal likelihood; suicide pcr; sense of coherence; line health professionals; relation with authority; lice; employment status influences; suicide intervention response inventory; cosmic ray; health of rural population; independent Lithuania; accessibility of healthcare; seasonal risk; young physicians; psychosocial adaptation; time pattern; oncology deaths;	67	12.99	66.97
	Russian Federation	RUS	Nonparametric estimates; inducing; incitement; facilitating; organizing activities; suicides; demography of rural associations; death rate; death rate from external reasons; depopulation; migrations; migratory politics; suicides among urban residents; federation; neurocognitive functions; programs for the prevention of suicides among minors; encouragement; psychic health; tyva; native peoples of Siberia;	28	12.88	70.61
	Ukraine	UKR	Psychological adaptation; power plant; big five; European social survey; economic background; discriminant analysis; legal conflicts; strategy of eradicating female terrorism;	24	8.27	50.65
	Austria	AUT	Regional differences; traumatic brain injury deaths; social interaction anxiety; rugged individualism; modes of reception; film effects; decomposition and mummification of corpses; medicals lays; attitudes towards euthanasia; index of inequality; tyrol; prevention classification description; telephonic utilization; long term care dependency; epidemiological findings; aquatic invertebrates; spatial regression models; directions; portrayals; characters;	158	8.04	25.78
	Croatia	HRV	Heavy metals; committees; icp; major elements; rainwater tanks; shell vial technique; phylogenetic analysis; Mediterranean spotted fever; serology; continental area; suicide motive; explosives; Zagreb epilepsy center; rickettsia conorii; bjelovar; youngsters; self harm behaviours; osijek; forms of violence;	52	8.82	28.17
**C6**	Czechia	CZE	Czech republic; socio demographic patterns; questionnaire seniors; possible improving of primary care; coal gas; burning barbecue charcoal; myocardium;	18	5.58	24.76
	Finland	FIN	Northern Finland; hospital discharge register; fagerstrom tolerance questionnaire; prevention theory; self destruction; living arrangements; alcohol associated suicide; global solar radiation; breast disease; h3 imipramine binding; physician communication; opiate users; rural suicide prevention and intervention; Nordic countries; physical disorders; pesticide use; Eastern Finland; high security care; adolescent parricide;	380	9.29	28.88
	France	FRA	Plague; french adolescents; psychiatry wfsbp guidelines; wold federation; orientals; maslach burnout inventory; HIV infections; clinical audit; mental suffering; school difficulties; organizations whoqil fref; panic attacks; resistant	415	9.95	25.68

		schizophrenia; fast dissociation; core; factorial structure; mixed depressive mania; patient's right; hospitalization under constraint; psychiatric emergency unit;			
Moldova	MDA		3	5.19	30.68
Poland	POL	8 year; standard expected years of life lost; political protest; exile; acute poisonings; isomorphism; business ethics; adolescent marijuana; ambiguities; small and medium sized companies; polish Catholicism; Catholics; writer; process; satanism; r flip; taq1a polymorphism; environmental contributions; post treatment relapse; communism;	90	4.55	29.3
Serbia	SRB	3,5 dimetoxyphenol; social conditions; suicide in Serbia; responsible media reporting; invisible victims; code of ethics of Serbian journalists; vojvodina; hand grenades; psychophysiological disorders; explosive wounds; prison environment; crime victims; paclitaxel; plant toxins; yew leaves; chronic anxiety; taxus baccata; mental health inventory; propensity score matching; mortality trend;	35	9.41	25.79
Slovak Republic	SVK		5	3.76	23.32
Switzerland	CHE	Geneva; futility; social professionals; succession; ed users; suicide regulation; families; intravenous drug use; sever and persistent mental illness; treatment resistance; assessment of suicide; assessment instruments; visual instrument; suicidality assessment; maintaining hope; residential distance; health care cost; health insurance; rates of suicide; unassisted suicide;	284	10.91	22.91
**Medium SMR countries**					
Bosnia and Herzegovin a	BIH	Bosnia and Herzegovina; compassionate care; Sarajevo; emo subculture;	6	4.45	16.65
Bulgaria	BGR	Suicidal index; unemployment level; self injuries;	13	6.07	19.61
Denmark	DNK	Danish; 80 and over; life mission theory; vaccinia; shelters; outcome predictors; population study; nested case control design; IVF treatment; population attributable risk; civil registration system; qol; sickness absence; holistic process theory; premature mortality; psp cooperation; radicalization awareness; psychosocial therapy;	340	7.87	18.1
Germany	DEU	In patient suicide; open door policy; german health interview; medical clearance; e mental health; prison system; coercive measures; motivated reasoning; cranio-cerebral missile injuries; punitiveness; socio-emotional selectivity hypothesis; ingroup; dialectical behavioural therapy for adolescents; harm behaviour; german history; standard diagnosis; telephone survey;	462	6.96	19.9
Iceland	ISL	Iceland; friendship networks; rotorua; hazard assessment; census based cohort; community context; women's health issues; birth registration; serious adverse effects; perinatal care; delinquent peers; family conflict; discharge diagnosis; cause specific death; practice patterns; anger expression; attendances; quasi Poisson model; century; contextual effects;	32	6.66	19.36
Ireland	IRL	Ganciclovir; spect; gc/ms; planned complex suicide; Iowa gambling task; bystander effect; self regulation; forensic pathology; prolactin; lipid levels; mesenchymal stem cells; chain saw; toxicological analysis; malignant glioma; glucose metabolism; striatum; induced apoptosis; cytosine deaminase gene; nitric oxide; interactive model;	1258	4.64	18.51
Kyrgyz Republic	KGZ		1	4.3	18.71
Luxembour g	LUX		18	7.47	18.29

C7

	Norway	NOR	Tension type headache; Norwegian adolescents; baerim; nord trondelag health; co rumination; sapmi; psychopathological symptoms; conduct difficulties; leaving against medical advice; repeated poisoning; nephropathy; recurrent headache; attention difficulties; farm related concerns; drug consumption; hordaland health; injury and severity score; farm economy; job stress; impaired physicians;	238	7.62	17.19
	Portugal	PRT	Water supply; spatial determinants; winter; anorexia nervosa seawater; ion batteries; length of hospital stay; utilization; economic crisis; late onset schizophrenia; children depression; electric vehicle; industrial use; drinking water lithium; granitic pegmatites; specialized care;	66	5.68	18.78
	Romania	ROU	Intimate partner homicide suicide; co morbidities; residential injuries; diazinon; heme; tki inhibitors; dentist; coping strategy; supra expression; eml4 alk fusion gene; children left behind; multiple victims; enolase levels; professional work stress; methomyl; cholinesterase inhibitors; current suicide risk;	66	3.93	20.49
	Sweden	SWE	International adoption; neurodevelopmental disorders; 4.9 inhabitants; childhood socioeconomic position; Northern Sweden; passenger car fatalities; bereaved relatives; suboptimal health; lone mothers; multiple myeloma; criminal victimization; international adoptees; social assistance; childhood IQ; entrepreneurs; gestational age; female offender; convergent validity; grades;	558	9.49	20.71
	Georgia	GEO	Internalizing symptoms; multi drug resistant tuberculosis; virtual patient; alcohol attitudes and alcohol education; city substance users; civil registration; hepatic c virus; suicide thoughts and behaviours; communication checklist; age in grade; emotional disturbance; non natural death; attitude towards euthanasia; medical examiner investigator; forensic pathologist; referral patterns;	32	3	14.15
	Italy	ITA	Psychiatric reform; illicit drug overdose; drugs of abuse; stent graft; biological rhythms; northern Italy; glass; sensation seeking behaviour; durkheimian suicide analysis; homicidal deaths; defence wounds; self deception; denial; adolescent behaviour; emotional lability; somatic disorders; Italian suicide rates; current use; withdrawal life prolonging treatment;	296	3.5	10.87
	Netherlands	NLD	Train the trainer; e learning; social drift; physician aid in dying; living wills; ethnic minority density; pharmaco epidemiology; hastening death; general public; family medicine; nesda; mental imagery; withdrawal of life support; wish of hastened death; sedation until death; incident data; stations; accident analysis; rail suicide;	639	6.78	14.08
**C8**	North Macedonia	MKD		0	4.97	13.85
	Spain	ESP	Andalusia; policy makers; urban areas; geographical inequalities; synthetic cathinones; psychiatric emergency services; rapid assessment; drug intoxication; medicine intoxication; vitreous humor alcohol concentration; canary islands; genetic association; increased impulsivity; psychiatric diagnosis suicide method; late 2000s; standardized rates; panel surveys; home eviction;	298	3.91	12.06
	Turkmenistan	TKM		0	4.16	16.01
	United Kingdom	GBR	Gender queer; therapeutic landscape; 1,4 butanediol; political terrorism; stimulus valence; pastoral care; counselling in higher education; limitation; assisted reproduction; oocyte aspiration; ssri prescriptions; group processes; analgesic method; inter-group discrimination; restriction of access; Scottish; national representative study;	253	3.98	12.57
	Uzbekistan	UZB		2	4.81	12.99

	**Low SMR**					
**C9**	Turkiye	TUR	Shelter; Adana; chronotype; dao; authoritarian; self injury behaviour; incest; mobbing; Istanbul; symptoms sharing; alcohol treatment; relapse prevention; treatment motivation; cognitive flexibility; physiotherapists; sociodemographic characteristics; adult adhd; sport participation;	324	1.39	3.93
	Albania	ALB		12	4.16	7.55
	Armenia	ARM	Personality peculiarities; character accentuation; penitentiary institutions;	2	2.61	7.27
	Azerbaijan	AZE	Electronic device; student's mental health; teacher form; parent form;	4	1.72	6.72
**C10**	Cyprus	CYP	Workplace empowerment; colonial policies; goats; Mediterranean degradation; imagery re-scripting and reprocessing therapy; intrusive memory; early maladaptive schema;	9	1.56	6.05
	Greece	GRC	Health system; multiple correspondence analysis; Greek crisis; commons; phone counselling; conium maculatum; impact of financial crisis; quasi experimental approach; long term conditions; language shift; arvanitika; Greek; language community; marital quality; lunar periods; cyclicity; epa guidance; psychosomatic aspects; female aborigines;	146	1.48	6.72
	Tajikistan	TJK		2	2.53	5.23

Sub-Saharan Africa region

Table S7. Expanded outline of country-shared keywords in clusters of countries from Sub-Saharan Africa.

Cluster	Country shared most common keywords in the cluster
C2	HIV/AIDS; stress; disorder; injuries; primary care; students; men; stigma; substance use; psychiatric disorders; alcohol use; suicide prevention; intimate partner violence; internet searching; humanitarian injustice; violent death rate.
C7	Anti-colonialism; service provision; terrorism; CIA; suicide bomber; heteronormativity; criminalization; sexual and gender minorities; mental distress; school children; carbon dioxide; boko haram; firearm injuries; asphyxia deaths; thiosulfate; poisoning; h2s poisoning; co2 poisoning; death in geothermal area; pulmonary surfactant apoprotein; residues; cause of death; forensic autopsy; infectious diseases; histological analysis; asylum applicants; antenatal depression; sexual and reproductive health and rights; struggle; middle income countries; liberation; anxiety; drug use; intimate partner violence; sexual violence; illicit drug use; LGBT; tobacco use; psychosocial distress; tobacco; euthanasia; sexually transmitted diseases;
C8	HIV Africa; trauma; aids; terrorism; women; pathogenesis; immune system; outbreak; spongiform encephalopathy; polyene antibiotics; prion protein; RNA; live vaccine strain; suppression subtractive hybridization; immunoglobulin g antibodies; incubation; interferon; pathogenicity; vibrio cholera; community; anxiety; adults; students; domestic violence; Palestine; CIA; anti-colonialism; stress; war; exposure; psychological autopsy; post traumatic stress disorder; medico-legal death; neuropsychiatry observations; neuroscience; funding; firearm deaths; gunshot; epilepsy; acute poisoning; suicide bombing; schizophrenia; mental disorders; alcohol use disorders; clinical course; psychology students; nurses; homicide suicide; religion; decriminalization; suicide victims; ethanol; brain; rat; hippocampus;

	phosphodiesterase; mitochondria; frontal cortex; antirational therapy; carers; intellectual disability; depressive illness; criminalization; socio economic factors; heteronormativity; partner violence; pregnancy; genocide; women's health; psychometrics; multi-country; refugees; sexual violence; alcohol consumption; loneliness; coping strategies; school health; military personnel; veterans; operations; force; blast injuries; terrorist bombings; explosions; torture; us veterans; psychiatric; Persian gulf war; para suicide; corporal punishment;
C10	Primary care; terrorism; HIV; validation;psychiatric disorders; comorbidity; validity; schizophrenia; scale; stigma; community; income; poisoning; suicide bombing; boko haram; deliberate self harm; suicidality; aids; knowledge; young people; verbal autopsies; experiences; indigenous knowledge; male infertility; psychosocial impacts; apoptosis; positron emission tomography; suicide victims; major depression; inhibition; infection; mono amine oxidase; ethanol; rat brain; CIA; anti colonialism; hippocampus; phosphodiesterase; mitochondria; frontal cortex; mental disorders; schizo-affective disorder; interpersonal theory; corporal punishment; alcohol use; marital status; suicide ideation; anxiety; clinical attitudes; non specialists; treatment gap; intersectionality; framework; issues; psychosis; strategies; availability; intervention; therapist competence; forensic science; cytotoxic cardenolide glycoside; cardio toxicity; cardenolide; cerberin; cardiac glycoside; cerber addollam toxicity; plant poison; odollam tree; ordeal; apocynaceac; plant; digoxin; tree; emergency medicine; forensic toxicology; seeds;war; drug; emergency;violent death rate; internet searching; humanitarian injustice; help seeking; disability; beliefs;adult mortality; ingestion; epidemic; Escherichia coli; suicide vector; paraphenylene diamine; adolescent suicides; abuse; hope; exposure; developing world; intimate partner violence; street youth; threat; urban rural differences; non communicable disease; life style.

Table S8. Expanded Outline of country-specific keywords in clusters of countries from Sub-Saharan Africa.

Cluster	Country	iso3c	Frequent country specific keywords	Web of Science records	Female mean SMR	Male mean SMR
	**High SMR countries**					
**C1**	Lesotho	LSO		0	25.24	95.59
	Botswana	BWA		0	12.01	40.12
**C2**	South Africa	ZAF	Non-fatal suicidal behaviour; socioeconomic context; Pretoria; suburban; calcaneus; southafrican blacks; femur; self harm social support coping; community based organizations; St. Johns wort; tension reduction coping;	232	10.87	37.99
**C3**	Eswatini	SWZ		0	19.5	64.5
	Cabo Verde	CPV		0	4.74	22.71
**C6**	Central African Republic	CAF		1	6.96	23.99
	**Medium SMR countries**					
**C7**	Cameroon	CMR	Vesicovaginal fistulas; health district;	9	5.35	15.53

	Cote d'Ivoire	CIV		0	3.88	18.15
	Eritrea	ERI		1	5.7	19.44
	Gabon	GAB		0	3.68	16.48
	Mozambique	MOZ	North Carolina;	9	6	21.69
	Namibia	NAM	Namibia;	5	5.69	22.91
	Togo	TGO		3	5.03	15.39
	Zambia	ZMB		0	5.61	14.9
	Zimbabwe	ZWE	Lysosomes; protein crystal structures; lay health workers; Harare; traditional medicine; urban Zimbabwe; HIV test; saposinb; activator protein; coenzyme q10; lifetime diagnosis; positive adolescents; postnatal suicidal ideation; standardized patients;	21	13.42	20
**C8**	Angola	AGO		0	2.93	11.77
	Benin	BEN	Going adolescents; Philippine;	7	4.26	12.61
	Burkina Faso	BFA	Medical emergency; Ouagadougou;	2	4.39	11.66
	Burundi	BDI		0	4.9	11.29
	Chad	TCD		2	3.72	10.99
	Congo, Dem. Rep.	COD		1	3.12	11.23
	Congo, Rep.	COG		10	5.92	12.17
	Equatorial Guinea	GNQ		0	5.87	12.6
	Ethiopia	ETH	Neglected tropical disease; non filarial elephantiasis; problematic khat use; severe mental disorder; roof less-ness; verbal; physical; suicide prison; glycemic control; treatment naive; traditional society; prevalence pattern; cause death; low and middle income country; adolescent males; treatment nonadherence; podoconiosis; enacted stigma; felt stigma;	52	4.44	9.73
	Ghana	GHA	Funeral rites; moral infraction; disabled women; northern region; health assessment and care; women in Ghana; sleep disruption; Ghana newspaper; media depictions; research capacity; science policy; research focus; neurogenomics; anything related to sexual assault; street children and adolescents;	53	1.76	12.11
	Guinea-Bissau	GNB		1	4.47	12.17
	Malawi	MWI	Implementation science; patient health questionnaire 9; rural Malawi; chi square tests; male circumcision; service delivery; psychological aspects; orphans and vulnerable children; HIV sell test; social harms; status disclosure; serostatus; hiv prevention;	12	3.2	11.67
	Mauritius	MUS		0	3.96	14.33
	Rwanda	RWA	Rwanda; 4 developing countries; Thai Burma border; reporting questionnaire srq 20; sensitivity and specificity; women’s health services; non psychotic mental health disorders;	6	4.81	11.46
	Seychelles	SYC	Suicidal expression;	1	1.61	14.62
	Somalia	SOM	UN peacekeepers; foreign bodies; bomb fragment; Somali;	7	3.99	12.84

	Uganda	UGA	Kampala; post conflict northern Uganda; distancing; qualitative psychological autopsy; African refugee settlement; women suicide; comorbidity; war conflict; social science research; a developing country; post secondary education; a choli; serotonin transporter (5htt) gene polymorphisms; vulnerable youth; parental neglect; trading sex; mental health intervention;	70	2.87	11.31
	**Low SMR countries**					
C9	Mauritania	MRT		1	2.4	3.99
	Sao Tome and Principe	STP		0	0.85	2.11
	Comoros	COM		0	4.05	6.92
	Gambia	GMB		1	3.46	7.37
	Guinea	GIN	Guinea pig; ototoxicity; 2 bromoethylamine; imipramine binding sites; acylpeptide hydrolase; vestibular sensory epithelia; clorgyline; caspase; cell lines; constitutive activity; gamma s binding; inverse agonist properties; human ventricular cardiomyocytes; g protein activation; intrinsic activity; gtp gamma s binding; 5 ht1a receptor; serum albumin; axonal transport; mass spectrometry;	30	4.58	8.46
	Kenya	KEN	Life questionnaire; emotional loneliness scale; family discord; kilifi health; faith healer; language version; subjective social standing; suicide risk referral; suicide risk management; accident and emergency nurses; suicide self efficacy; healers; clinical officers; extinction; mental health related outcomes; mhgap ig; symbolic emergency; symbolic suicide; attachment theory;	23	3.23	8.95
	Liberia	LBR	Illness narratives	2	3.87	5.64
	Madagascar	MDG		3	3.45	7.91
C10	Mali	MLI	Mali; massive influx; university hospital Gabriel Toure; deliberate poisoning;	4	3.47	5.47
	Niger	NER	Thermodynamics; biophysical chemistry; radio lysis; oxidation; metal ions; tartrazinefd; electron scavengers; catalasel mysteries; suicide inactivation; aspergillus noger; tartrazine dye; candida albicanis; tyrosinase bismuth oxide nanoparticles; penicillium chrysogenium; access channel; glutathione peroxidase; bovine liver catalase; clakoxygen electrode;	3	3.4	7.15
	Nigeria	NGA		52	2.45	6.17
	Senegal	SEN	Hdss; kersa health;	1	3.51	10.2
	Sierra Leone	SLE		1	4.44	8.18
	South Sudan	SSD		0	2.03	5.55
	Sudan	SDN	Angio edema; Sudan; co regulated pilus; ctx phi; colonization factor; el tor; transcriptional activation; environmental strains; pandemic strains;	5	2.97	5.14
	Tanzania	TZA	Low income country; school bullying; implementation science; sentence completion; in school adolescents;	14	2.97	7.78

Middle East and North Africa region

Table S9. Expanded outline of country-shared keywords in clusters of countries from Middle East and North Africa.

Cluster	Country shared most common keywords in the cluster
C8	Community; co proxamol; age; deaths; psychotropics; psychiatric hospital; propoxyphene; fatal poisonings; abnormalities; risks; deficit hyperactivity disorder; adulthood; methadone; developing world; ethnicity; substance abuse; sex; antidepressants; amitriptyline; burnout; euthanasia; cross national research; interpretative phenomena; employee; strikes; enteral nutrition; hunger; psychologists; empirical study; opinion; European values; attitudes of general public; coping strategies; ill cancer patients; terrorism; poisoning; schizophrenia; liaison psychiatry; suicide attempts; criminality; domestic violence; suicide plan; organophosphates; jihadism; ethnic identity; al-qaeda; para phenylenediamine;
C9	Terrorism, young adults, non communicable diseases, Muslim world, mental disorders, social support, suicide ideation, war, slimanebenaissa, tahar djaout, fundamentalism, conversion, deadly traffic injuries, maternal and child health, gap analysis, health development targets, sustainable development, transnational terrorism, jihad, social exclusion, political violence, al-qaeda, democracy, domestic violence, forensic science, trauma, psychosis, schizophrenia, impulsivity, disaster, tramadol, intimate partner violence, women, s mental health, rural Bangladesh, parturition, unwanted pregnancy, child bearing, college students, Islam, psychological distress, life events, childhood household dysfunction, cross cultural comparison, parental death, attachment, coping, suicidal motives, medical inpatients, politics of education, optimism, euthanasia, students, end of life, assisted suicide, mercy killing, bullying victimization, protective factor, indigenous population, small area, illicit drug use, neighbourhood, multilevel, phenomenology, suicidal behaviours, initiation, living conditions, violence prevention, psychiatric morbidity, Lebanon, anxiety, burnout, stress, validation, overdose, suicide bombing, emotional intelligence, us veterans, self esteem, Lebanon war, illness, ISIS, health effects, infectious diseases, histological analysis, asylum analysis, public interest, strait, law limits, counter insurgency, forensic cases, Mindanao, apocalypse, conspiracy, predominant party systems, opposition failure, erdogan, security informatics, hybrid warfare;
C10	Trauma, injuries, burns, suicide attempt, mental disorders, management, overdose, post traumatic stress disorder, terrorism, schizophrenia, suicide ideation, eastern Mediterranean region, burden of disease, self immolation, poisoning, inflicted burns, age, immolation, military, deployment, traumatic brain injury, combat, resilience, war, mental health problems, military personnel, soldiers, comorbidity, us veterans, service, exposure, adolescence, immigrants, social support, stress, suicide attempt, anxiety, suicide prevention, counter terrorism, self harm, local law enforcement, conceptual model, Fatimid architecture, Jean Raymond Pacho, collaboration, deadly traffic injuries, maternal and child health, gap analysis, health development targets, sustainable development, counter terrorism, non communicable diseases, acculturation, epilepsy, gun ownership, intoxication, cohort, availability, marital status, sex, harm, para suicide, anticonvulsants, focus groups, community, suicidality, sleep, melatonin, life style, consensus statement, professional football, midlife, sleep loss, Arabs, Saudi Arabia, autopsy, autonomy, young adults, college students, religiosity, forensic science, drug overdose, neck structures, spirituality, high school students, pediatric burns, cyberstalking, medical, cybercrime, migrant workers, decriminalization, cyberbullying, religion, students, legal analysis, outcomes, moderate Muslims, pluralism, moderate Islam, humanitarian injustice, internet searching, violent death rate, intentional injuries, experiences, systematics analysis, conflict, child abuse, maltreatment, consequences, disability, sexual abuse, nahdlatul ulama;

Table S10. Expanded Outline of country-specific keywords in clusters of countries from Middle East and North Africa.

Cluster	Country	iso3c	Frequent country specific keywords	Web of Science records	Female mean SMR	Male mean SMR
	**Medium SMR countries**					
C8	Bahrain	BHR	Bahrain; irrational use; prescribing pattern;	3	2.97	11.98
	Djibouti	DJI		0	5.52	10.79
	Malta	MLT		3	2.49	10.25
	Morocco	MAR	Hermine; abortifacient; vaccine; peganum harmala I.; souss massa draa; university hospital center; language maintenance; language contraction; imazighen (Berbers); harmful use; tunnissian sample; obstetric complications; idiopathic epilepsy; Hodgkin lymphoma; article 475;	24	6.3	11.15
	**Low SMR**					
C9	Algeria	DZA	Algeria; agency theory; kabylie; sociologic; firms; aqmi; institutional analysis; Maghreb; authoritarian institutions; strategic logic;	11	2.46	4.16
	Egypt, Arab Rep.	EGY	Addict; auto aggression; problem; fang marks; perceptions of domestic violence; suicidal and homicidal poisoning; dog; mummies; female death; post mortem examination; port said; soluble gas; lymphocyte apoptosis; prognostic significance; fas I; hcc; fas ligand; assiut;	24	2.22	4.1
	Jordan	JOR	Seaside resort; Neil Jordan; contemporary Irish fiction; archipelagic; community based research; ethical issues in research; Jordan;	11	1.03	2.53
	Kuwait	KWT	Parental smoking; teaching effectiveness; community instability; bedouin;	12	1.05	3.74
	Lebanon	LBN	Syrian crisis; trans feminine; south Lebanon; Australian veterans; Swedish UN soldiers; operation desert storm; well being assessment; multiple terrorist attacks; mena; bedouin; Elias Khoury; Rabi Jabir; terrorist modes of attack; intellectual Beirut; parody; metafiction; postwar Lebanon; cross sex hormones;	35	1.89	3.67
	Syrian Arab Republic	SYR		18	0.61	2.48
	Tunisia	TUN		0	2.26	4.68
C10	Iran, Islamic Rep.	IRN	Khozestan province; psychiatric distress; Iranian kurds; adjustment disorders; fars; chronic exposure; Kermanshah; visitation; religions; sprague dawley rats; burns percentage; upper extremity; subcutaneous injection; household insecticide; imprisoned mothers; caring; control counties; measurement model; safe; standard regimen;	259	4.37	8.62
	Iraq	IRQ	Focused attention; mass casualty response; pacific rim; stepped care; assault victims; transfusions; air transport; reintegration; community reintegration; sense of community; Amis; storm; desert shield; suicide bombing attack; operation; effectiveness research; chemical and biological casualties; typhomalarial fever; religious cleavages; suicide register;	379	2.43	5.28
	Israel	ISR	Hamas; second intifada; Arab; assassinations; mental health distress; biblical psychology; Palestinians; jet rempi; electrical state; semi arid areas; organo phosphate; online counselling; ground theory; cloninger; posters; treason;	248	2.53	9.03

		collaborators; wars; antidotal treatment; will to live;			
Libya	LBY		6	3.09	7.12
Oman	OMN	Oman; anti epileptic drug; Arab Islamic;	4	1.37	8.73
Qatar	QAT	Qatari women; suicide scales; menopause; Arabic women; cultural differences; incontinence; geomagnetic fields; apogee; perigee; nocturnal activity; bites; field;	4	2	9.19
Saudi Arabia	SAU	Skin disease; disease risk; index durel; psychometric assessment; umra worship; psychology of worship; religious rituals; cyberterrorism; worrisome; psychology of religion; Arabia; young age; Saudi Arabian province; ksa; mood disorder; small firearms; physician patient relations; poly drug abuse; beck depressive inventory; firearm forensic aspects; firearm injury incidence;	31	1.71	7.58
United Arab Emirates	ARE	East Asian immigrants; nationals; expatriates; Dubai; child bearing age; pelvic floor dysfunction;	7	3.29	9.31
Yemen, Rep.	YEM	Hadhramaut university students; Yemen;	6	4.68	6.93

East Asia and Pacific region

Table S11. Expanded outline of country-shared keywords in clusters of countries from East Asia and Pacific.

Cluster	Country shared most common keywords in the cluster
C2<br>C4<br>C6	Suicide attempt; epidemic suicide; Vavau; community psychiatry; young people; students; global school based health survey; early substance use; alcohol use initiation; nationally representative sample; substance use; suicidal ideation; unemployment; age; mental disorders; youth suicide; predictors; suicide prevention; schizophrenia; social support; scale; internet; community; self harm; intervention; elderly; education; euthanasia; social change; blood pressure; ethno cultural aspects; emotion; Korean adolescents; turbidity; cod; domestic sewage treatment; high school students; economic crisis;
C7	Community; Australia; euthanasia; self harm; deaths; help seeking; England; new south wells; suicide prevention; unemployment; anxiety; intervention; wales; mental disorders; follow up; young people; stress; suicide attempts; age; indigenous; Samoa; cannabis;adolescence; intimate partner violence; south pacific; outcomes; colonialism; institutions; Micronesia; community psychiatry; risk behaviour survey; social change; blood pressure; suicidal ideation; speaking; china; alcohol use initiation;
C8	Mental disorders; Hong Kong; social support; age; schizophrenia; validity' reliability; predictors; Chinese; para suicide; deliberate self harm; Asian; behaviours; psychological autopsy; community; anxiety; hopelessness; rural china; quality of life; impulsivity; culture; Fiji islands; attempted suicide; developing countries; general hospital; surveillance; Micronesia; drug; poisoning; illness; blood pressure; Hinduism; community psychiatry; refugees; migration. North Korea; spatial diffusion; south Korea; north Korean defectors; adolescent; asylum seekers; psychiatric symptoms; clusters; protective factor; social connectedness; middle east; Toronto

	alexithymia scale; soviet union; event scale; socialism; subjectivity; Singapore; suicide attempts; stress; adolescent suicide; harm; therapy; elderly suicide; older adults; intimate partner violence; major depressive disorder; loneliness; outcomes; meta analysis; psychological distress; severity; dsm iv; psychotherapy; unipolar; suicide ideation;
C9	Thailand, suicide ideation, psychological distress, patterns, Hong Kong, alcoholism, lifetime risk, time trend analysis, terrorism, suicide terrorism, students, validity, sample, smoking, older adults, college students, young adults, jihad, suicide attempt, Islam, suicidal behaviours, substance use, the association of southeast Asian nations, soft drink consumption, experience, self harm, ultimate sacrifice, medical anthropology, self sacrifice, injury pattern, ecology,, social dominance orientation, warfare, destructive killers, motivational primacy, sacred values, individual self, group identification, identity fusion, perceived collective continuity, rites of terror, imagistic practices, hopelessness, loneliness, guidelines, domestic violence, adolescent health, drug use, psychosocial distress, mental health first aid, birth weight, post traumatic stress disorder, menstruation, fetal growth, early substance use, s mental health, menstrual cycle;
C10	Instrument, Hopkins symptom checklist 25, depressive symptoms, services, childhood trauma questionnaire, sex work, hiv, suicidal expressions, physical activity, adolescent suicide, young people, media, global burden, substance use, antiretroviral therapy, college students, social support, suicide ideation, hopelessness, drug, income, religion, poisoning, students, dsm iv, young adults, stress, self harm, age, older adults, qualitative, area, psychosocial factors, refugee, bullying victimization, pregnant women, food, depression symptoms, university students, religion index, the association of southeast Asian nations, soft drink consumption, psychosis, community psychiatry, post traumatic stress disorder, Vietnam veterans, military, war, exposure, combat veterans, Persian gulf war, combat, scale, traumatic brain injury, mental health problems, suicide attempts;

Table S12. Expanded Outline of country-specific keywords in clusters of countries from East Asia and Pacific.

Cluster	Country	iso3c	Frequent country specific keywords	Web of Science records	Female mean SMR	Male mean SMR
	**High SMR countries**					
C2	Japan	JPN	Mie prefecture; Akita prefecture; contingent valuation survey; medical residents; depressive state; electromagnetic field; defeat depression campaign; national policy; follow up survey; hydrogen sulphide suicide; the great east japan earthquake; adenosine deaminase deficiency; geomagnetic storm; gender difference; month; Wiskott Aldrich syndrome; Asia pacific war; eudaemonia; public awareness campaigns;	770	12.65	31.68
	Korea, Rep.	KOR	Republic of Korea; solar radiation; time trend; kyrbws; crescent; sexual intercourse; sociotropy; status integration; suicide in south Korea; mediated moderation; individual and social stress; effect; death system; style inventory; personal coping resources; education program; under counting; game addiction; mental patients;	360	16.98	36.45

	Micronesia, Fed. Sts.	FSM	Oceania; Truk;	6	11.4	38.54
	Mongolia	MNG	Mongolia;	5	7.79	36.05
**C4**	Kiribati	KIR		3	9.53	51.31
**C6**	Solomon Islands	SLB		3	1.82	27.56
	Vanuatu	VUT	Cultural health beliefs;	4	7.93	29.18
	**Medium SMR countries**					
**C7**	Australia	AUS	Western Australia; aboriginal and Torres strait islander; workforce; remoteness; psychiatric injury; veterinarians; 2007 national survey; frailty; rural Australia; cop; big dry; chronic callers; suicide prevention center; community treatment orders; cultural and linguistic diversity; companion animals; improve; territory;	1070	5.78	17.73
	New Zealand	NZL		0	6.36	18.56
	Samoa	WSM	Status inconsistency; developmental niche; westernization; psycho neuroimmunology; acute phase proteins; urban settings; infancy; biological sensitivity;	10	6.57	18.6
**C8**	China	CHN	Psychological strain; fox conn; negative life event; micro-blog; rural China; Chinese workers; young suicide; weibo; republic of china; Guangzhou; one child policy; celebrities; territories; Taipei city; strain theory of suicide; health system reform; Chinese rural elderly; Chinese population; epidemiological profile	896	9.25	11.7
	Fiji	FJI	Trauma registry; condoms; social learning theory; motor vehicle assisted suicide; pacific island; wound and injury; middle income; Fiji islands; islands;	18	6.34	12.85
	Korea, Dem. People's Rep.	PRK	Cold war; Czechoslovakia; Karlovy Vary; national missile defense; Korean American; Korean nuclear crisis; us foreign policy; rational deterrence; liberal internationalism; extended deterrence; Korean independence movement; Alice Hyun; Wellington Chung;	15	8.28	10.86
	Singapore	SGP	Academic stress; adolescent outpatients; catatonia; train suicide; meaning centered ethnography; ethnographic writing; sociodemographic profile; pedagogics and literacy; cultural genres; intercultural theatre; lethality of means; art du de placement; efflux; Malays; child and adolescent suicide; funeral ritual; Chinese diaspora; clinical diagnoses; parental invalidation; health utility;	83	7.35	13.77
	Thailand	THA	Thai; Thai adolescents; cardio toxic ornamental plant species; acute organophosphate; public secondary school; southern border provinces Thailand; occupational heat stress; secondary school students; euro d scale; preoccupied; anxious; action research; Bangkok; suicide tree; ITS DNA markers; fatal firearm injury; primary care unit; intervention program; deletion;	52	4.07	14.05
	**Low SMR countries**					
**C9**	Brunei Darussalam	BRN		1	0.83	3.32
	Indonesia	IDN	Bali; Islamist; emotional loneliness; terrorism and social media youth and terrorism; lone wolf terrorism; Indonesian millennial; counter narratives; online radicalization; female jihadist; spiritual messages; high risk behaviour outcomes; health services assessment; kessler 6 screen; community mental health service; banten; muhammadiyah; Islamic radicalism; lottery; cash;	32	1.39	4.14

			majlis taklim;			
	Papua New Guinea	PNG		6	1.38	3.76
	Philippines	PHL	Levodopa equivalent dose; Parkinson disease; postnatal growth; subthhalamic nucleus; pallidotomy; tremor; pd; birth length; Cebu cohort study; peer counselling; education; campus based;	28	1.08	3.09
	Timor-Leste	TLS		0	2.06	4.44
	Cambodia	KHM	Women's health; key somatic complaints; cultural syndromes; alcohol advertising; media exposure; societal attitudes; diet; nets; clinical progression; developing world bioethics; empirical ethics; rationing treatment; scarce medical resources;	19	3.32	7.23
	Lao PDR	LAO		0	4.31	7.9
	Malaysia	MYS	Suicide registry; chrysomya megacephala; healthcare team; health nurses; chemical; human robot interaction; robotic therapy; mediation role; young gay; national health and morbidity survey; Penang public hospital; smoking and alcohol use; Kuala Lumpur; youth development; cyber aggression; cyber victimization; positive orientation; psychological distress scale; suicide coverage;	77	2.16	7.17
**C10**						
	Myanmar	MMR	Migrant; Myanmar;	7	1.67	5.78
	Tonga	TON		2	2.91	5.56
	VietNam	VNM	Post service mortality; Vietnam theatre veterans; academic pressure; bone fragments; adolescent offspring; Vietnamese adolescents; low mood; venlafaxine extended release; variable number tandem repeat (vntr); external mortality; malignant neoplasms; Chinese women; urinary cortisol excretion; delayed onset; psychiatric cost; Davidson trauma scale; prisoners of war; captivity; prolonged exposure therapy; betrayal trauma;	145	4.44	9.47

South Asia region

Table S13. Expanded outline of country-shared keywords in clusters of countries from South Asia.

**Cluster**	**Country shared most common keywords in the cluster**
C6	Pesticides; self harm; developing countries; suicide prevention; poisoning; self poisoning; harm; tragedy; ingestion; strategies; pesticide poisoning;
C7	India; deaths; Tamil Nadu; south India; self harm; poisoning; china; developing countries; Sri Lanka; mental disorders; schizophrenia; deliberate self harm; suicides; domestic violence; kaniyambadi block; poverty; agriculture; primary care; pesticides; attempted suicide;
C8	India; self harm; anxiety; Nepal; stigma; psychotherapy; pesticides; middle income countries; randomized controlled trial; primary care; prevention strategy; ethno psychology; gay; post traumatic stress; severity; intimate partner violence; hanging; substance abuse; Pakistan; terrorism; stress; deliberate self harm; suicidal behaviour; injuries; suicide bombing; medical students; abuse; experience; war; poisoning; hopelessness; low income; therapy; management;
C10	Post traumatic stress disorder, veterans, military, deployment, resilience, combat, traumatic brain injury, mental health problems, military personnel, war, soldiers, comorbidity, trauma, us veterans, service, personnel, terrorism, anxiety, intimate partner violence, demography, deaths, stress, injury, rural Bangladesh, assault, pregnancy, verbal autopsy, domestic violence, maternal mortality, marital status, dysthymia, accidents, transport, adolescent, behaviours, childhood, income, loneliness, sexual violence, adjustment, gshs;

Table S14. Expanded Outline of country-specific keywords in clusters of countries from South Asia.

Cluster	Country	iso3c	Frequent country specific keywords	Web of Science records	Female mean SMR	Male mean SMR
	**High SMR countries**					
**C6**	Sri Lanka	LKA	Sri Lanka; yellow oleander; Kataragama; stakeholder analysis; informed policy; low and middle income country; militarization; Tamil eelam; achile mbembe; nayomimunaweera; female militant; public health policy; food production; non fatal self poisoning; suicide like acts; third world; sexual respectability;	190	8.84	29.27
	**Medium SMR countries**					
**C7**	India	IND	Maharashtra; agrarian crisis; biotechnology; farmer suicides; dowry death; bt cotton; iot; marginal farmers; Kerala; Jainism; multinomial logistic model; attempted suicides; burn mortality; goa; credit; southern India;	570	12.74	15.91
**C8**	Nepal	NPL	Edinburgh postnatal depression scale; self report questionnaire 20; thinking healthy program; mother child interaction; feasibility studies; pralidoxime; intensive care management; organophosphorus poisoning; gender and sexual minority; cosinor analysis; daily rhythm; pesticides exposure; student's life satisfaction scale; cultural models; urban men; vital surveillance;	41	2.48	14.76
	Pakistan	PAK	Karachi; hijras; paraphylene diamine; chemical burn; mineral content; caliphate; sexual infidelity; coping strategies; honey; metals; coastal area; atomic absorption spectrophotometer; divorcee individuals; adult males; scalp hair; psychiatric history; psychological effect; burnout syndrome; security risk; Sunni Shia sectarianism;	111	4.62	13.07
	**Low SMR countries**					
**C9**	Maldives	MDV		0	1.01	4.64
**C10**	Afghanistan	AFG	Survey sampling; sample weights; epidemiological research design; design effects; sample bias; survey design efficiency; co morbid chronic pain; iv mental disorders; US army; behavioural activation; symptom clusters; older male veterans; induced traumatic stress; working dogs; structured clinical interview; combat related amputations; abuse screening test; massive casualties; veteran's health; administered pt sd scale;	303	4.06	4.84
	Bangladesh	BGD	Tetanus; micro credit; women with disabilities; female garment workers; factory workers; interstitial cystitis; questionnaire phq 9; psychosocial well being; call descriptions; schadenfreude; intension; postpartum suicidal ideation; in depth network; interval; depression risk factors; Bangladeshi older people; self harming thoughts; reproductive age; diagnostic accuracy;	52	2.41	6.3
	Bhutan	BTN	Parental engagement;	3	2.78	6.07

Latin America and Caribbean region

Table S15. Expanded outline of country-shared keywords in clusters of countries from Latin America and Caribbean.

Cluster	Country shared most common keywords in the cluster
C2	
C4	Relative risks; adolescent and youth suicide mortality rates; teenagers; suicide in young people; average annual percent variation;
C6	
C7	Adolescent; euthanasia; psychopathology; mental disorders; psychiatric disorders; poisoning; suicide prevention; primary health care; comorbidity; common mental disorders; quality of life; childhood; attempted; intentional; self injurious behaviour; sudden unexpected death; suicide in young people; strategy; relative risks; surgery; teenagers; age; period; premature mortality; associations; parental involvement; theory; boys; service attendance; general hospital; parenting; slavery; girls; practice; connectedness; peer; human development; religiousness; peer victimization;
C8	Latin America; teenagers; relative risks; suicide in young people; food insecurity; bipolar disorder; anti retroviral therapy; blood; anthropology; young adults; sex; infection; HIV; psychopathology; victimization; lithium; adherence; life expectancy; firearms; suicidal behaviour; inequalities; schizophrenia; school health; insecticides; sexual behaviour; multilevel; population health; alcohol drinking; serotonin transported gene; determinant; socio economic; tobacco; neurotoxicity; middle school students; applicators; chlorpyrifos; pesticides; self harm; meaning in life; personality traits; altitude; self inflicted injury; risk behaviour survey; agricultural workers; agriculture; hunger; search; patterns; drinking water; fatigue; political violence; sleep deprivation; medical errors; low income countries; sexual behaviours; obesity; family practice; physicians; family; malnutrition; demographic factors; family health; externalizing problems; internalizing problems; community based participatory research; age; pregnancy; gun; homicide rates; laws; urban; anxiety; serotonin re uptake inhibitors; emotional violence; Cambodian refugees; ethno psychology; chronic diseases; violence against children; child maltreatment; paradox; middle income countries;
C9	London, mental health disorders, Trinidad, adolescent, alcohol drinking, bullying, quality of life, english speaking Caribbean, hospital admission rates, substance use, public health surveillance, middle school students, woulds and injuries, school health, traffic accidents, sequelae, sexual behaviour, Latin America, tobacco, accidents, developing countries, system, Mexico, aggression, childhood, firearms, Jamaica, behaviours, suicide attempts, depressive symptoms, abuse, stress, associations, body image, religiousness, psychosocial distress, latino, developing country, England and wells, LGBT, Peru, pregnancy, postpartum women, Spain, suicide attempt, sleep quality, intimate partner violence, spirituality, outcomes, phq 9, cohort, asthma, altitude, disturbances;
C10	Latin America, deaths, mental disorders, comorbidity, depressive symptoms, developing

	countries, bullying, suicide attempt, adolescent, psychiatric disorders, validity, Mexico, aggression, substance use, Nicaragua, firearm availability, developing countries, surveillance, drug use, abuse, sexual abuse, intimate partner violence, domestic violence, suicide in young people, adolescent and youth suicide, average annual percent variation, family, behaviours, meta analysis, associations, connectedness, parental involvement, spirituality, schizophrenia, public policy, sexual behaviour, health policy, relatives, alcohol drinking, organophosphate, shool health, outpatients, middle school students, mental health service, carbamate, Aymara, tobacco, predictors, bipolar disorder, quality of life, validation, follow up, external causes, age, scale, men, mood disorders, pregnancy, self harm, Colombia, euthanasia, hopelessness, life expectancy, homicides, assisted suicide, impulsivity, college students, older adults, self esteem, high school students, dementia, suicide mortality, vital statistics, autonomy, pesticide policy, pesticide poisoning, immigrant, populations, acculturation, forensic science, injury, stressful life events, suicidal expressions, para suicide, young people, content analysis, relative risks, resiliency, instruments, disease, anxiety disorders, global burden, indigenous, suicidal ideation, phq 9, peoples, serious psychological distress, gene therapy, married women, cancer gene therapy, biosynthesis, accumulation, inhibitor, multi country, mental health, common mental disorders, child abuse, brain tumours, peer victimization, peer, girls, parenting, boys, water, resistance, population based, strain, Huntington, s disease, emotion, mutation, violent death rate, internet searching, humanitarian injustice, populism;

Table S16. Expanded Outline of country-specific keywords in clusters of countries from Latin America and Caribbean.

Cluster	Country	iso3c	Frequent country specific keywords	Web of Science records	Female mean SMR	Male mean SMR
	**High SMR countries**					
**C4**	Guyana	GYU	Names; toxic plants; ichthyotoxic plants; one homicide; catharsis; mass suicide;	18	15.43	53.76
**C2**	Suriname	SUR	Global health governance; multi lateral development banks; mercury exposure; Hindustan culture; systems thinking;	4	11.31	37.44
**C6**	Uruguay	URY	Cocaine base post; Uruguay; historical evolution;	10	7.46	27.84
	**Medium SMR countries**					
**C7**	Chile	CHL	Dangerous behaviour; cocaine related disorders; hidden populations; partial remission; low risk; toxic actions; double effect principle; immuno competent infants; computer communication networks; depression postpartum; chronicity; secondary care; letting die; ii depression; privileged access interviewers; rapa nui;	74	3.61	17.23
	Cuba	CUB	Cuba; epidemiology descriptive; refractory temporal lobe epilepsy; deaths on epilepsy; sudep; liberal government; family relations;	23	6.34	21.71
	Trinidad and Tobago	TTO	Trinidad and Tobago; consultation liaison; social predictors;	8	4.73	18.79
**C8**	Argentina	ARG	Maria Ines Krimer; novela negra; boron; thyroid stimulating hormone; iodine; dead; pierce; genero negro; mental health policies; sex workers; aggressors; cyberbullying perpetration; new technology; economic changes; psychological assessment; positive persons; terra del fuego; engagement in	30	3.47	14.59

			care;			
	Belize	BLZ		0	1.69	10.65
	Costa Rica	CRI		6	2.06	11.42
	Ecuador	ECU		12	4.97	13.08
	El Salvador	SLV		8	2.64	11.11
	Guatemala	GTM	Guatemala; school health survey; sexual initiation;	4	3.29	12.92
	Haiti	HTI	Community health workers; rural Haiti; adolescent depression;	4	9	10.65
	St. Lucia	LCA		1	1.69	13.86
	**Low SMR countries**					
	Antigua and Barbuda	ATG		0	0.22	1.14
	Barbados	BRB		1	0.58	1.73
	Grenada	GRD		0	0.59	2.93
	Honduras	HND	Honduras; attempted suicide; Tegucigalpa;	3	0.83	3.9
**C9**	Jamaica	JAM	Sickle cell disease; underachievement; historic preservation; king manor; Jamaica queens; packer syndrome; risk for depression; antenatal care; internalizing distress; parental involvement; gender differentiation; disease knowledge; Jamaican adolescents; seeking behaviour; painful crisis;	21	0.89	3.13
	Peru	PER	Subjective constitution; subjective experience; fatherhood; depressive symptomatology; 2a receptor gene; epds; antepartum depressive symptoms; suicide tendency; Korea international cooperation agency; official development assistance; school health; chronic hypoxia;	21	1.74	4.14
	Bahamas	BHS		1	1.18	5.34
	Bolivia	BOL	Bolivia; la paz;	4	4.4	8.12
	Brazil	BRA	The elderly; psychosocial autopsy; community population; temporal distribution; Portuguese version; time series studies; family health program; mortality trends; spectrum disorder; suicide among elderly; Sao Paulo state; south American Indians; reproducibility of results; age factors; bac; fanniidae diptera; diptera calliphoridae; chrysomya albiceps wiedemann; fanniidae; oil;	300	2.41	8.17
**C10**	Colombia	COL	War crimes; sexual dissidence; persons deprived of the freedom; unconstitutional state of things; rights of the prisoners; pesticides toxicity; confounding factors; publication; gender ideology; peace agreements; social dominance; life tables;changing relation; healthcare disparities; residence characteristics; geographers mapping; emotional regulation; death risk; mono amine oxidase activity; social implications;	54	1.9	6.6
	Dominican Republic	DOM		4	1.83	8.14
	Mexico	MEX	International consortium; c peptide; school population survey; amyloid plaques; apoe4; corpora amylacea; tauopathies; hart crane; addiction diagnosis; sero epidemiology; Durango; measurements; iconography; drd2 gene; alleles; mental deviations; relational violence; social reputation; attitude towards institutional authority; child suicide;	282	1.76	7.71

Nicaragua	NIC	Family of origin; socio demographics conditions; hospital surveillance; seasonal pattern; perceived causes of suicide;	11	2.54	7.32
Panama	PAN	Panama;	1	1.28	7.5
Paraguay	PRY	Scopadulcic acid b; traditional herbal medicines of guanary indio; bone resorption; leaf organo culture; bio active di terpene; scop aria dulcis I.; Paraguay; antiviral activity; 2 chemo types; gastric h+,k+ atpase;	2	2.86	6.11
St. Vincent and the Grenadines	VCT		1	1.09	7.32
Venezuela, RB	VEN	Antibiotics; aero monas spp.; Antonio Leocadio Guzman; partido liberal; mangrove oyster; mesophilic aero monads; vibrio;	12	1.11	5.93

North America region

Table S17. Expanded outline of country-shared keywords in clusters of countries from North America.

**Cluster**	**Country shared most common keywords in the cluster**
C7	Euthanasia; suicide attempts; adolescent; mental disorders; substance use; comorbidity; life; Canada; assisted suicide; Inuit; palliative care; medical assistance in dying; anxiety; community; aboriginal; self harm; abuse; social support; England; firearms; psychiatric disorders; major depression; age; adolescent suicide; deaths; behaviour; follow up; stress; patterns; post traumatic stress disorder; physician assisted suicide; primary care;

Table S18. Expanded Outline of country-specific keywords in clusters of countries from North America.

**Cluster**	**Country**	**iso3c**	**Frequent country specific keywords**	**Web of Science records**	**Female mean SMR**	**Male mean SMR**
	**Medium SMR**					
**C7**	Canada	CAN	Nunavut; age standardized mortality rates; metis; home care; cag repeats; Alberta; marijuana use evidence; British Columbia; narrative research; body donation; aboriginal young people; Edmonton; correctional service of Canada; interpersonal trauma; minimum dataset; on reserve; Canadian community health survey; family health promotion; hospital pharmacists; dutch program;	730	6.17	17.98
	United States	USA	Non Hispanic whites; united states veterans; reporting system; purchase; mass shootings; iv audadis iv; youth violence; survivor-ship; national violent death reporting system; precipitating circumstances; general population sample; dsm iv disorders; multiple births; households; rampage shootings; decedents; nvdrs; elevation;	3563	5.91	20.72
