# Supplementary material for: Global Suicide Mortality Rates (2000–2019): Clustering, Themes, and Causes Analyzed through Machine Learning and Bibliographic Data
Source: Int J Environ Res Public Health. 2024 Sep 10;21(9):1202. doi: 10.3390/ijerph21091202 (PMC11431541; doi:10.3390/ijerph21091202)
Supplement: Supplementary file 1 [file ijerph-21-01202-s001.zip › ijerph-3110525-supplementary/SUPPLEMENTARY/Supplementary_Slides_S1_Additional_explanatory_plots_and_graphs.pptx]

## Slide 1
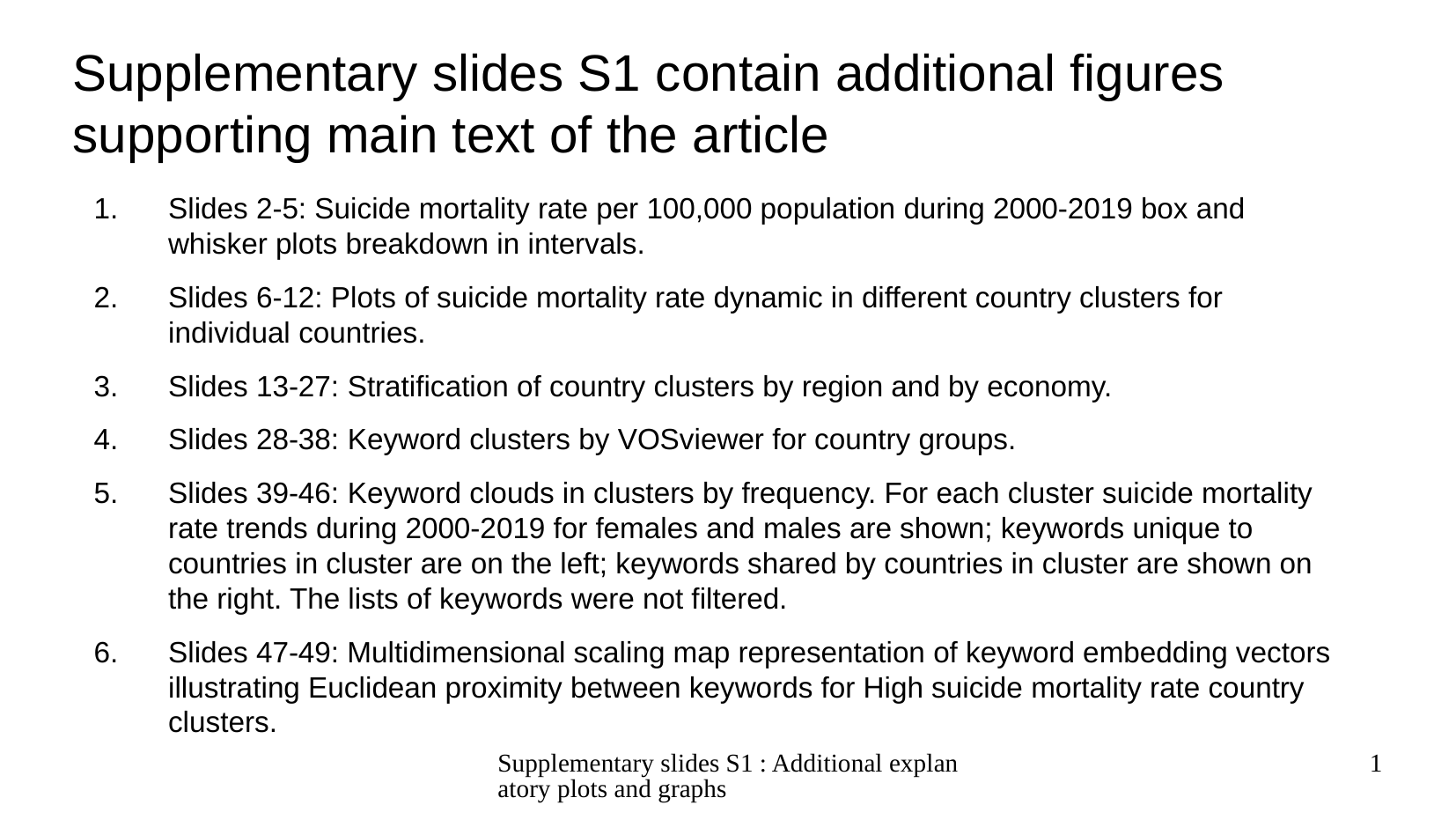

# Supplementary slides S1 contain additional figures supporting main text of the article
Slides 2-5: Suicide mortality rate per 100,000 population during 2000-2019 box and whisker plots breakdown in intervals.
Slides 6-12: Plots of suicide mortality rate dynamic in different country clusters for individual countries.
Slides 13-27: Stratification of country clusters by region and by economy.
Slides 28-38: Keyword clusters by VOSviewer for country groups.
Slides 39-46: Keyword clouds in clusters by frequency. For each cluster suicide mortality rate trends during 2000-2019 for females and males are shown; keywords unique to countries in cluster are on the left; keywords shared by countries in cluster are shown on the right. The lists of keywords were not filtered.
Slides 47-49: Multidimensional scaling map representation of keyword embedding vectors illustrating Euclidean proximity between keywords for High suicide mortality rate country clusters.
Supplementary slides S1 : Additional explanatory plots and graphs
1

## Slide 2
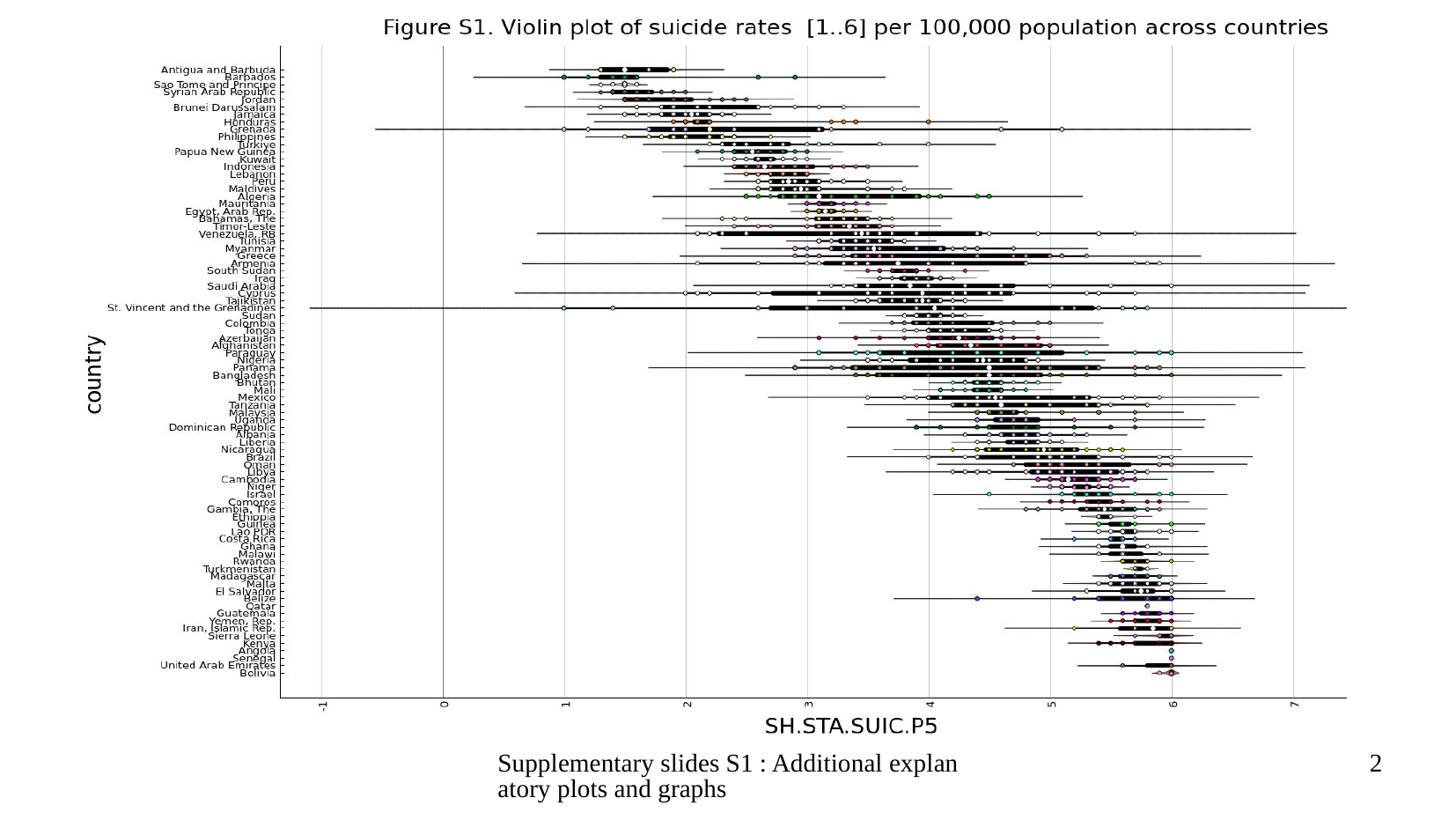

Supplementary slides S1 : Additional explanatory plots and graphs
2

## Slide 3
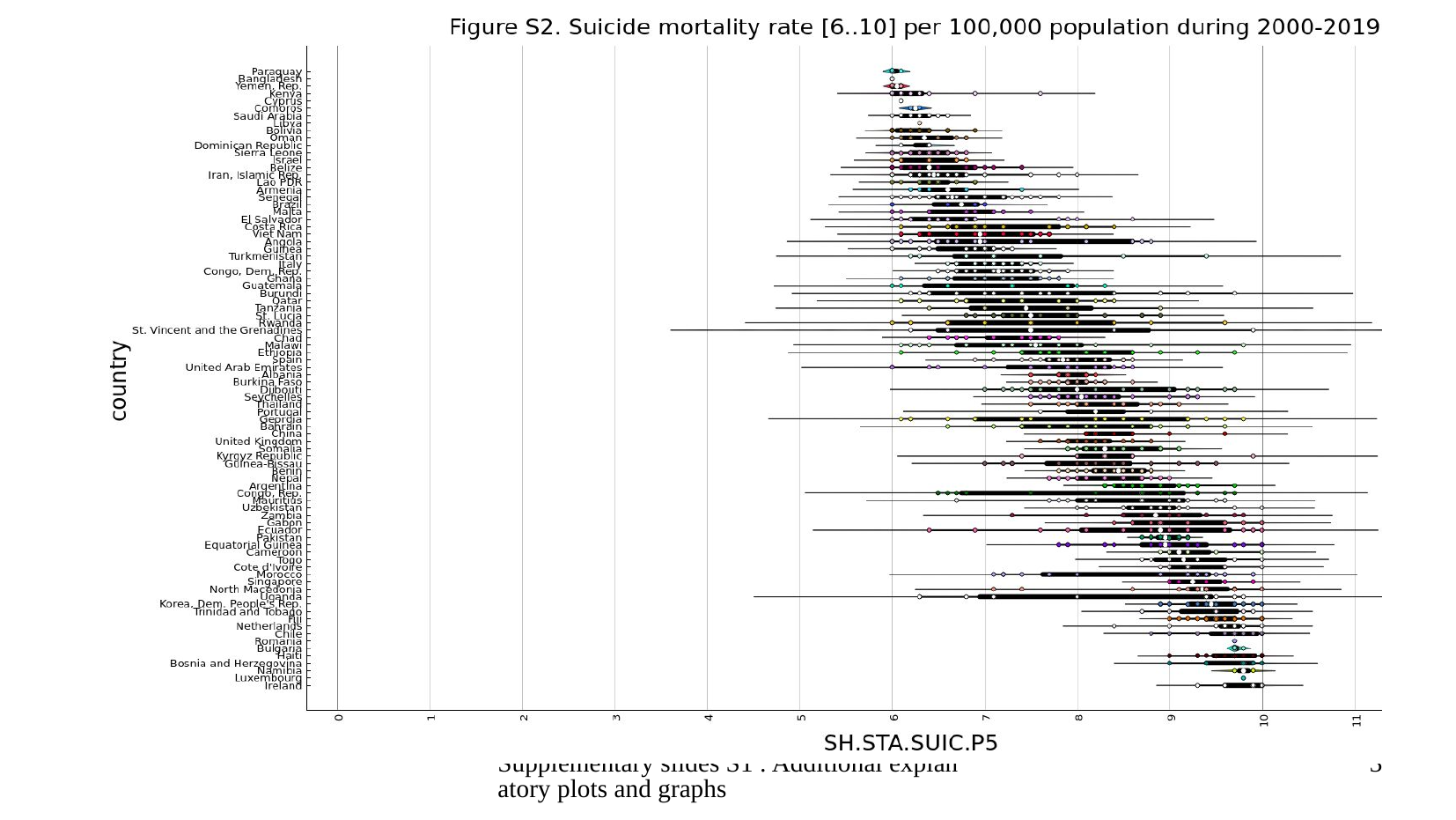

Supplementary slides S1 : Additional explanatory plots and graphs
3

## Slide 4
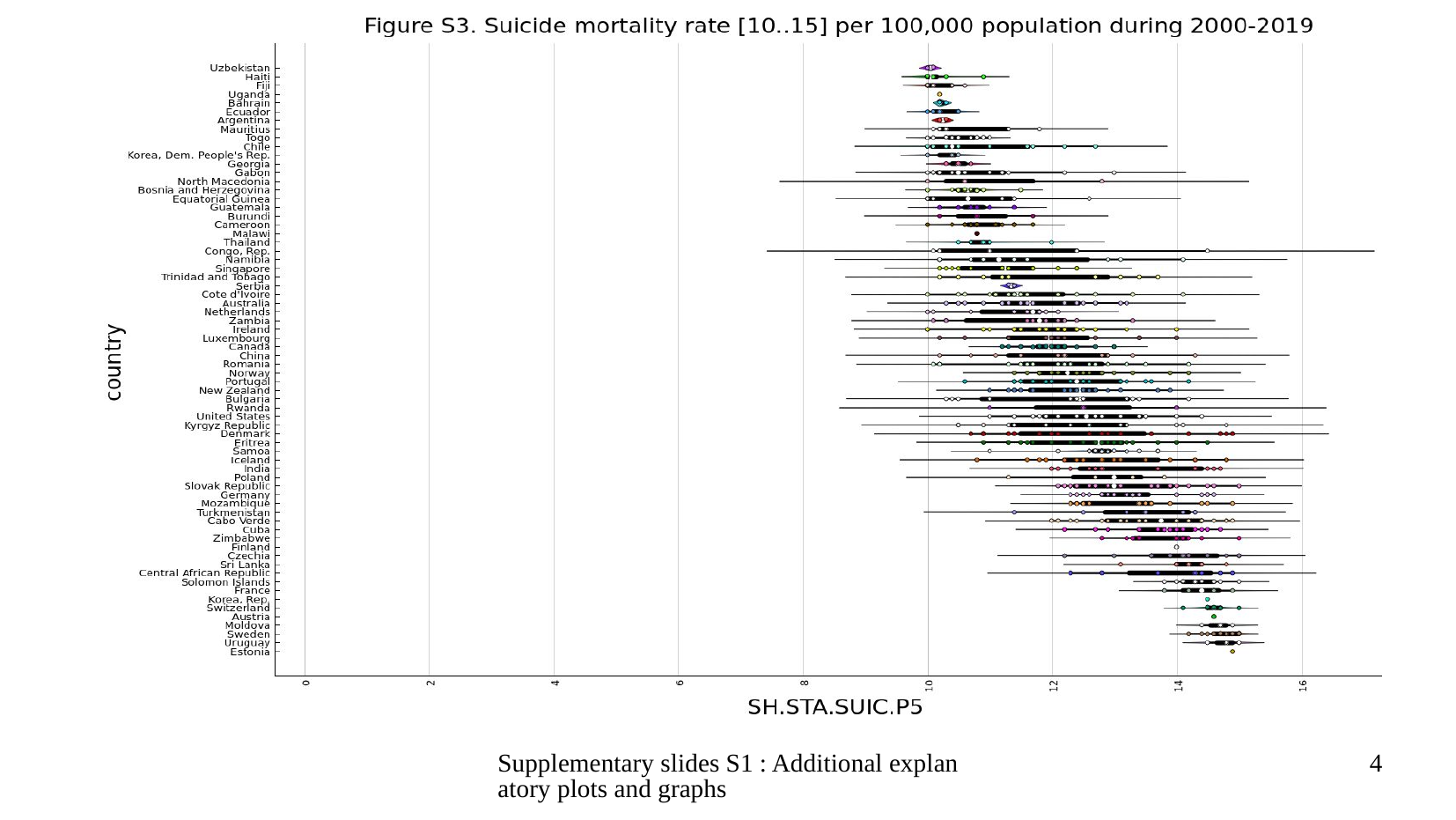

Supplementary slides S1 : Additional explanatory plots and graphs
4

## Slide 5
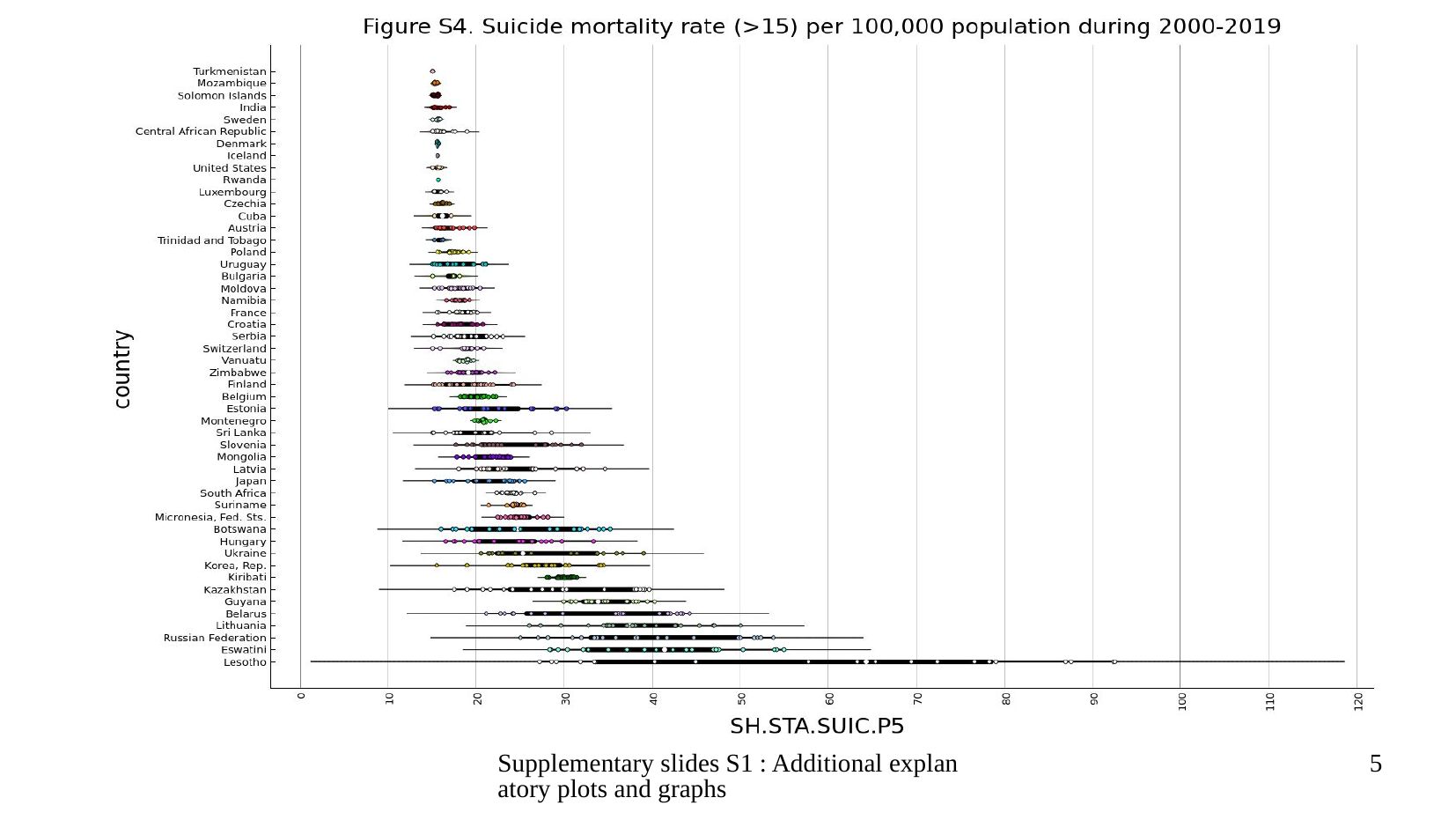

Supplementary slides S1 : Additional explanatory plots and graphs
5

## Slide 6
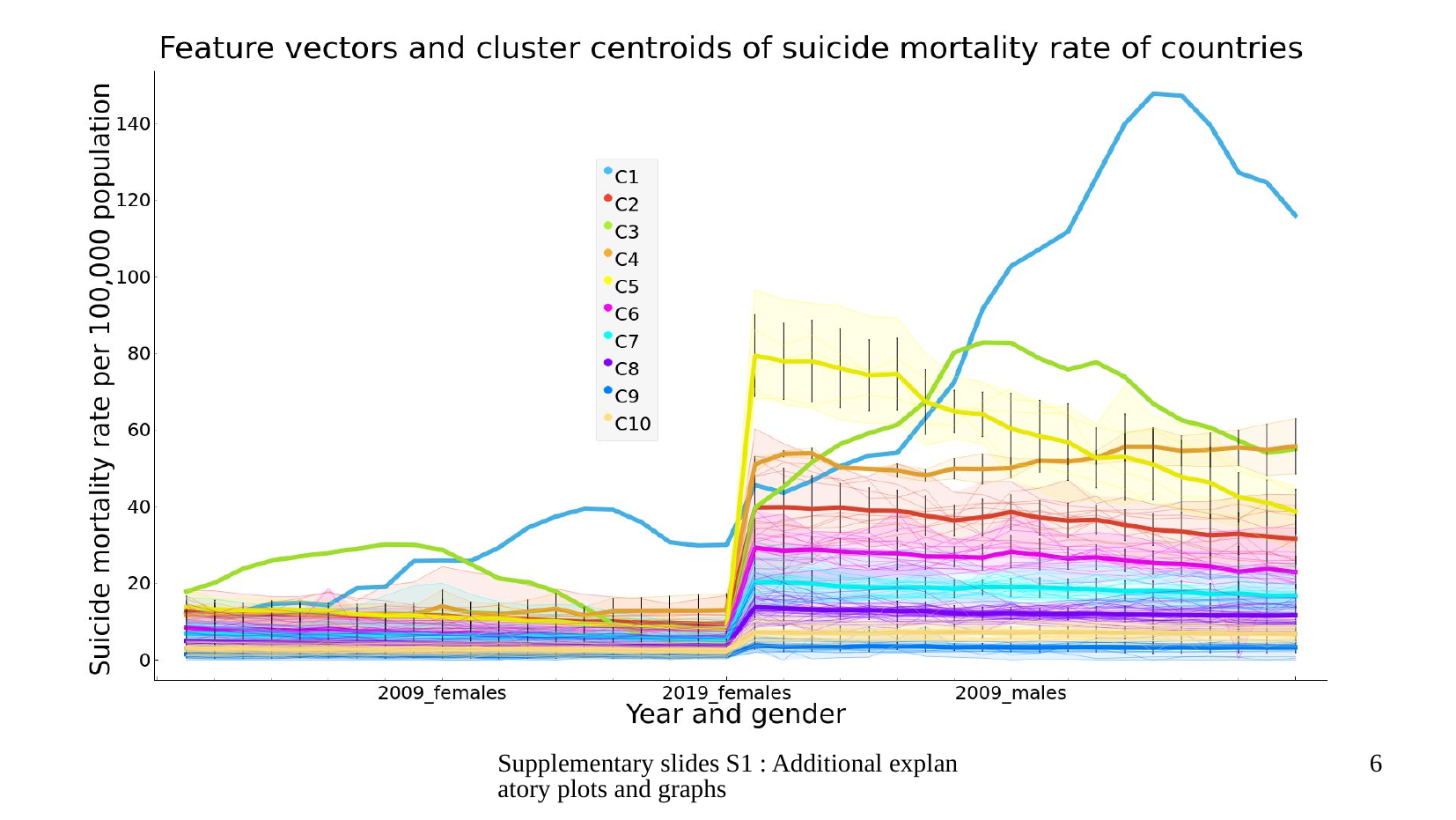

Supplementary slides S1 : Additional explanatory plots and graphs
6

## Slide 7
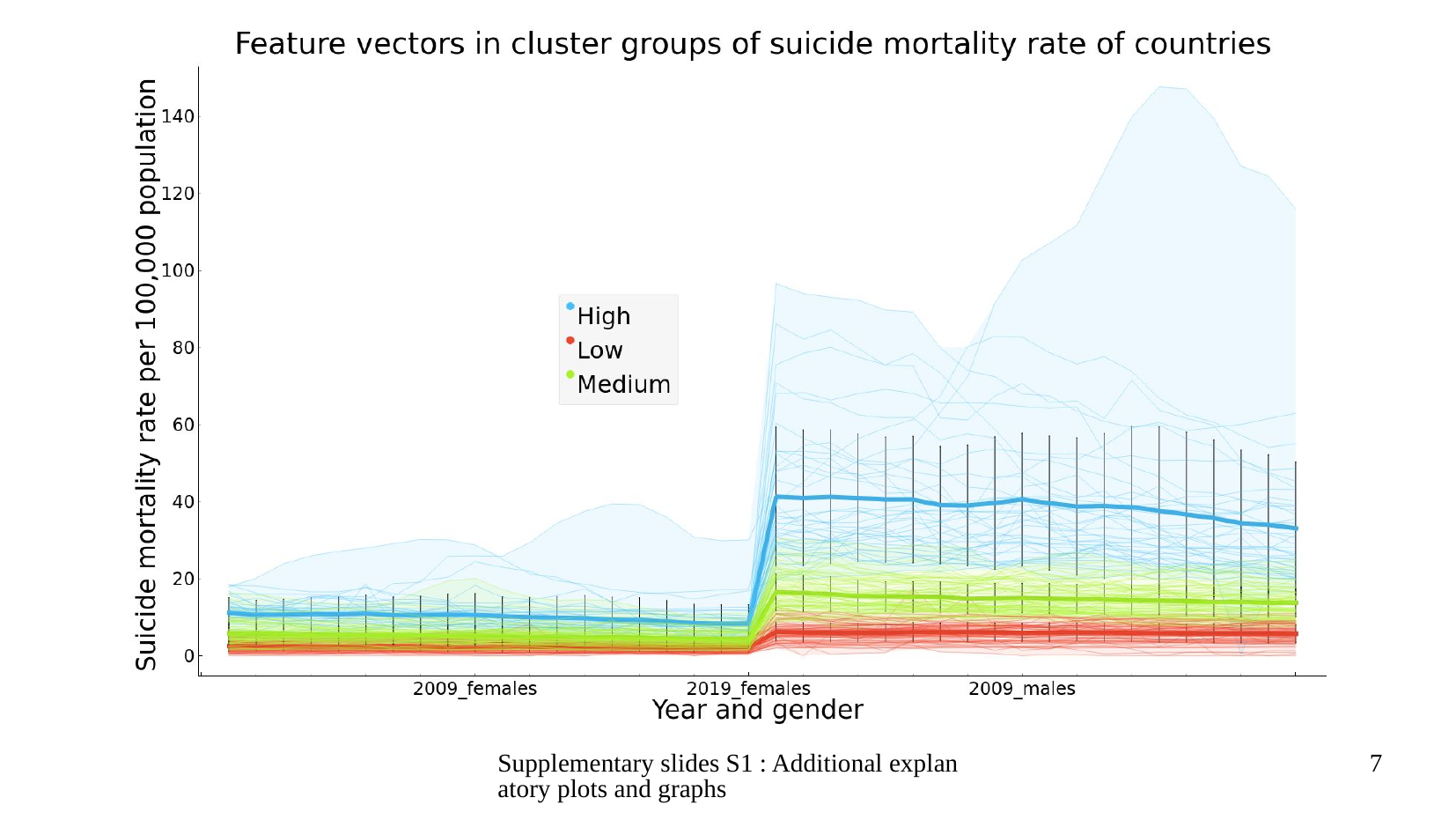

Supplementary slides S1 : Additional explanatory plots and graphs
7

## Slide 8
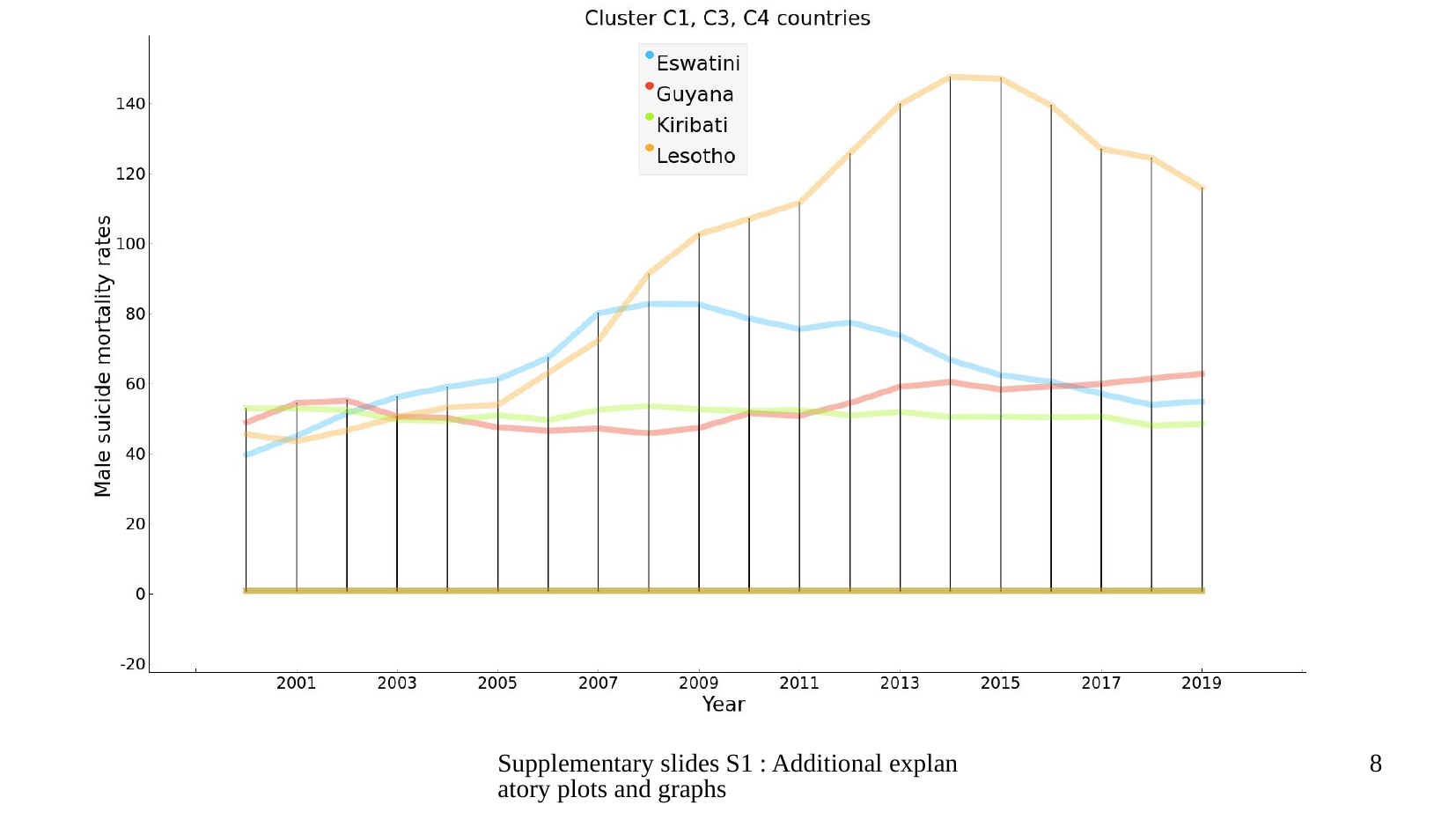

Supplementary slides S1 : Additional explanatory plots and graphs
8

## Slide 9
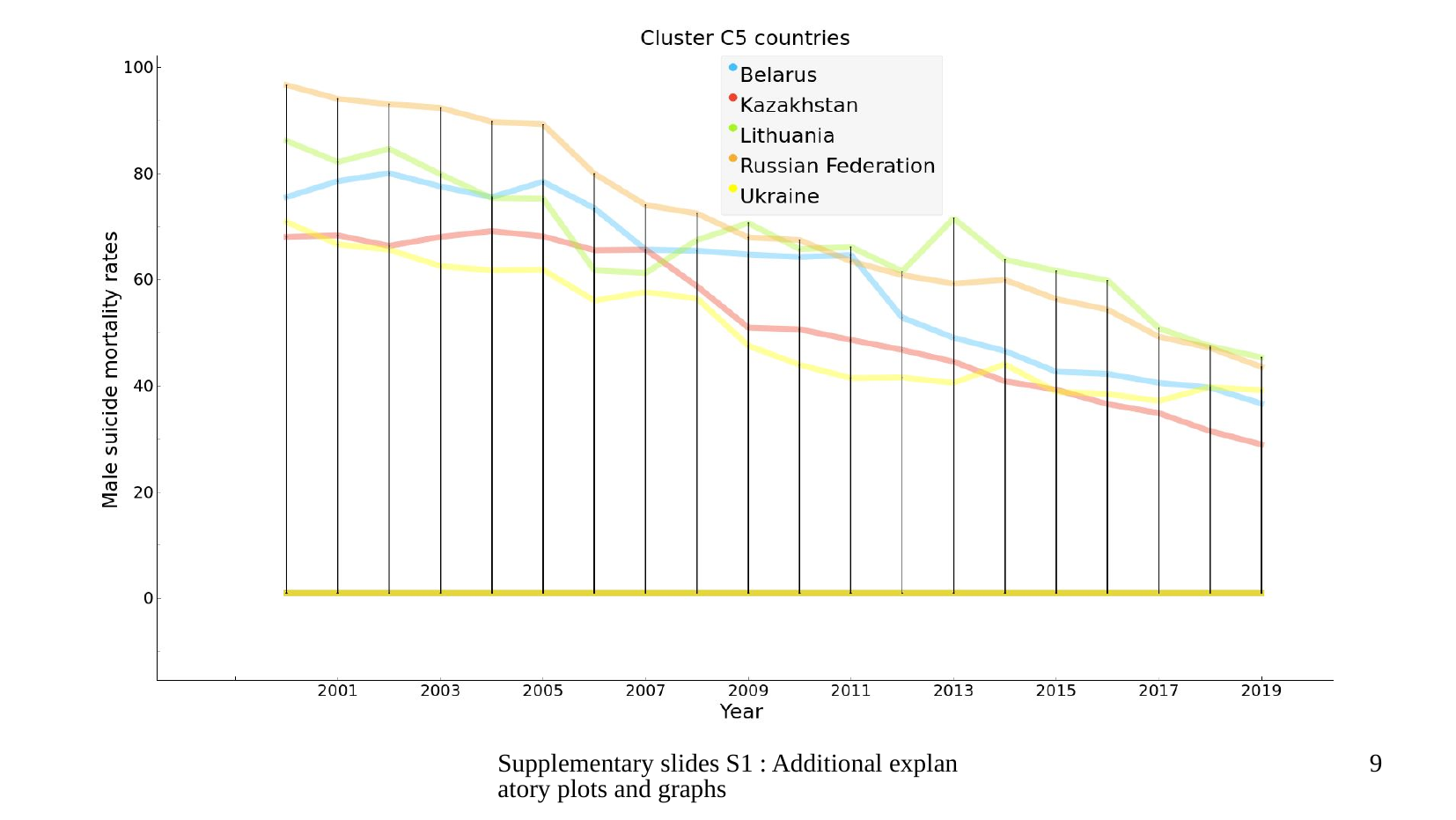

Supplementary slides S1 : Additional explanatory plots and graphs
9

## Slide 10
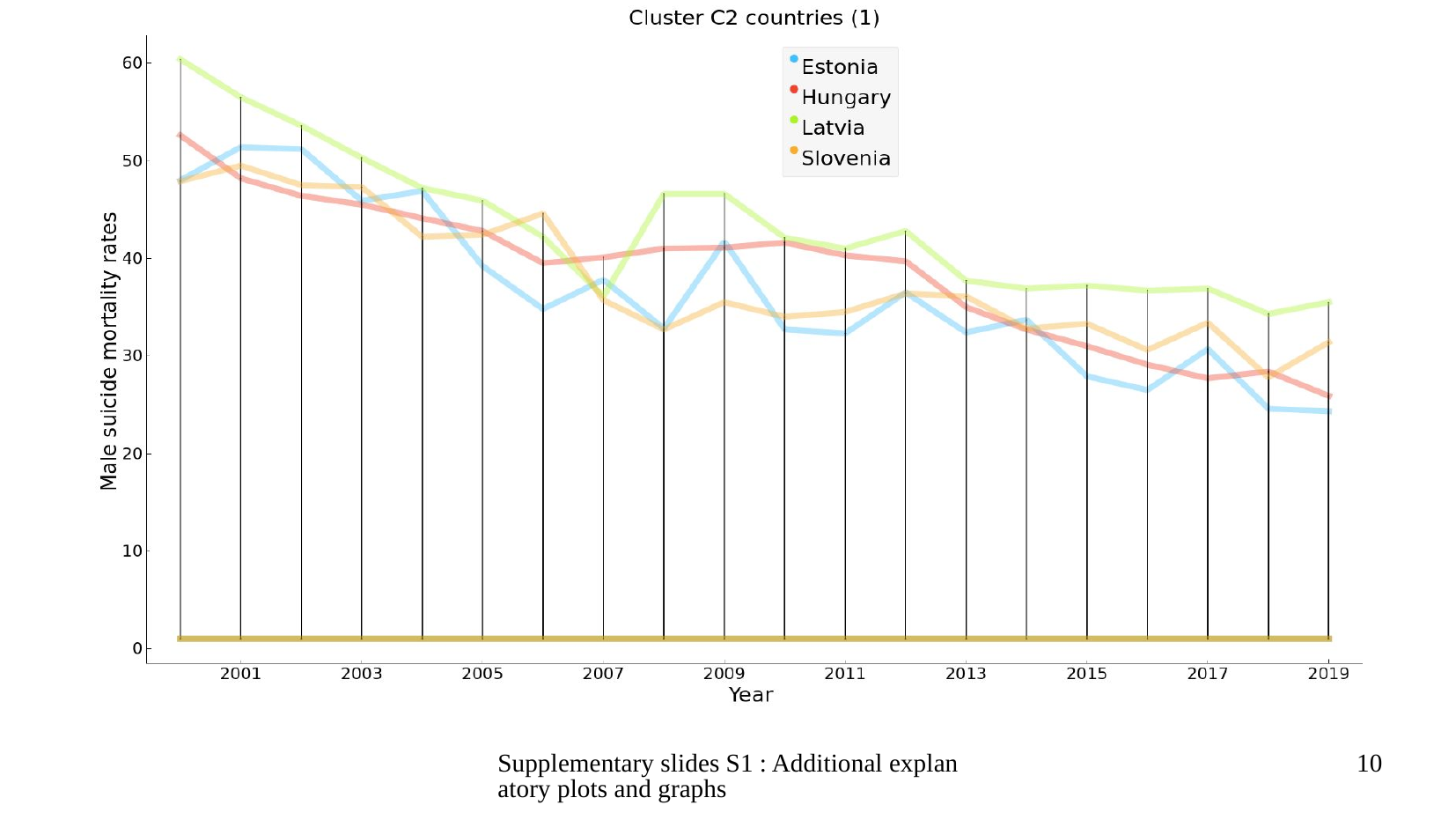

Supplementary slides S1 : Additional explanatory plots and graphs
10

## Slide 11
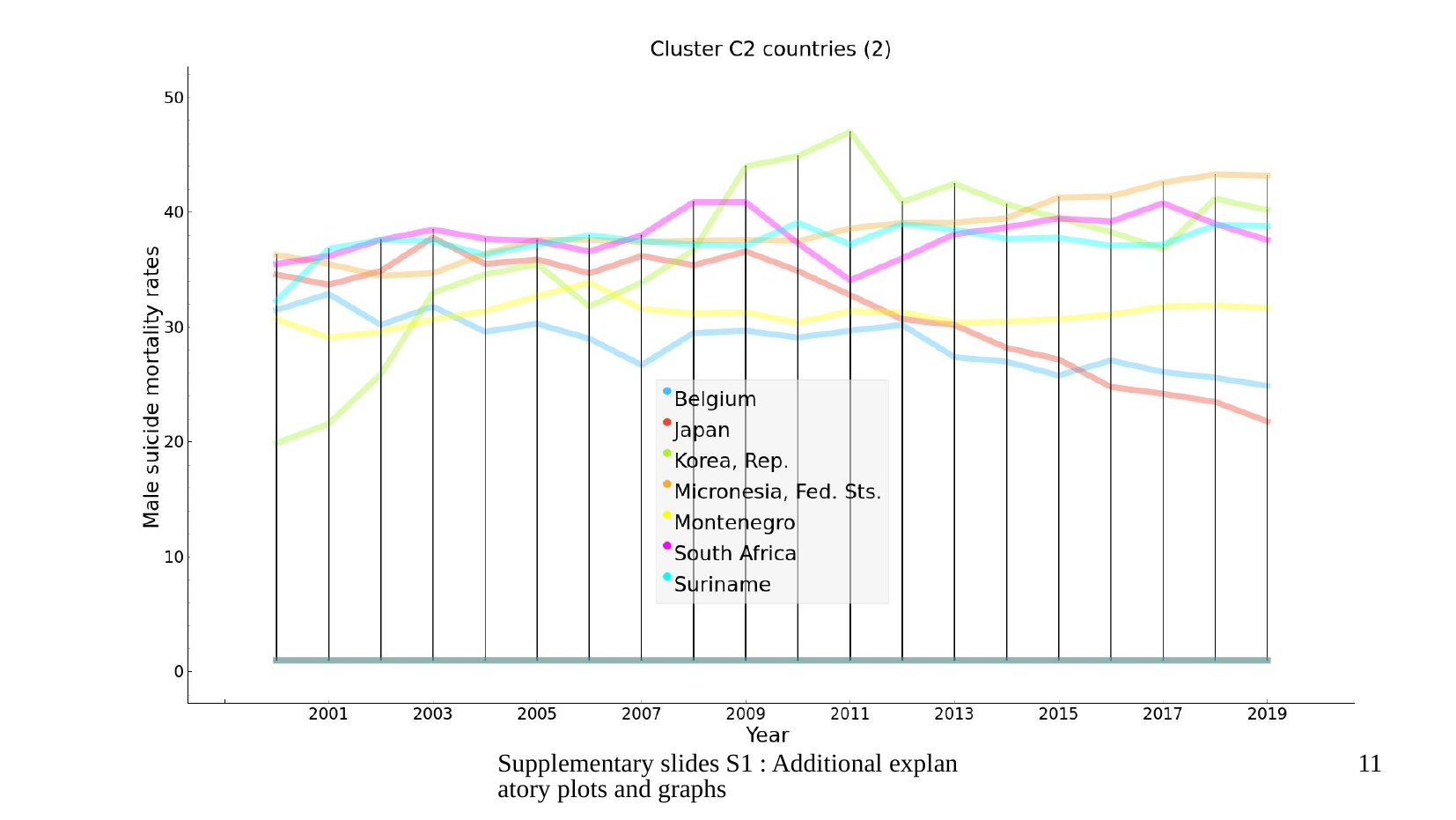

Supplementary slides S1 : Additional explanatory plots and graphs
11

## Slide 12
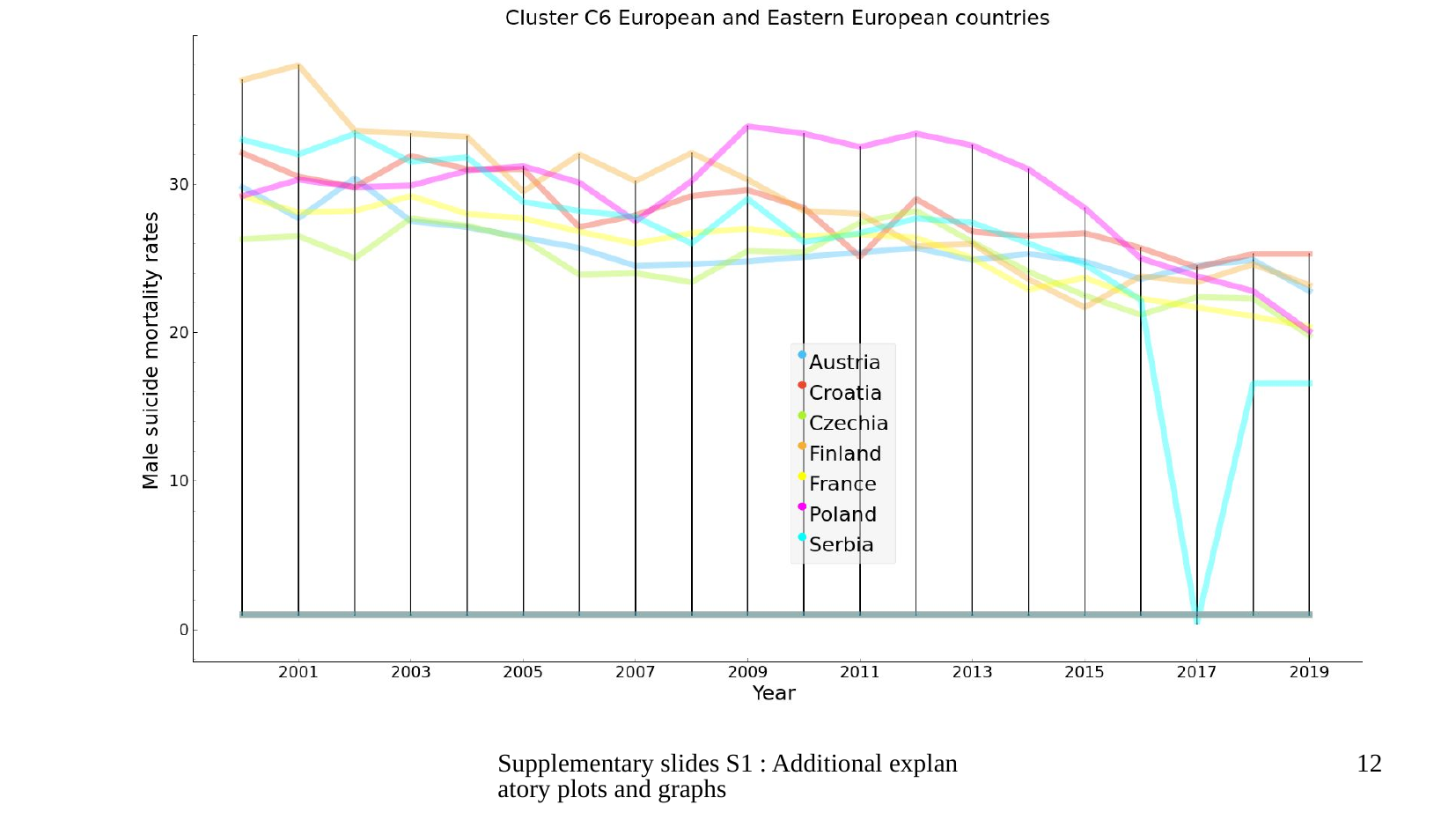

Supplementary slides S1 : Additional explanatory plots and graphs
12

## Slide 13
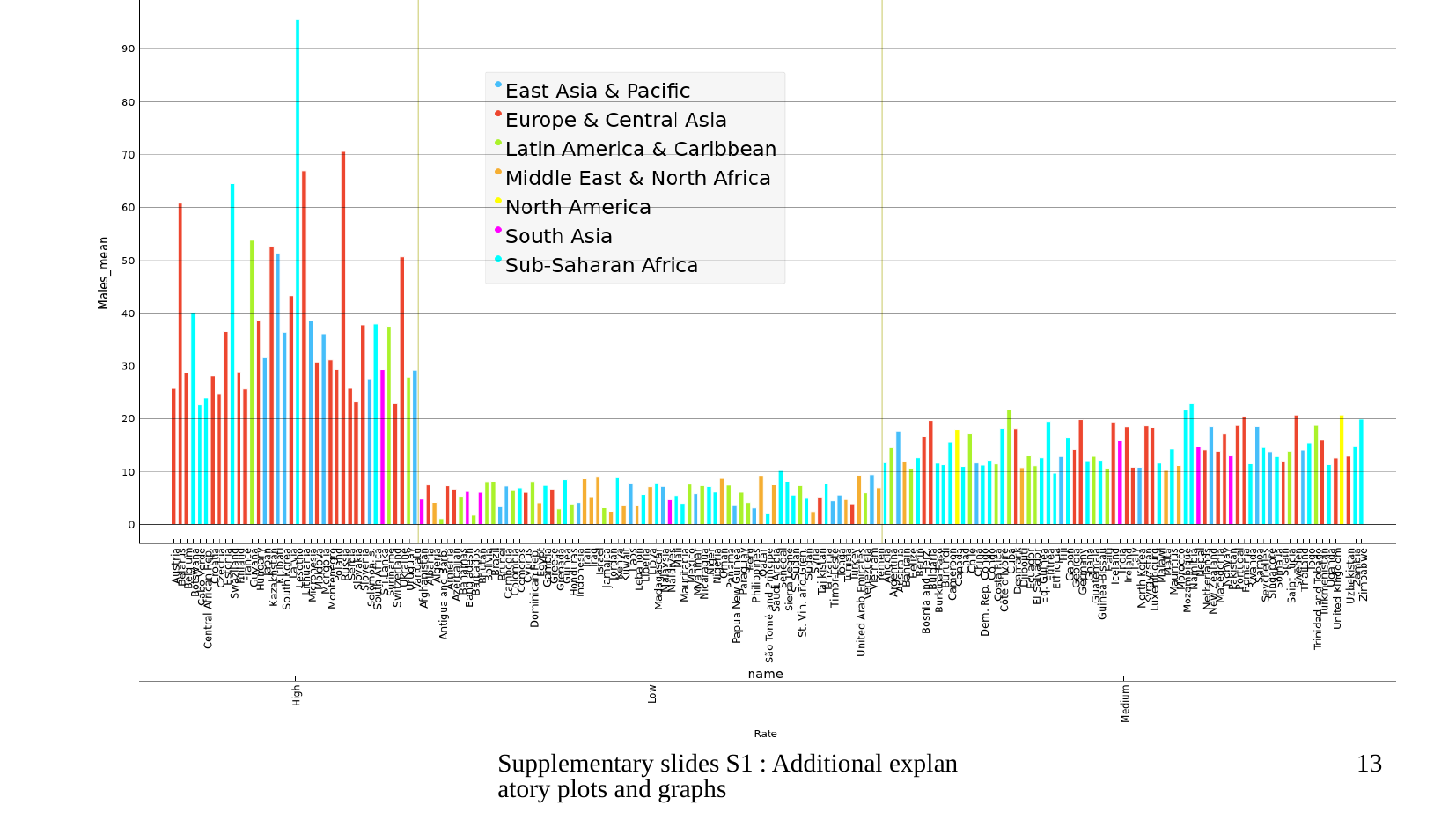

Supplementary slides S1 : Additional explanatory plots and graphs
13

## Slide 14
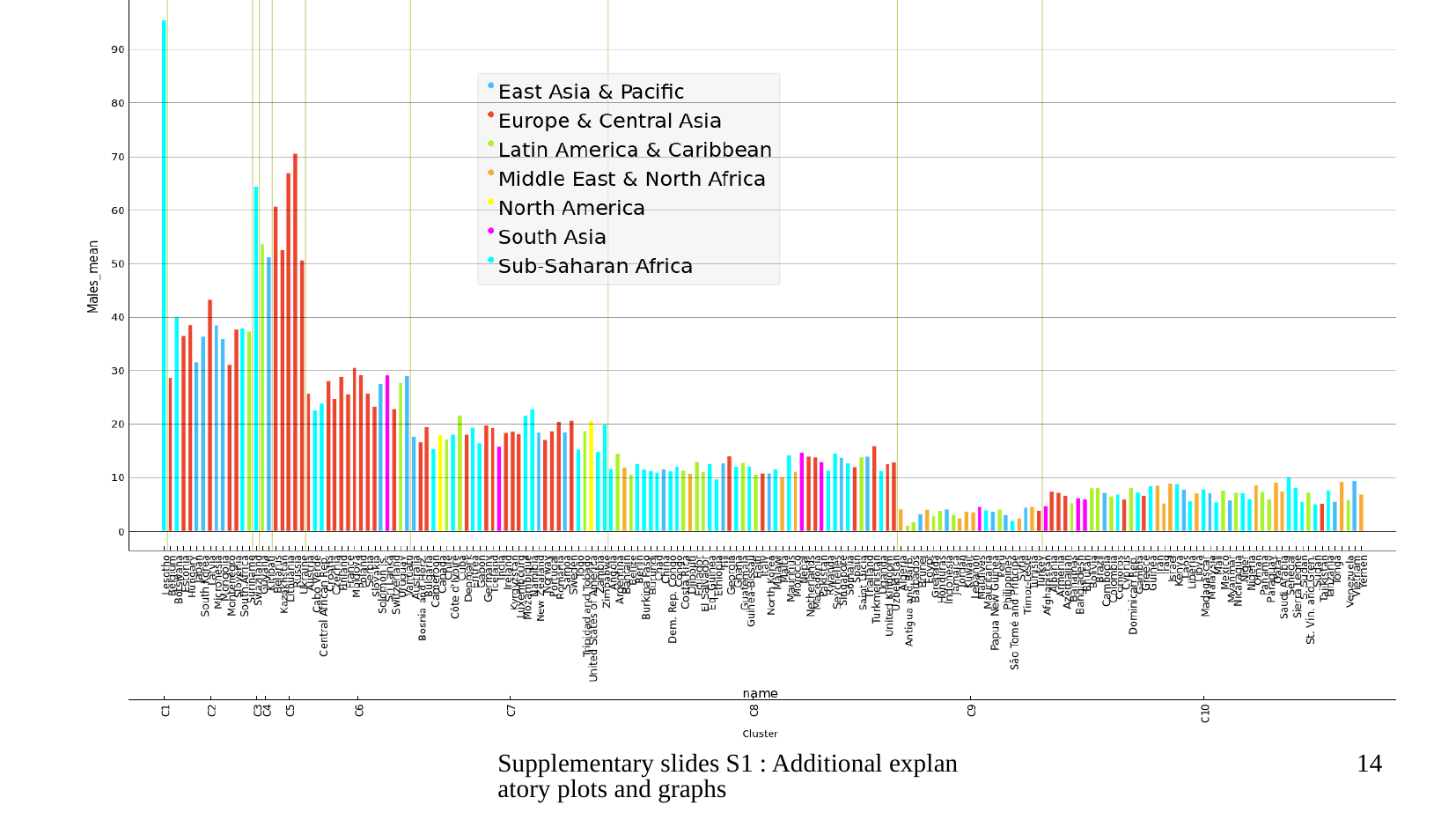

Supplementary slides S1 : Additional explanatory plots and graphs
14

## Slide 15
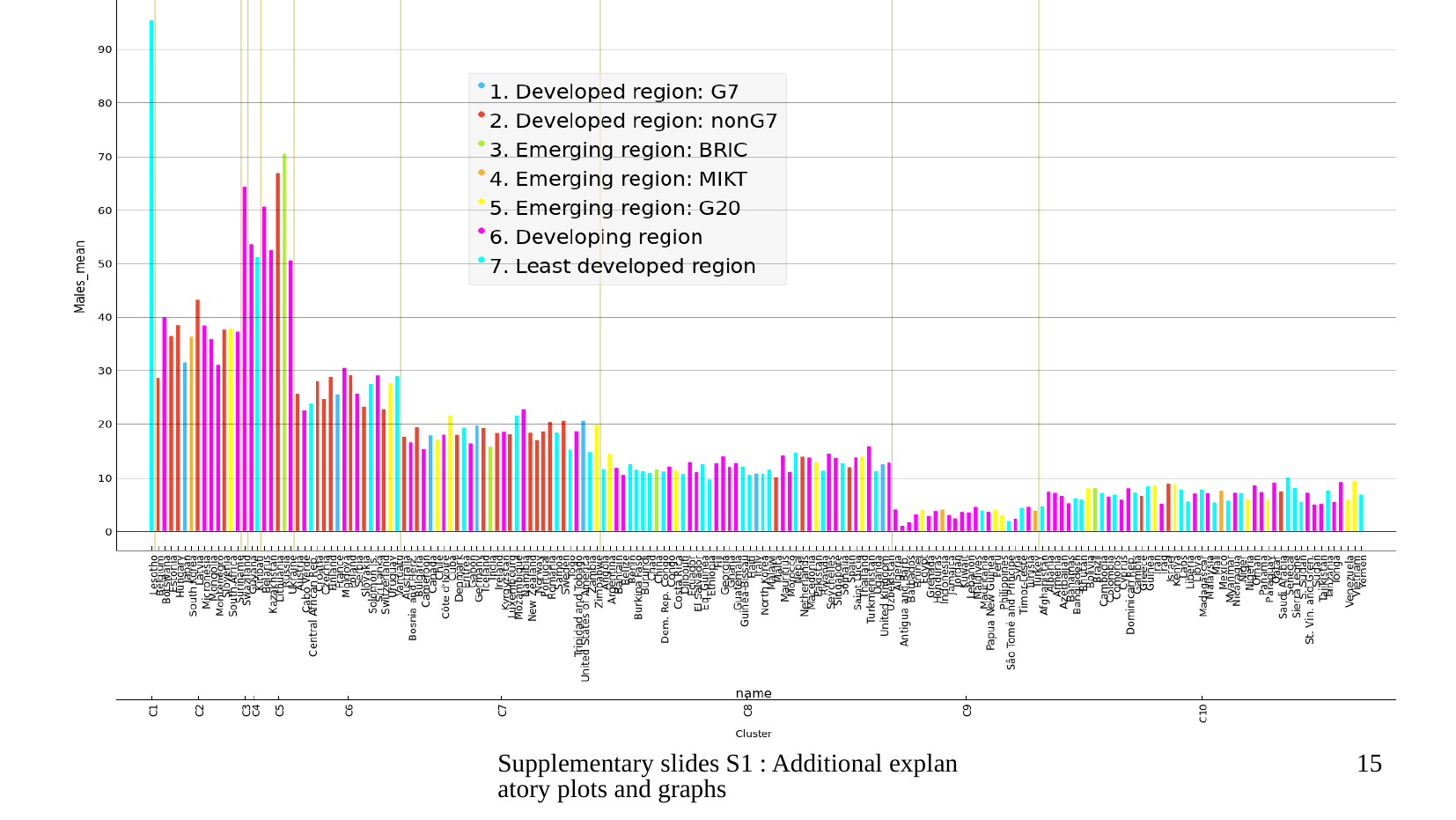

Supplementary slides S1 : Additional explanatory plots and graphs
15

## Slide 16
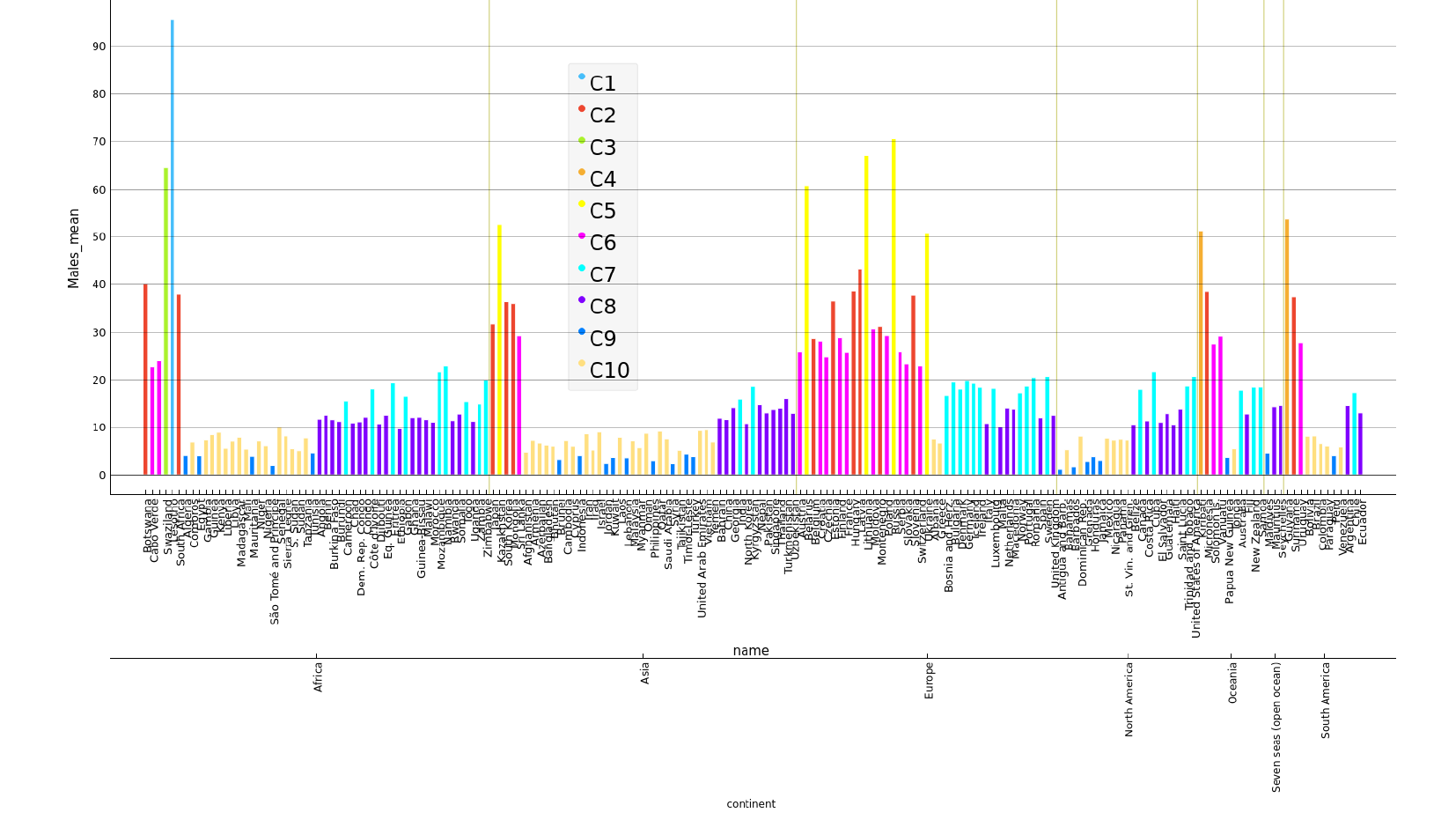

Supplementary slides S1 : Additional explanatory plots and graphs
16

## Slide 17
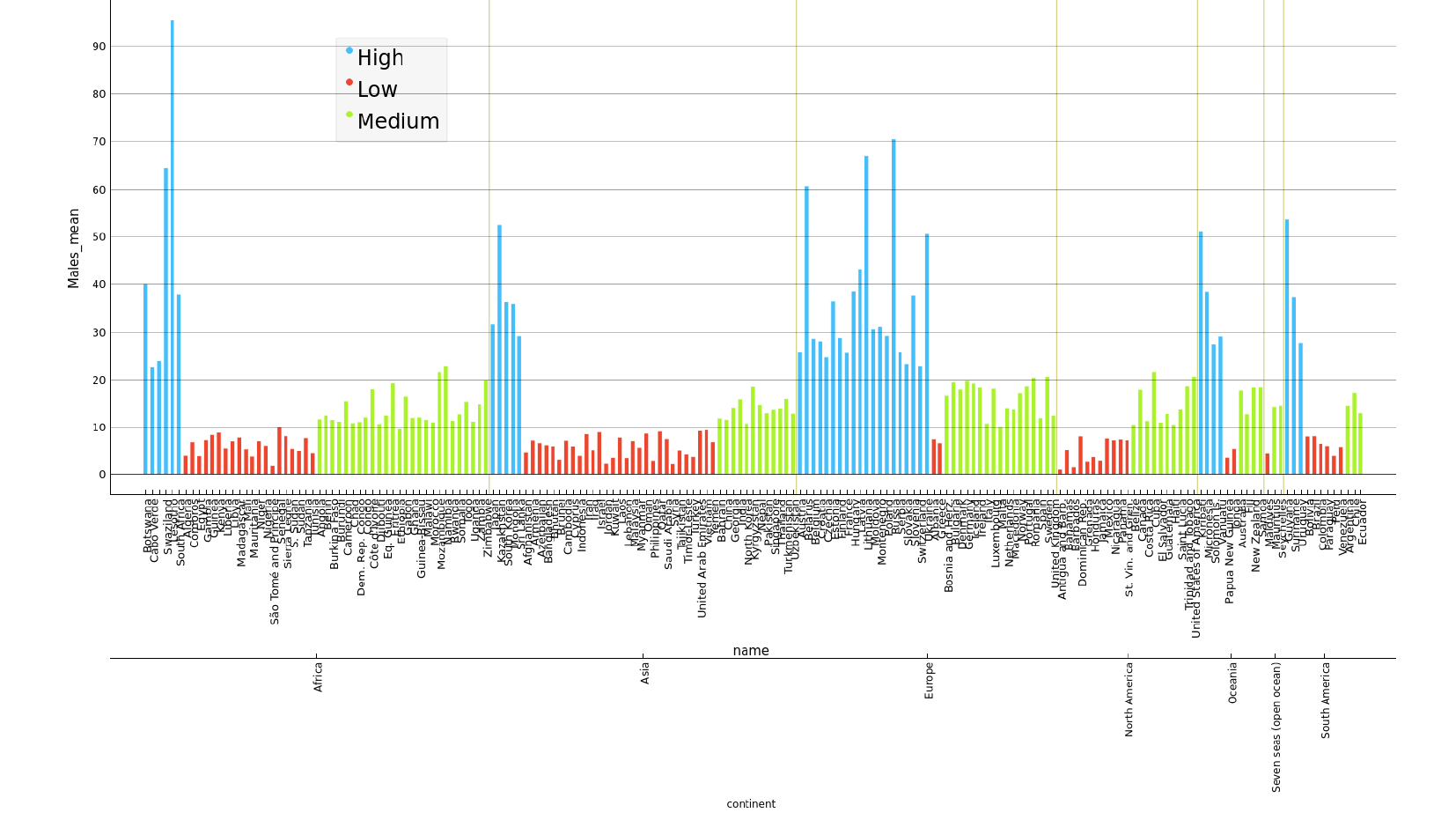

Supplementary slides S1 : Additional explanatory plots and graphs
17

## Slide 18
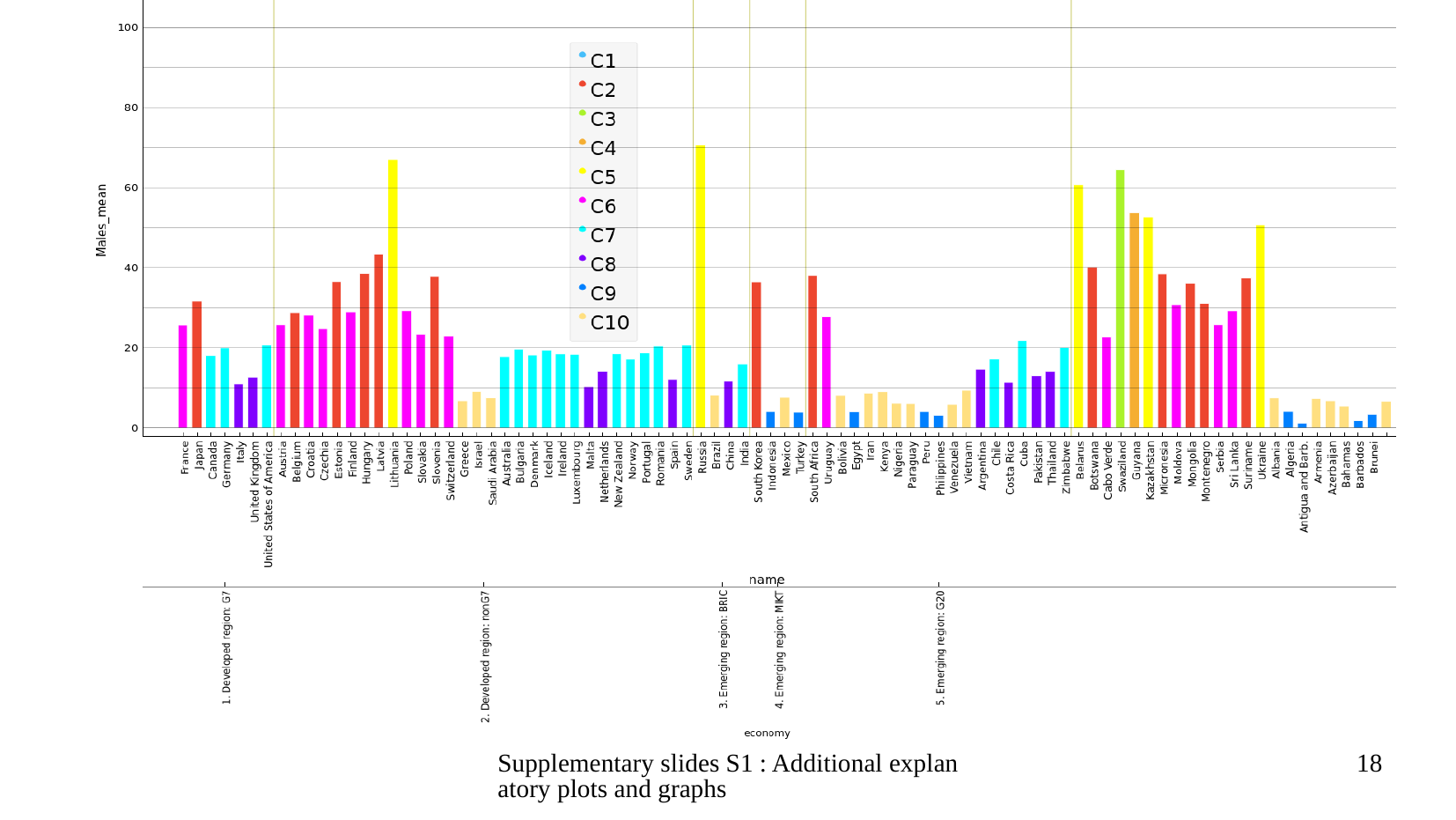

Supplementary slides S1 : Additional explanatory plots and graphs
18

## Slide 19
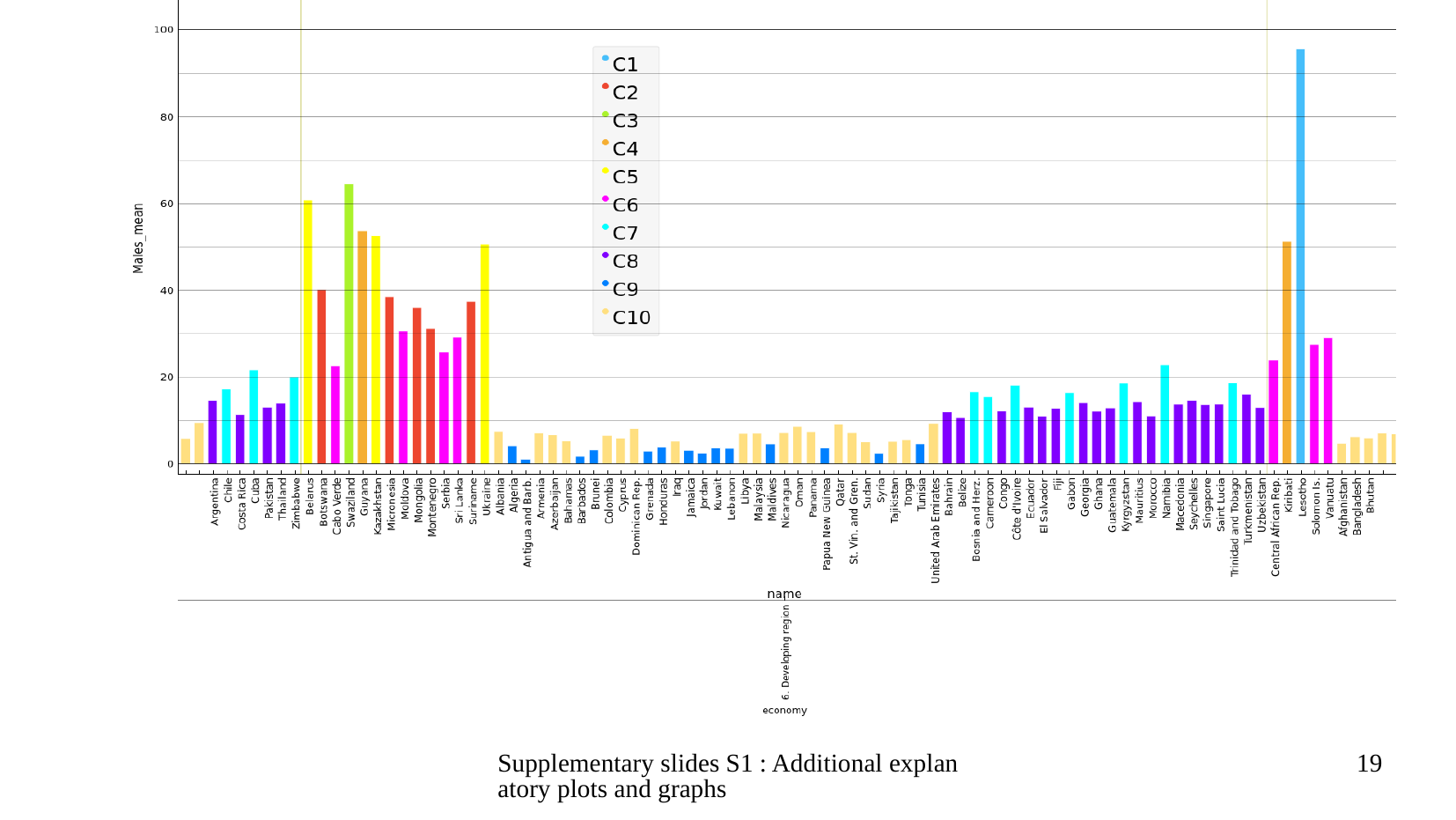

Supplementary slides S1 : Additional explanatory plots and graphs
19

## Slide 20
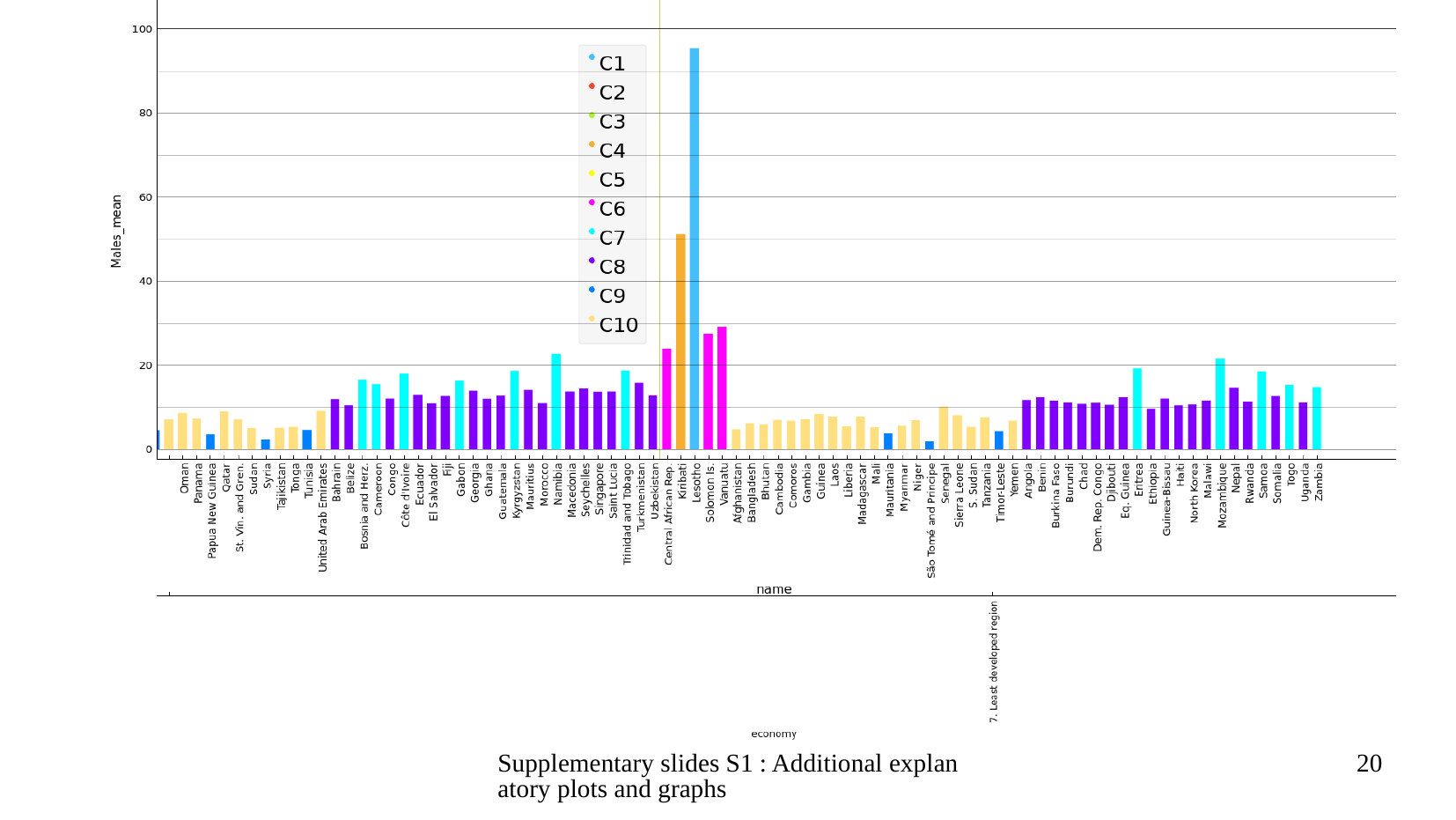

Supplementary slides S1 : Additional explanatory plots and graphs
20

## Slide 21
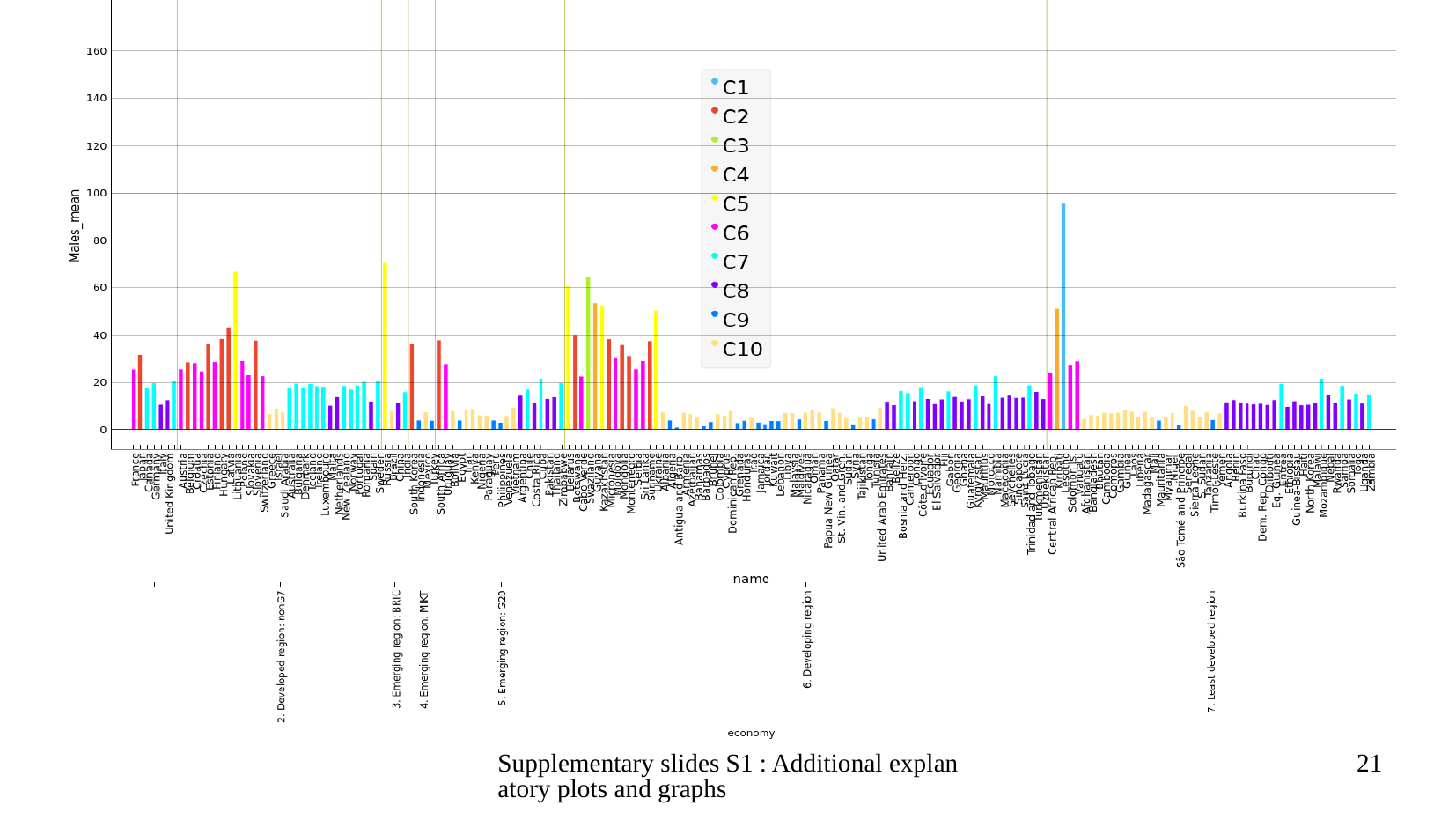

Supplementary slides S1 : Additional explanatory plots and graphs
21

## Slide 22
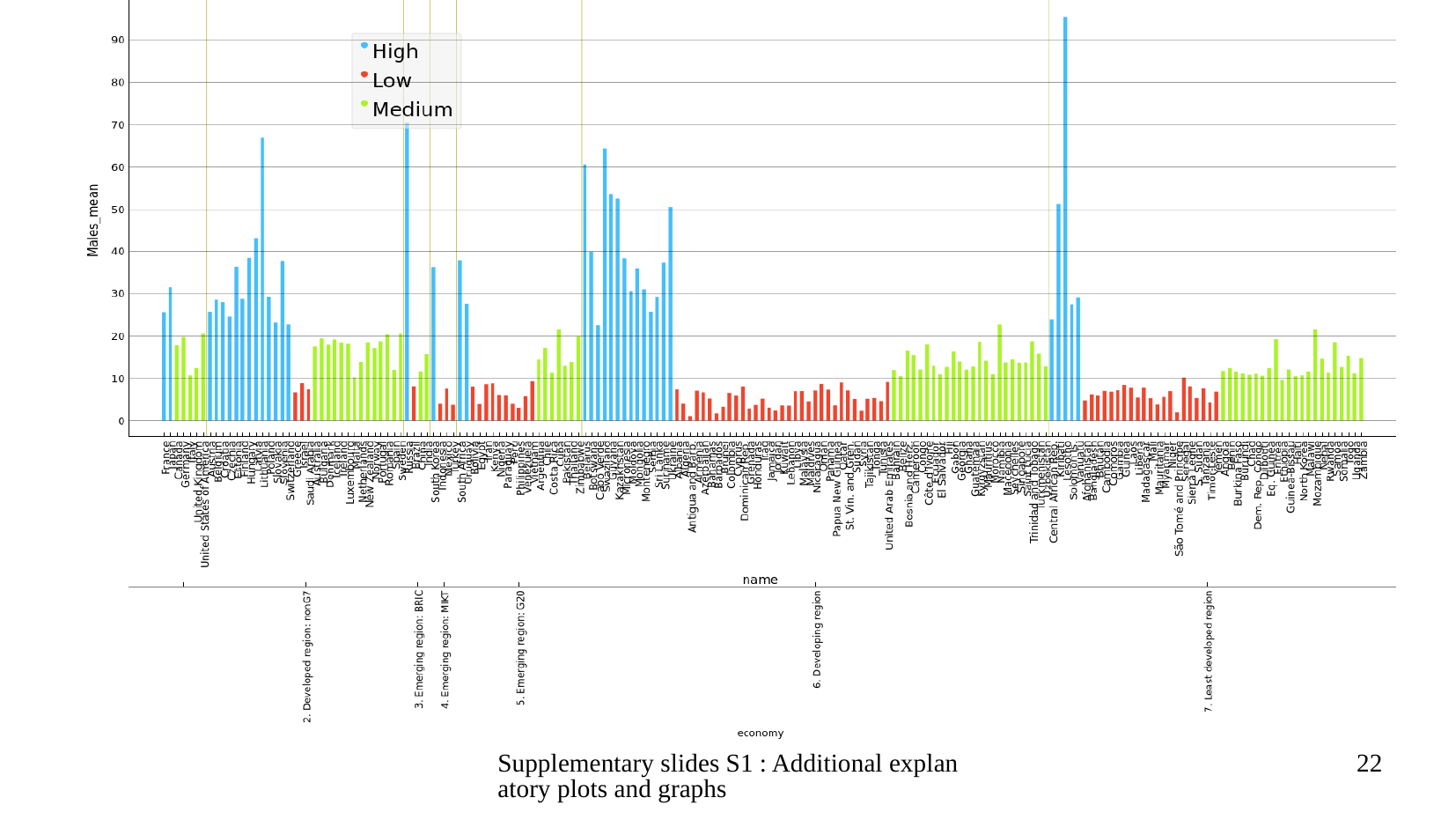

Supplementary slides S1 : Additional explanatory plots and graphs
22

## Slide 23
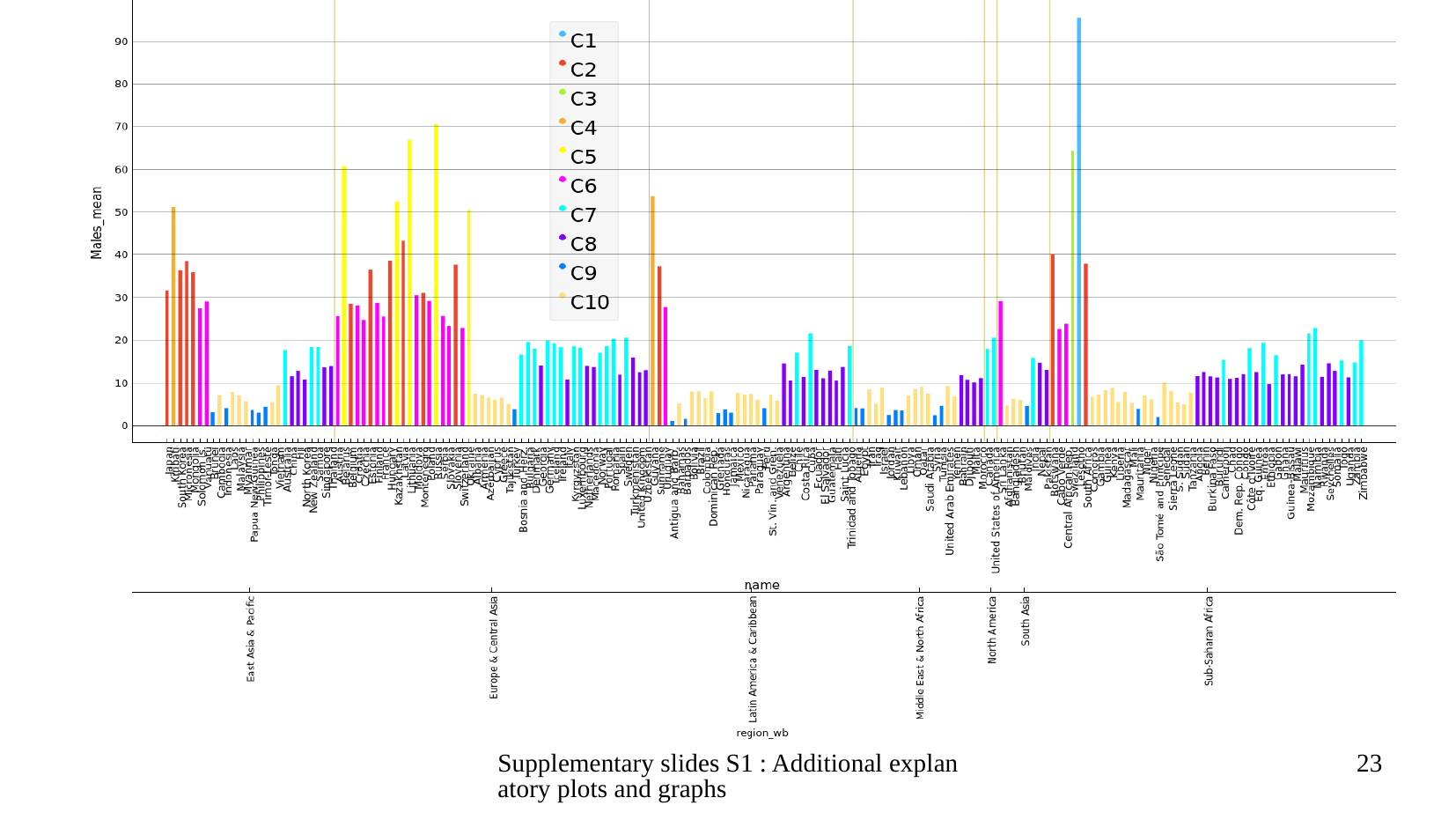

Supplementary slides S1 : Additional explanatory plots and graphs
23

## Slide 24
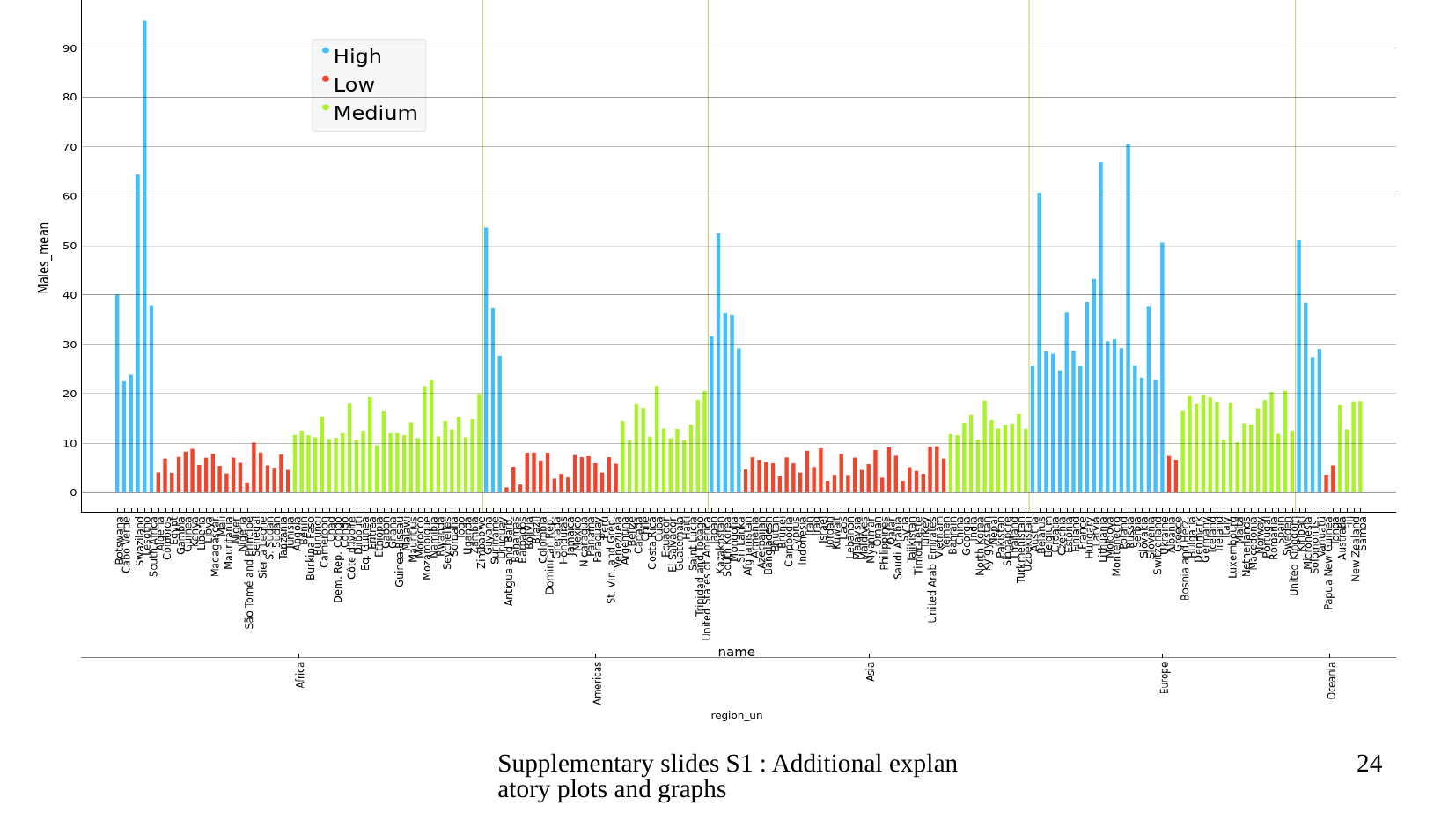

Supplementary slides S1 : Additional explanatory plots and graphs
24

## Slide 25
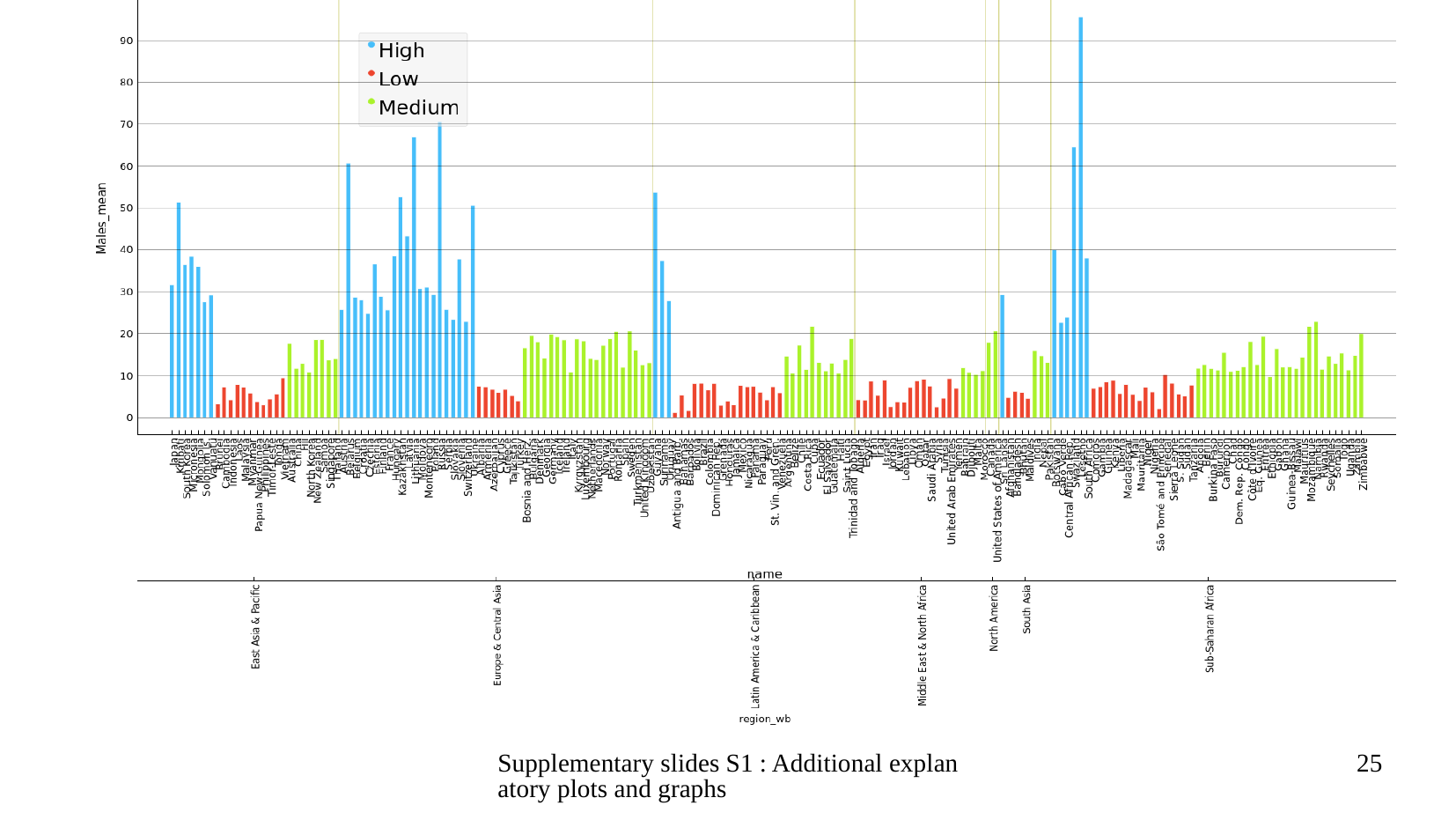

Supplementary slides S1 : Additional explanatory plots and graphs
25

## Slide 26
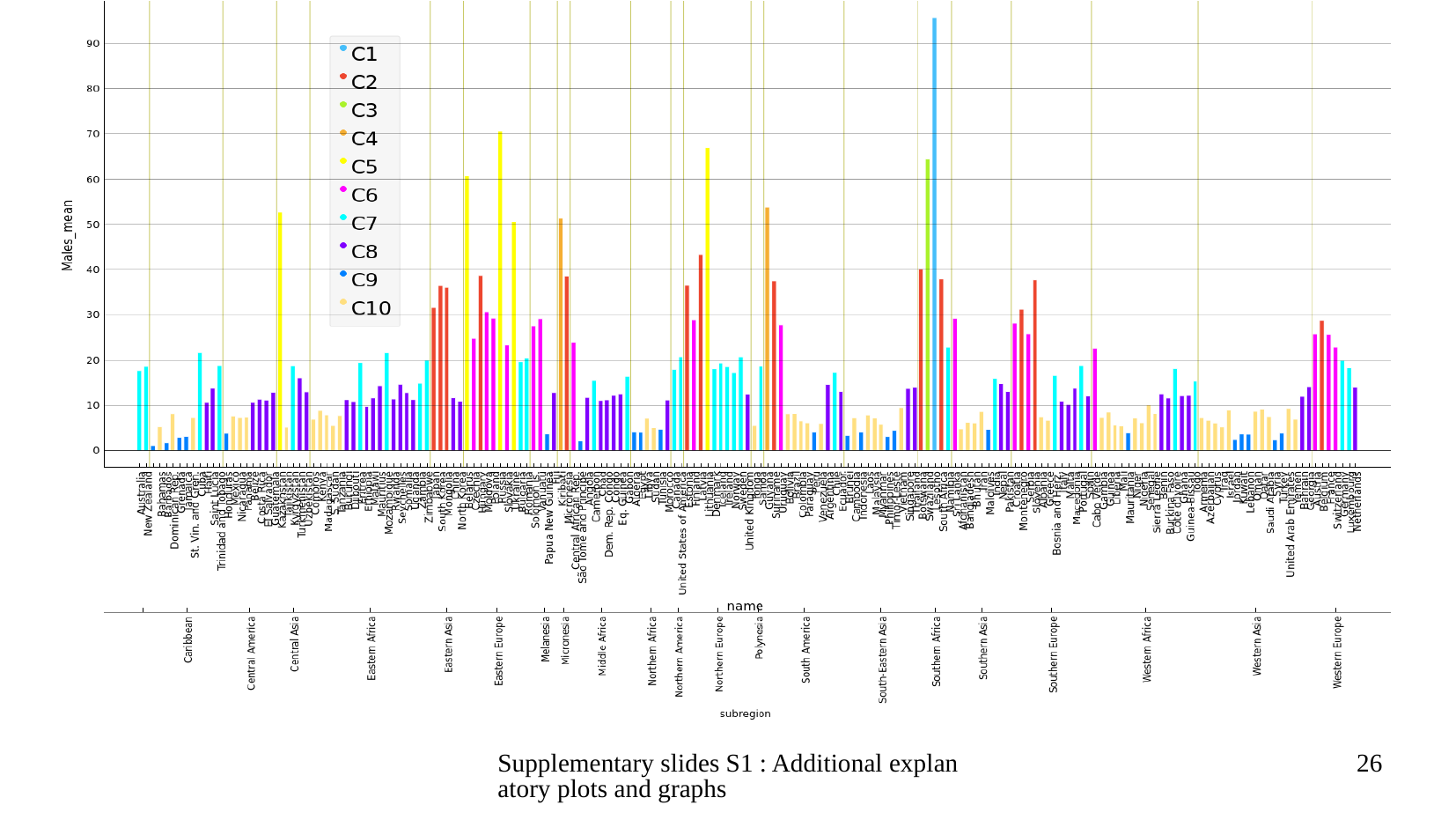

Supplementary slides S1 : Additional explanatory plots and graphs
26

## Slide 27
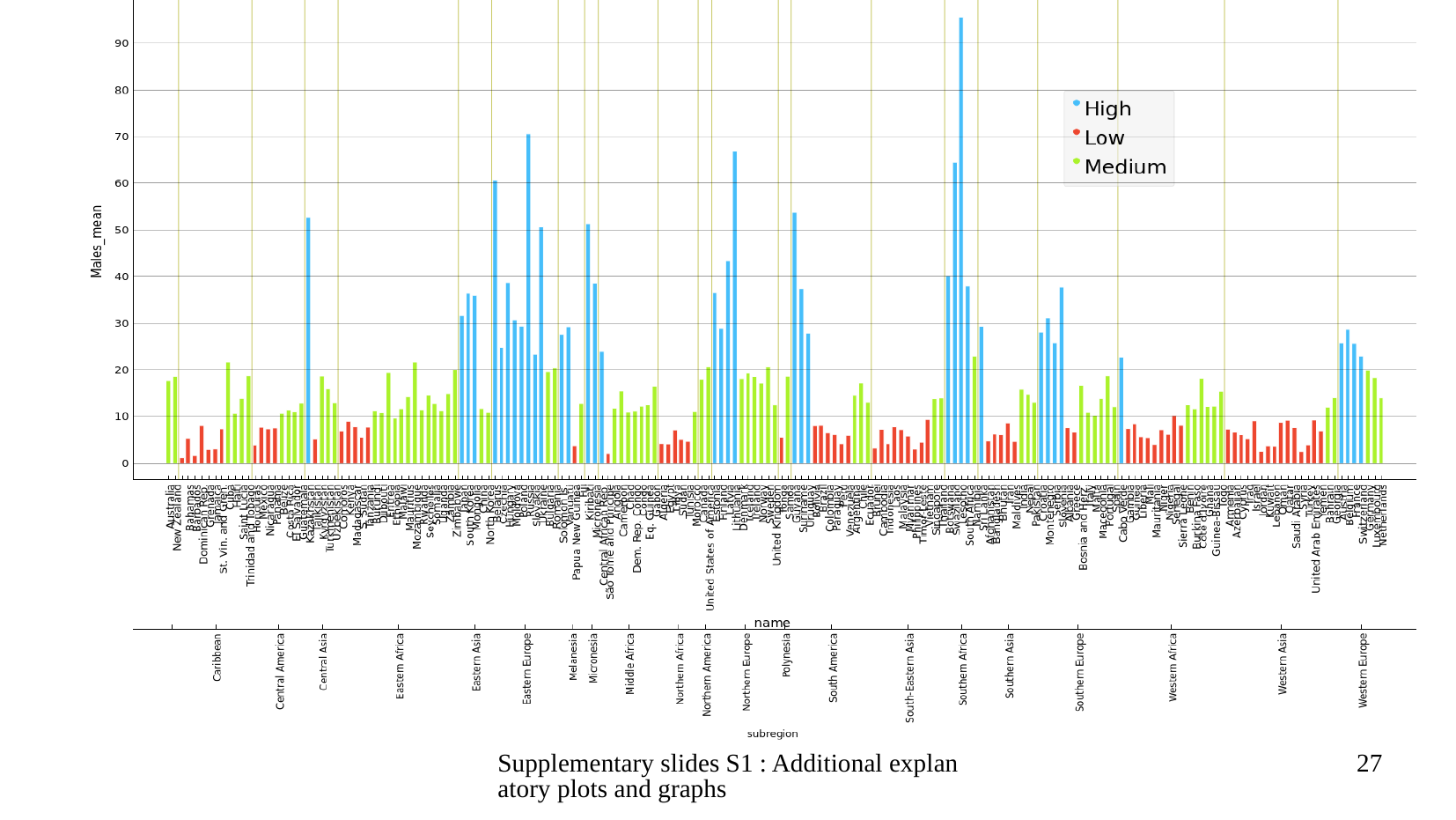

Supplementary slides S1 : Additional explanatory plots and graphs
27

## Slide 28
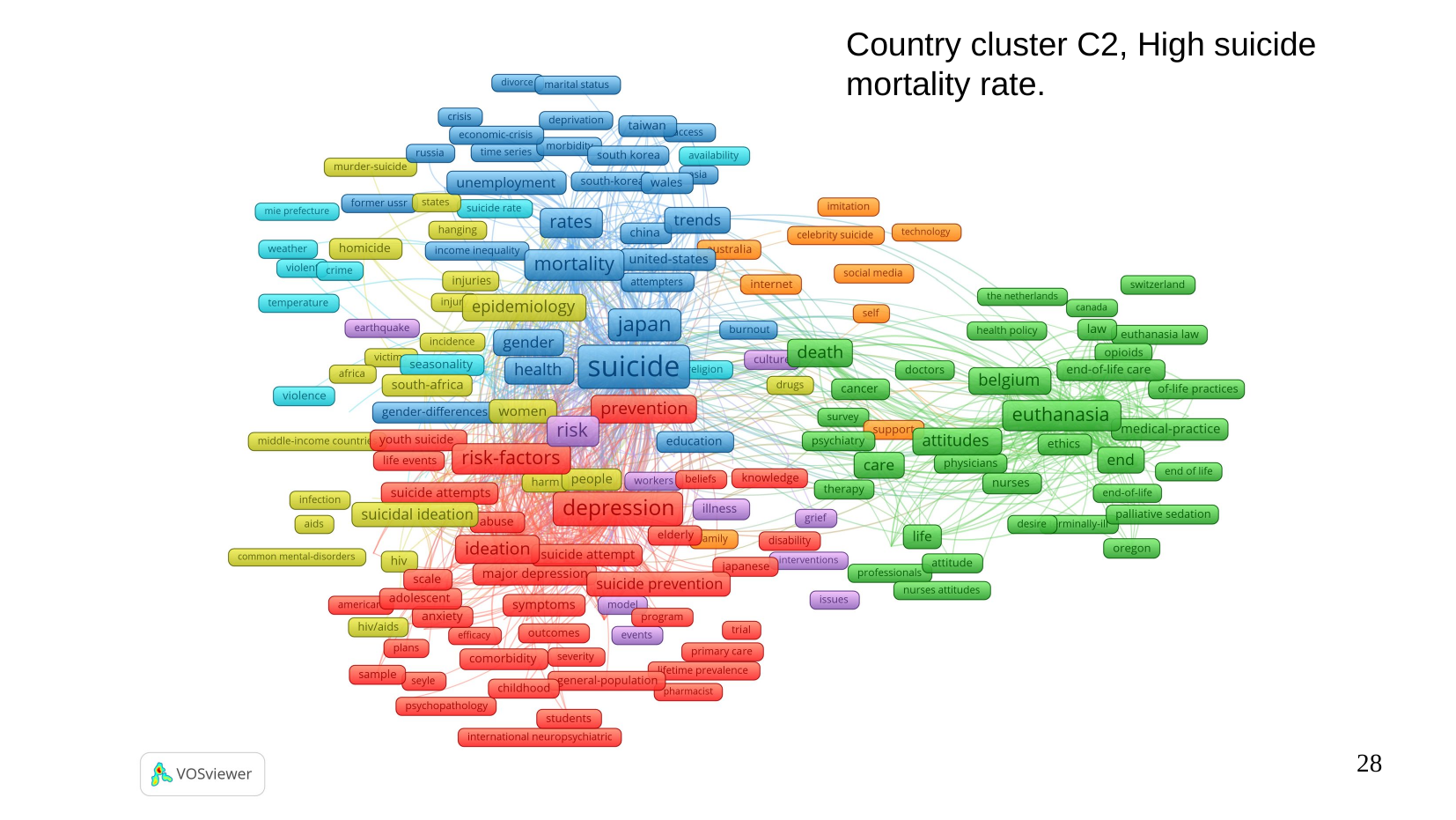

Country cluster C2, High suicide mortality rate.
Supplementary slides S1 : Additional explanatory plots and graphs
28

## Slide 29
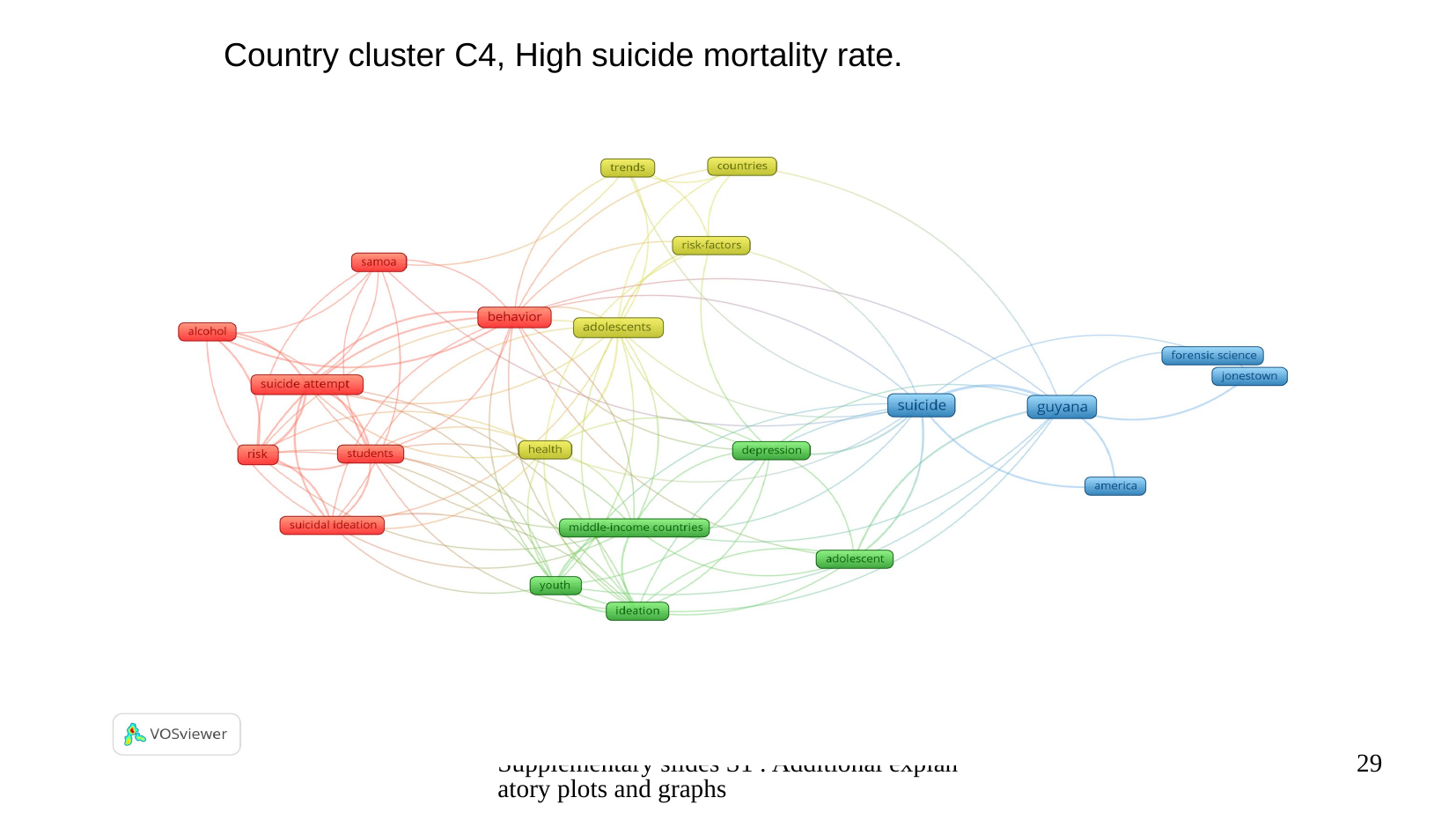

Country cluster C4, High suicide mortality rate.
Supplementary slides S1 : Additional explanatory plots and graphs
29

## Slide 30
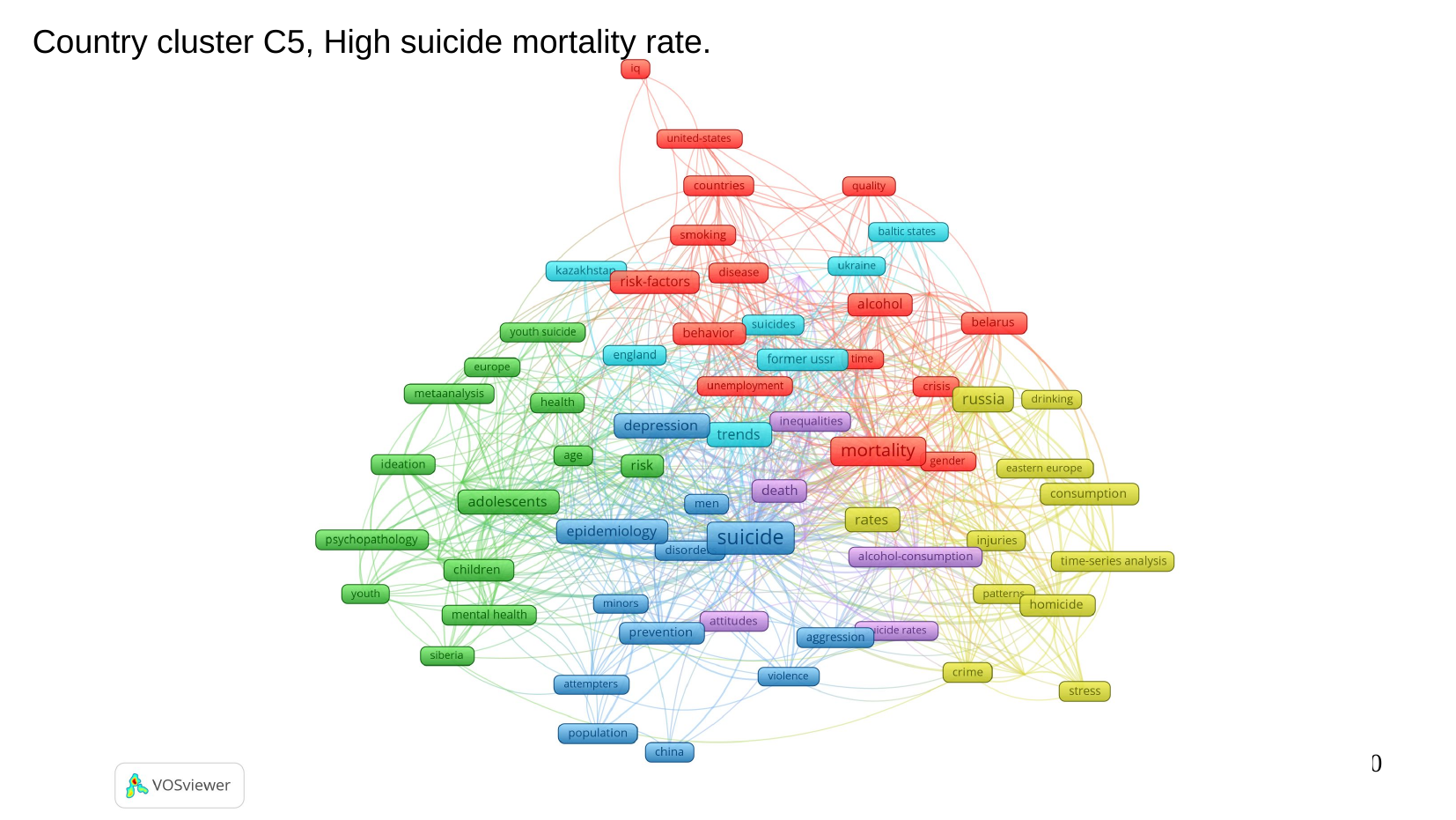

Country cluster C5, High suicide mortality rate.
Supplementary slides S1 : Additional explanatory plots and graphs
30

## Slide 31
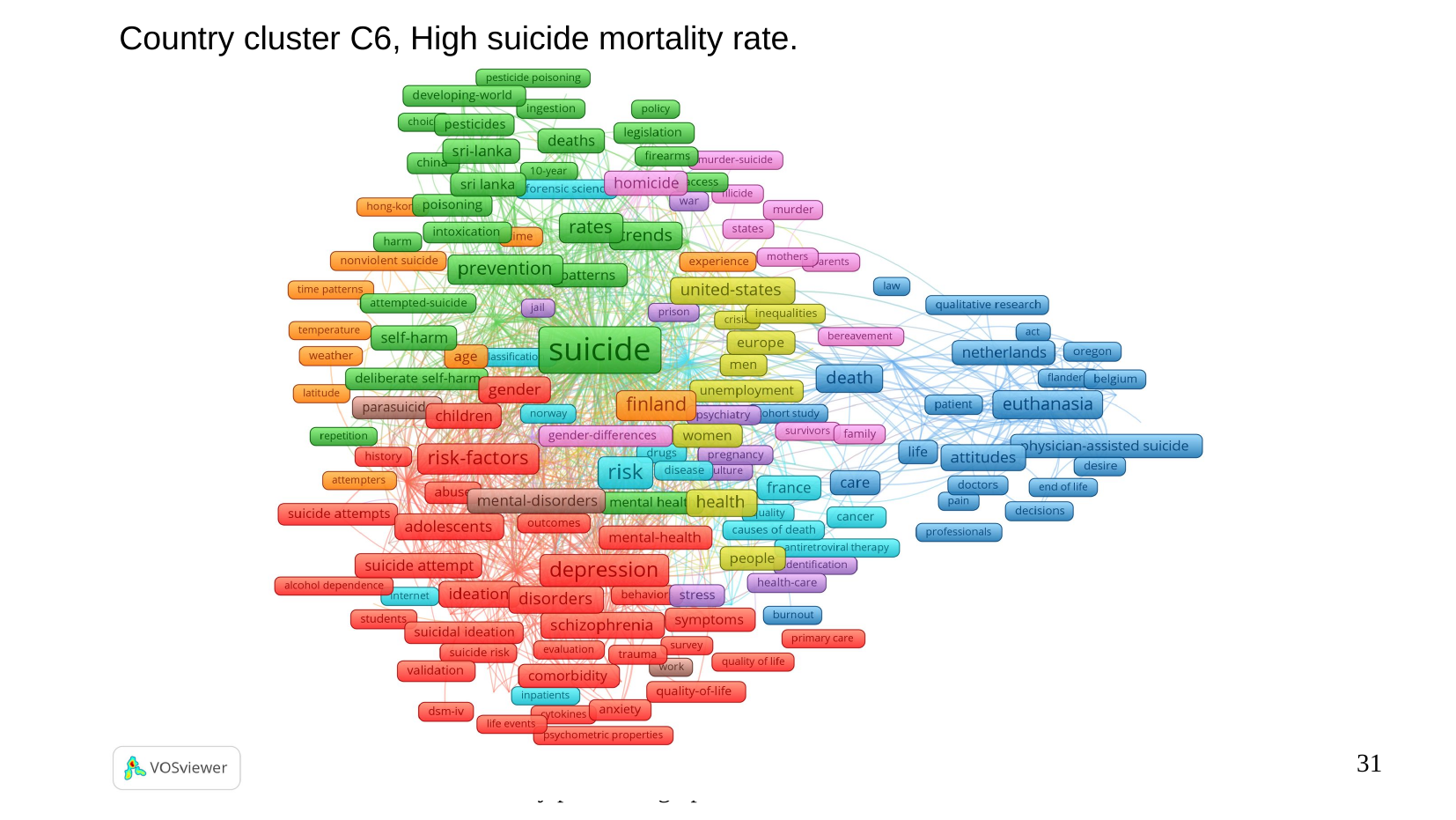

Country cluster C6, High suicide mortality rate.
Supplementary slides S1 : Additional explanatory plots and graphs
31

## Slide 32
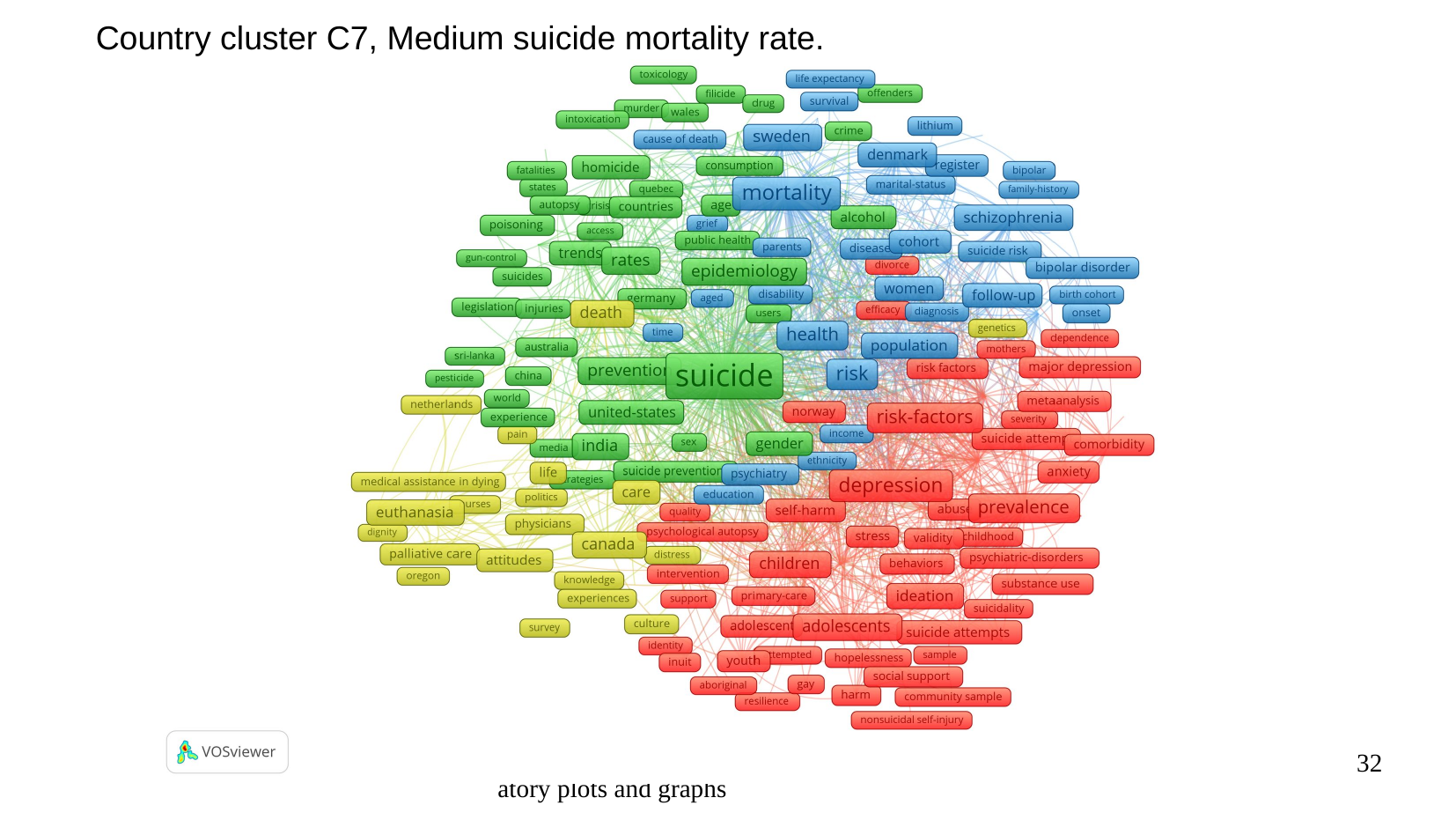

Country cluster C7, Medium suicide mortality rate.
Supplementary slides S1 : Additional explanatory plots and graphs
32

## Slide 33
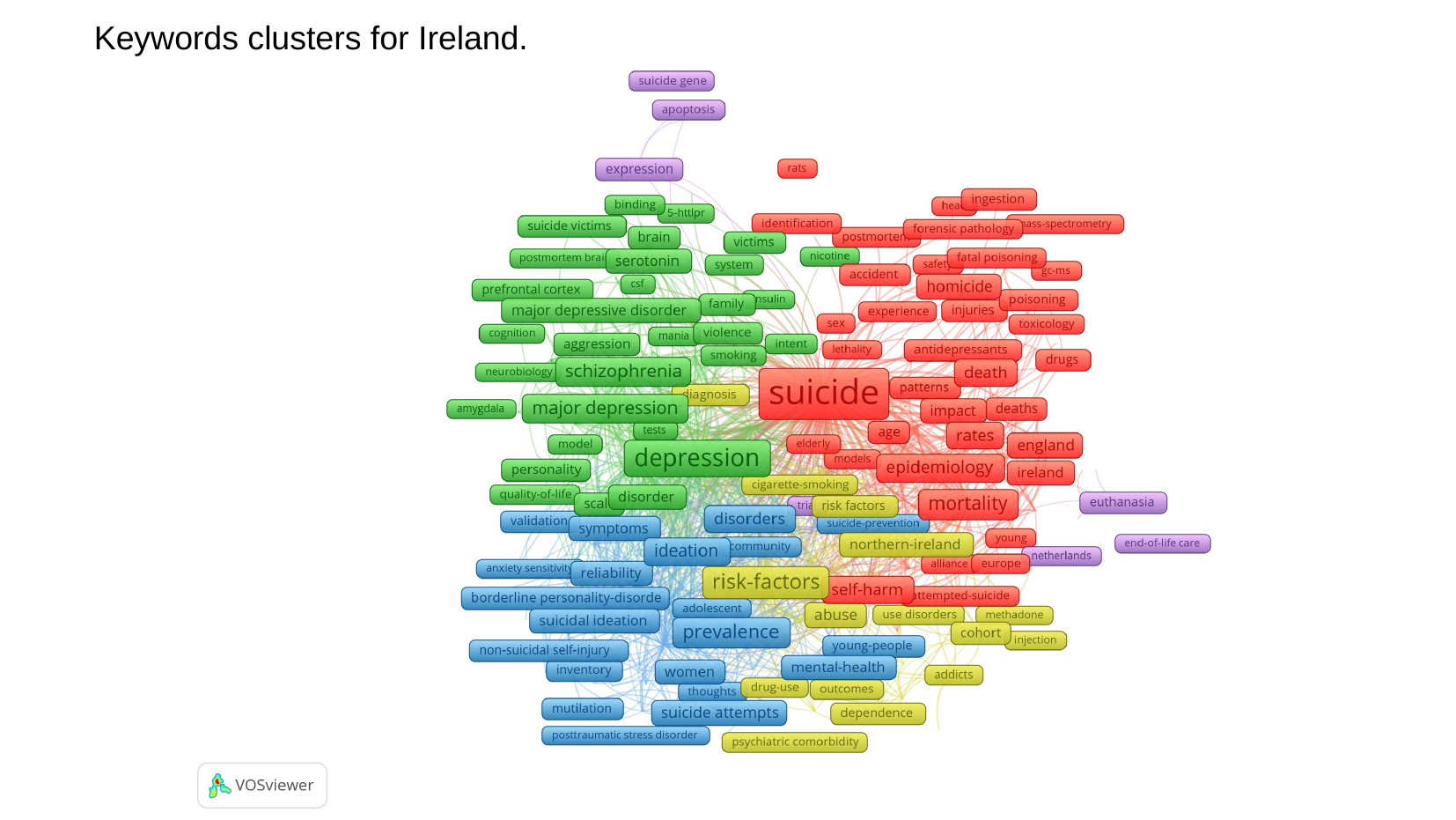

Keywords clusters for Ireland.
Supplementary slides S1 : Additional explanatory plots and graphs
33

## Slide 34
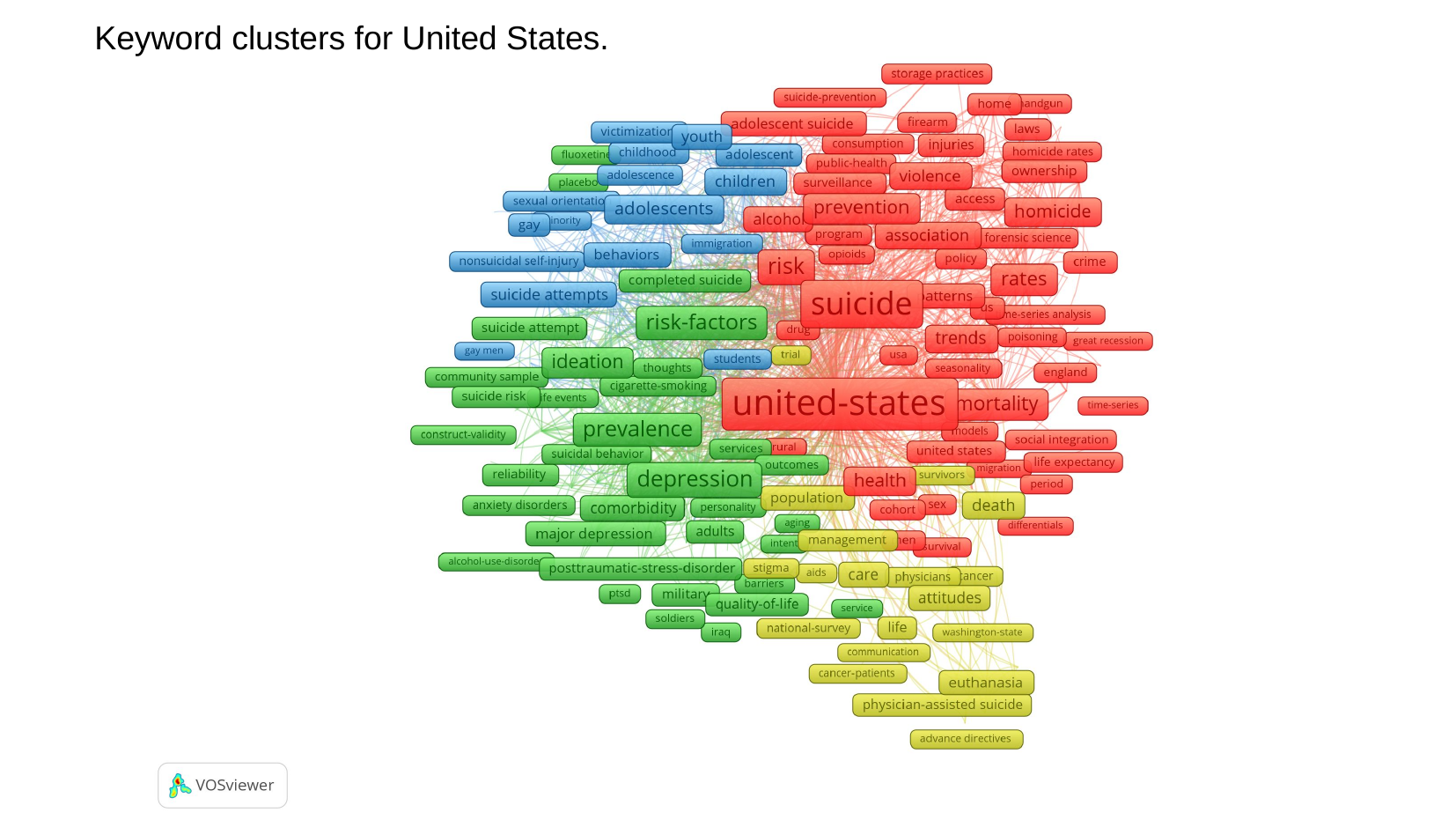

Keyword clusters for United States.
Supplementary slides S1 : Additional explanatory plots and graphs
34

## Slide 35
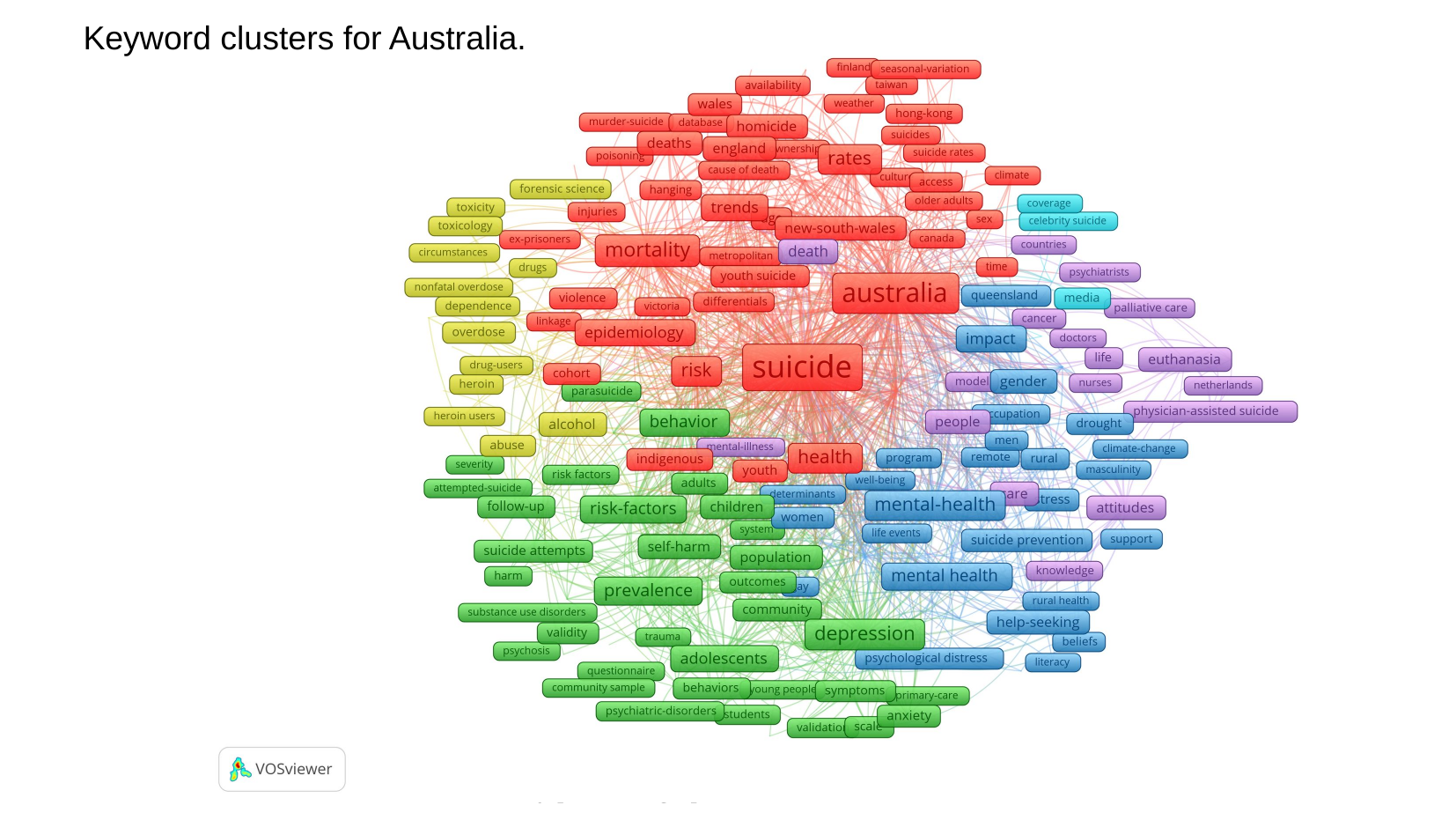

Keyword clusters for Australia.
Supplementary slides S1 : Additional explanatory plots and graphs
35

## Slide 36
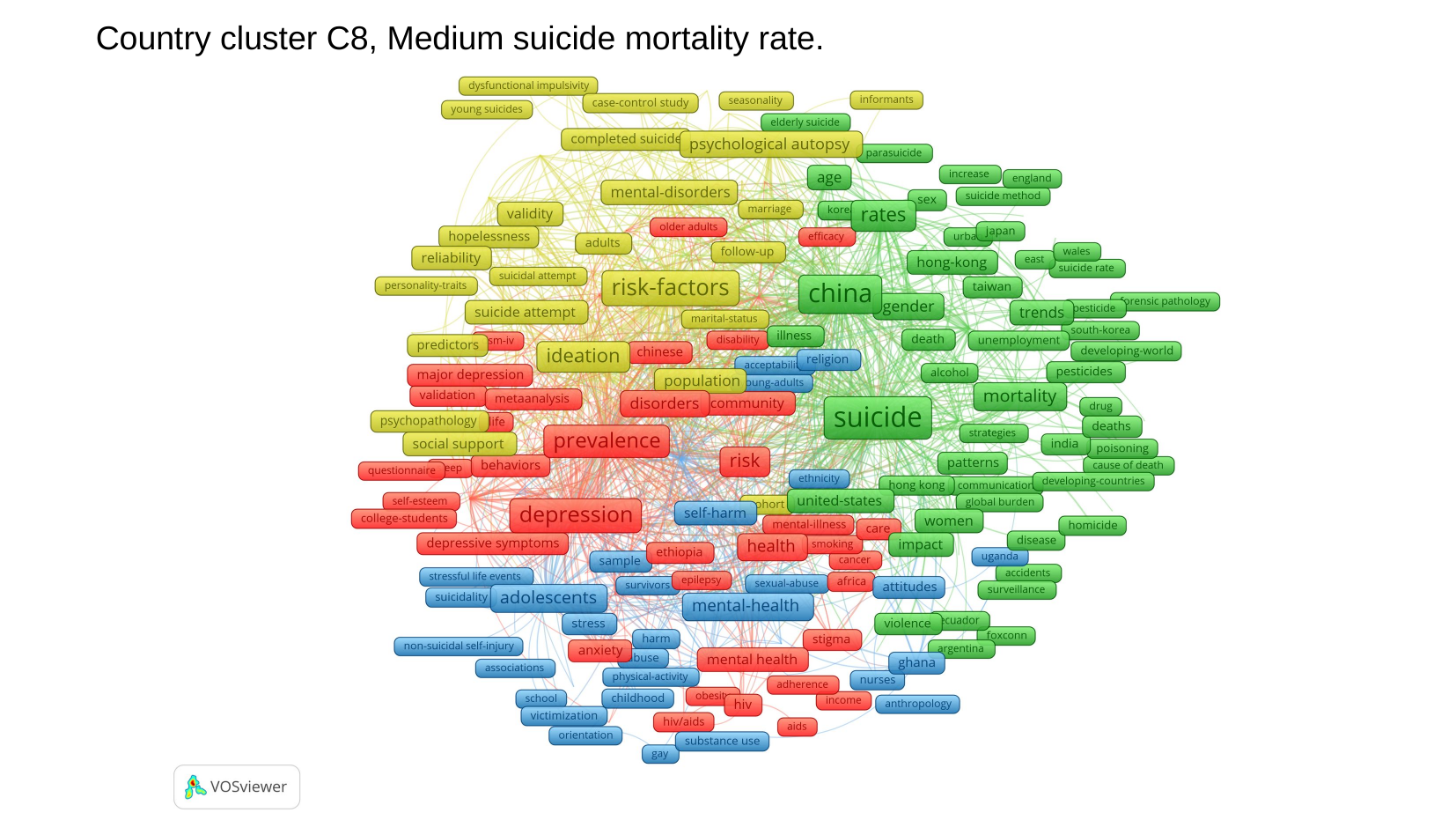

Country cluster C8, Medium suicide mortality rate.
Supplementary slides S1 : Additional explanatory plots and graphs
36

## Slide 37
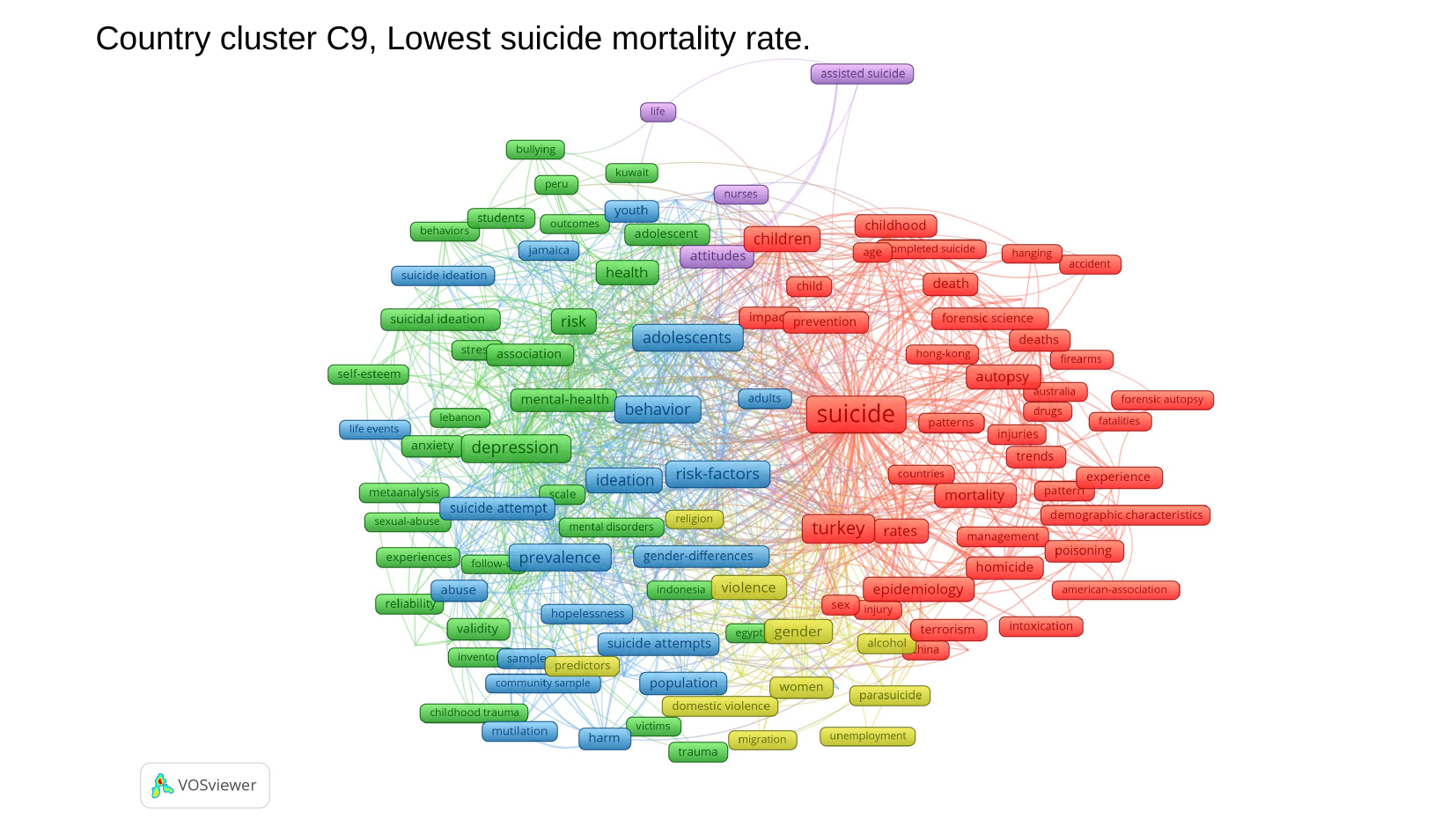

Country cluster C9, Lowest suicide mortality rate.
Supplementary slides S1 : Additional explanatory plots and graphs
37

## Slide 38
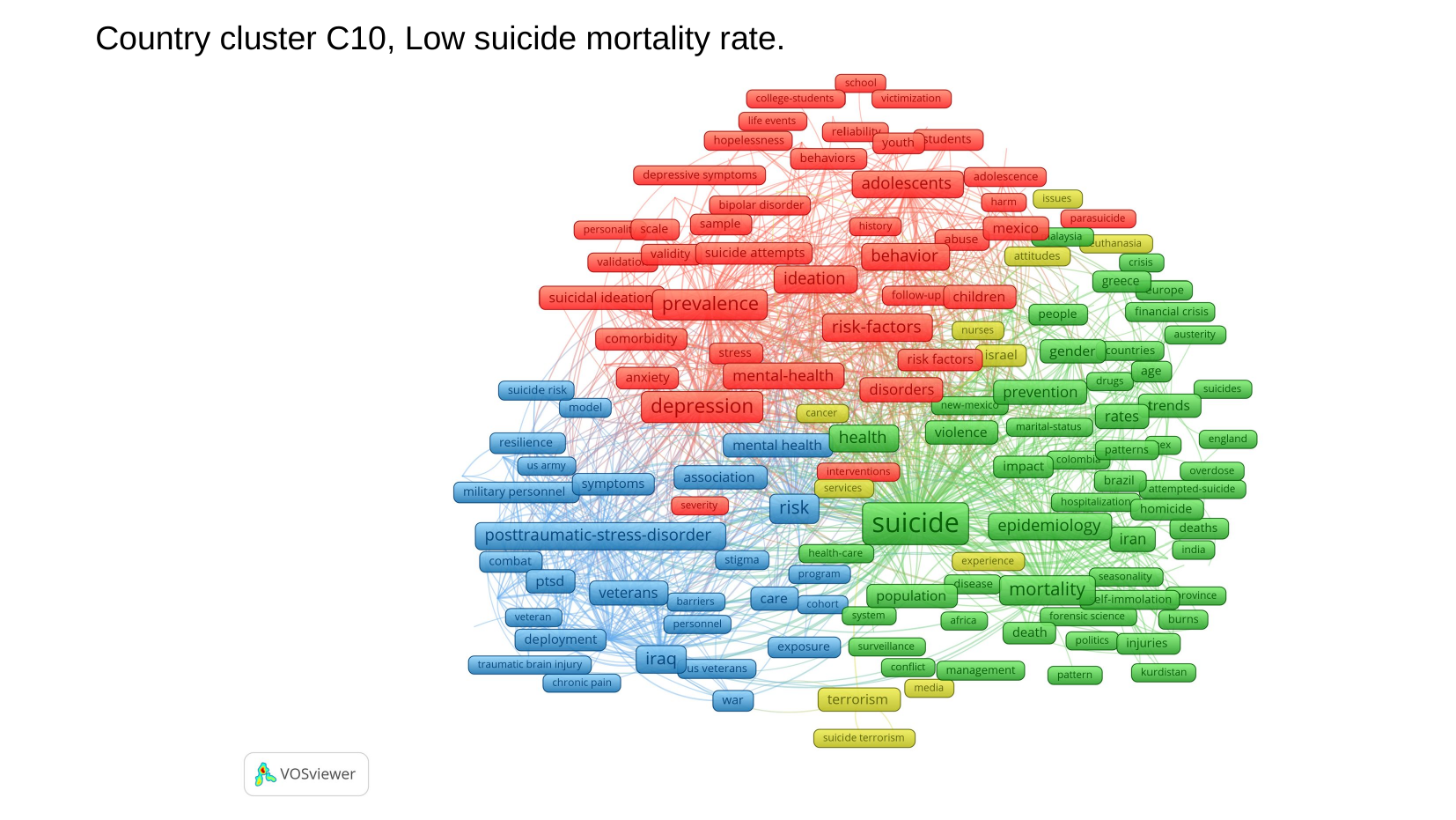

Country cluster C10, Low suicide mortality rate.
Supplementary slides S1 : Additional explanatory plots and graphs
38

## Slide 39
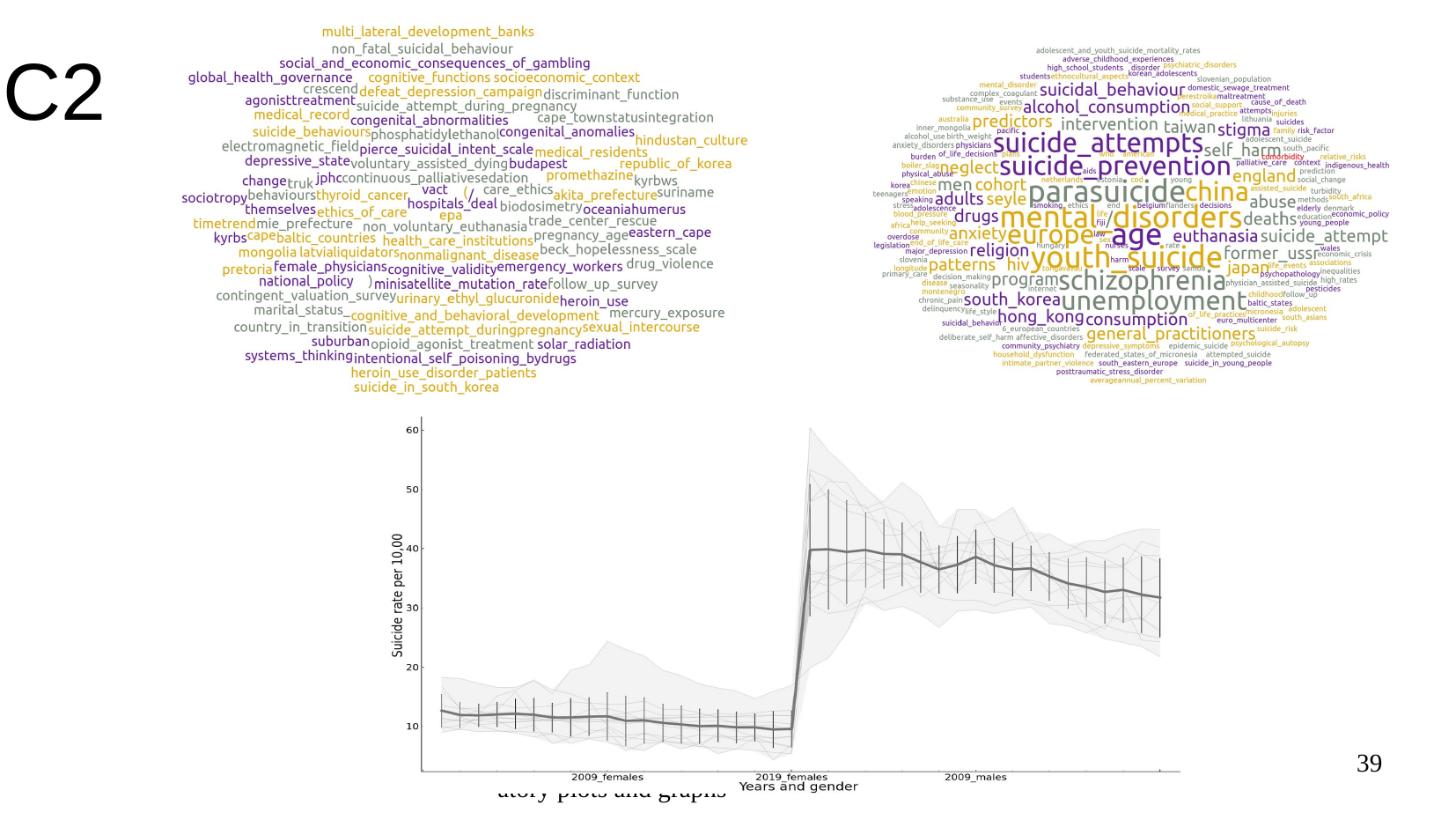

# C2
Supplementary slides S1 : Additional explanatory plots and graphs
39

## Slide 40
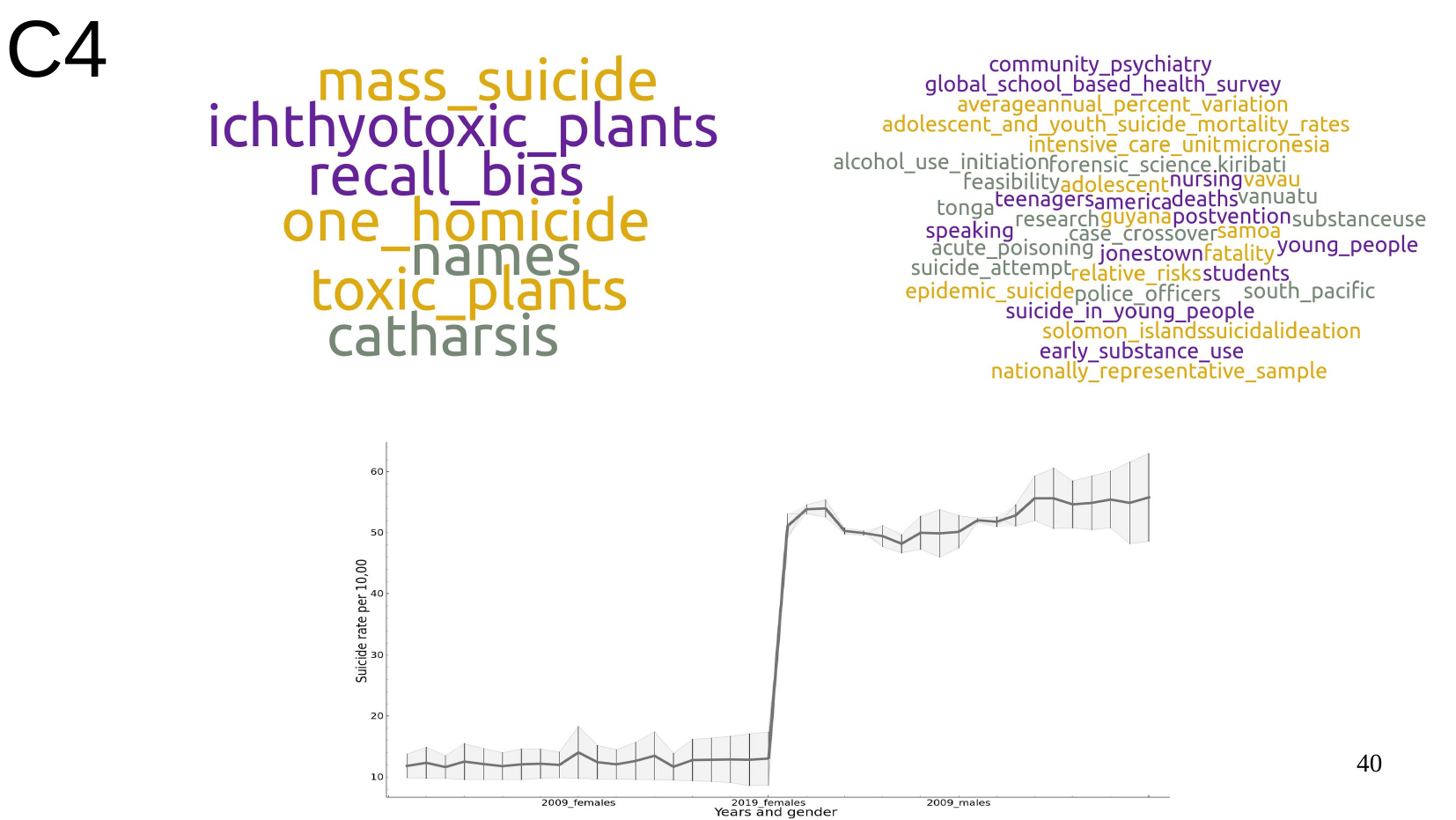

# C4
Supplementary slides S1 : Additional explanatory plots and graphs
40

## Slide 41
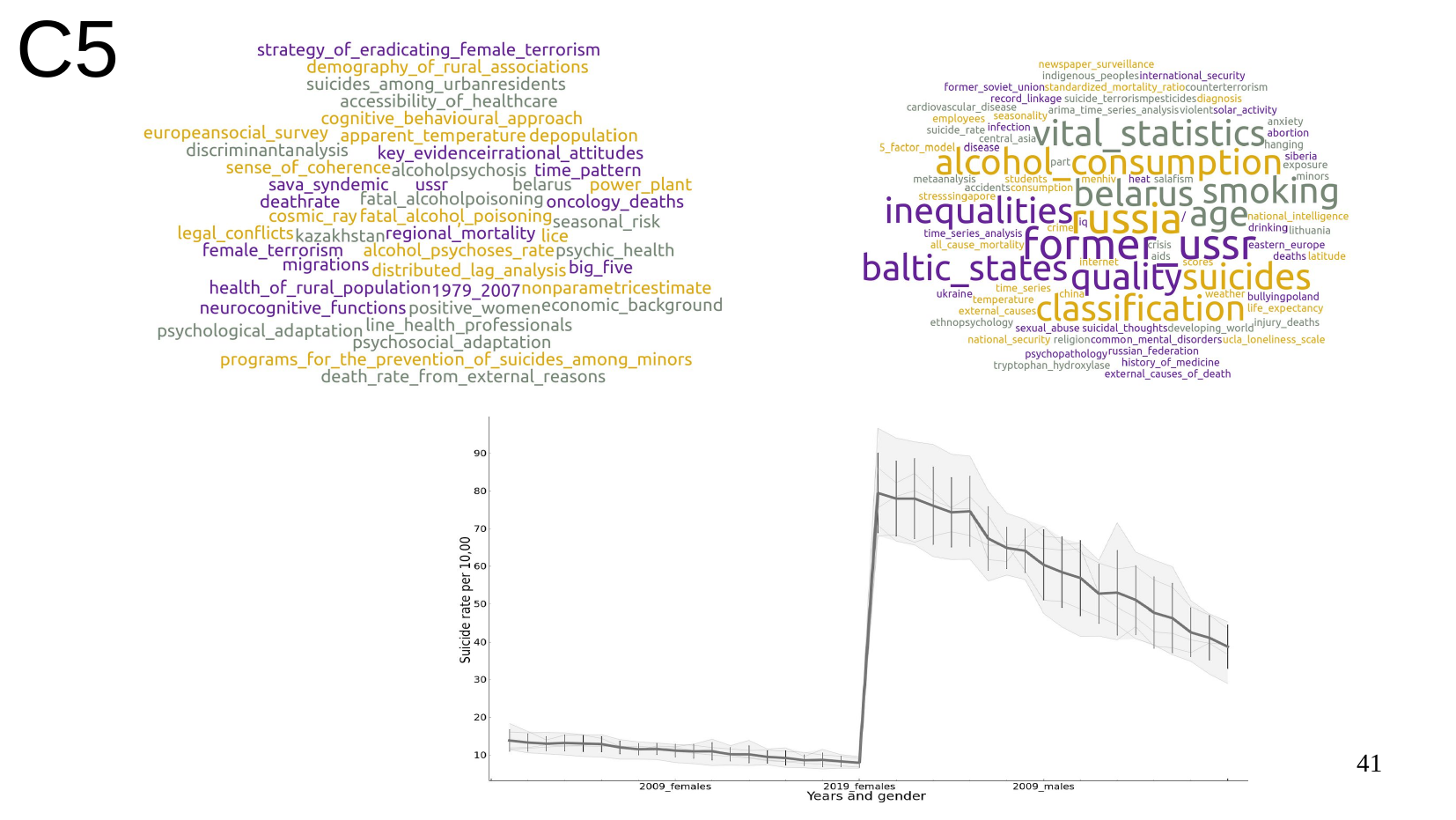

# C5
Supplementary slides S1 : Additional explanatory plots and graphs
41

## Slide 42
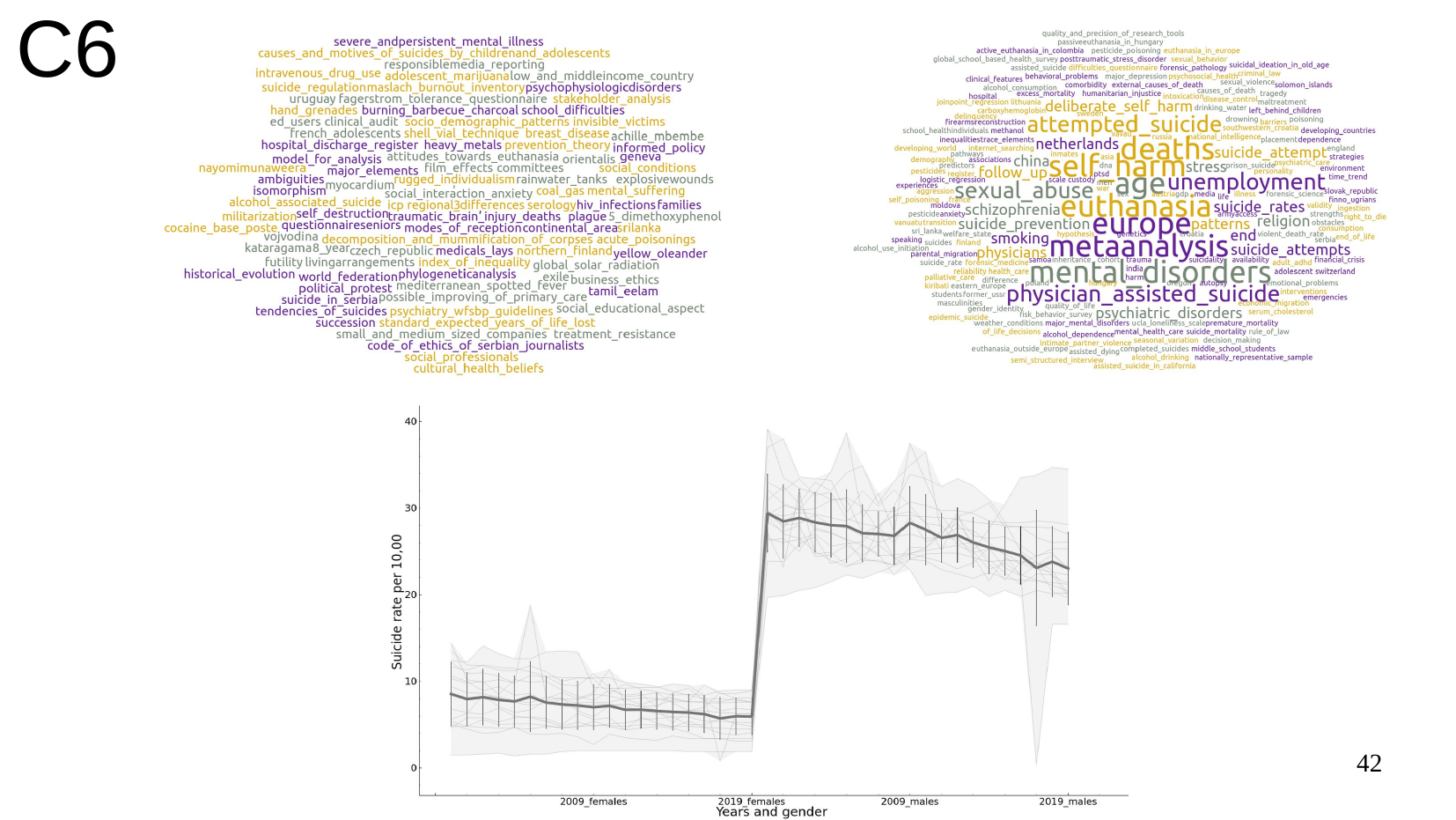

# C6
Supplementary slides S1 : Additional explanatory plots and graphs
42

## Slide 43
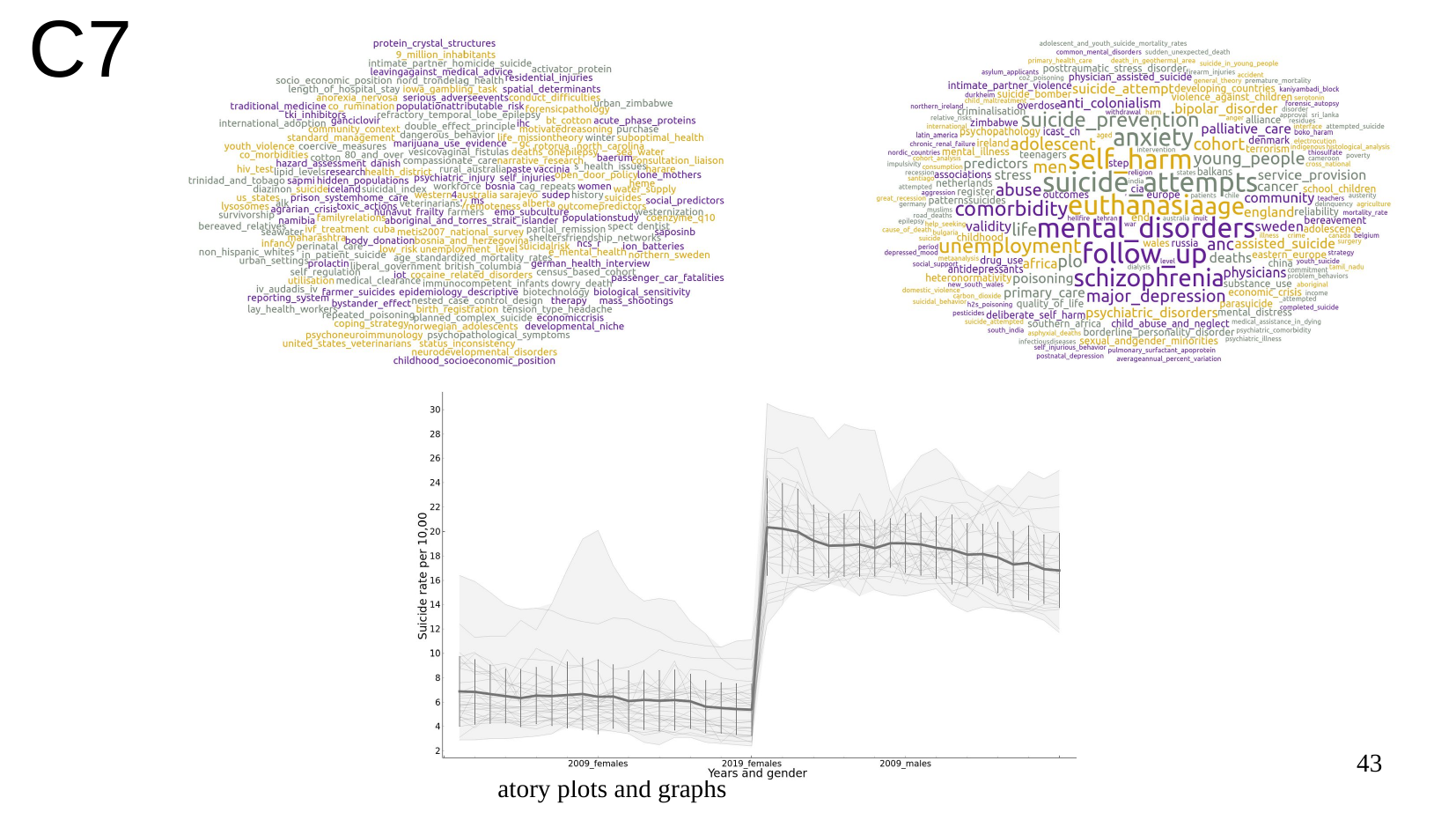

# C7
Supplementary slides S1 : Additional explanatory plots and graphs
43

## Slide 44
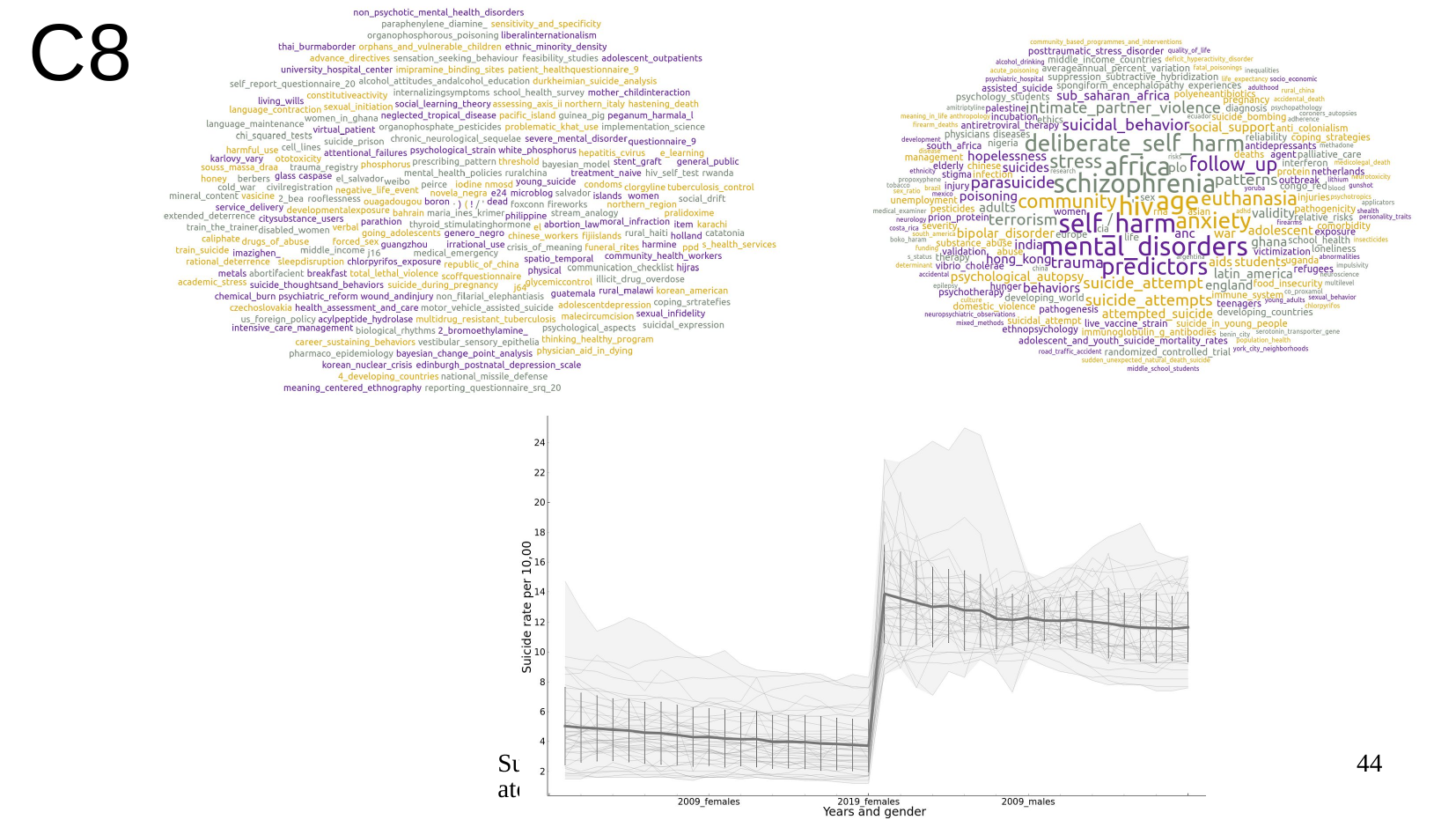

# C8
Supplementary slides S1 : Additional explanatory plots and graphs
44

## Slide 45
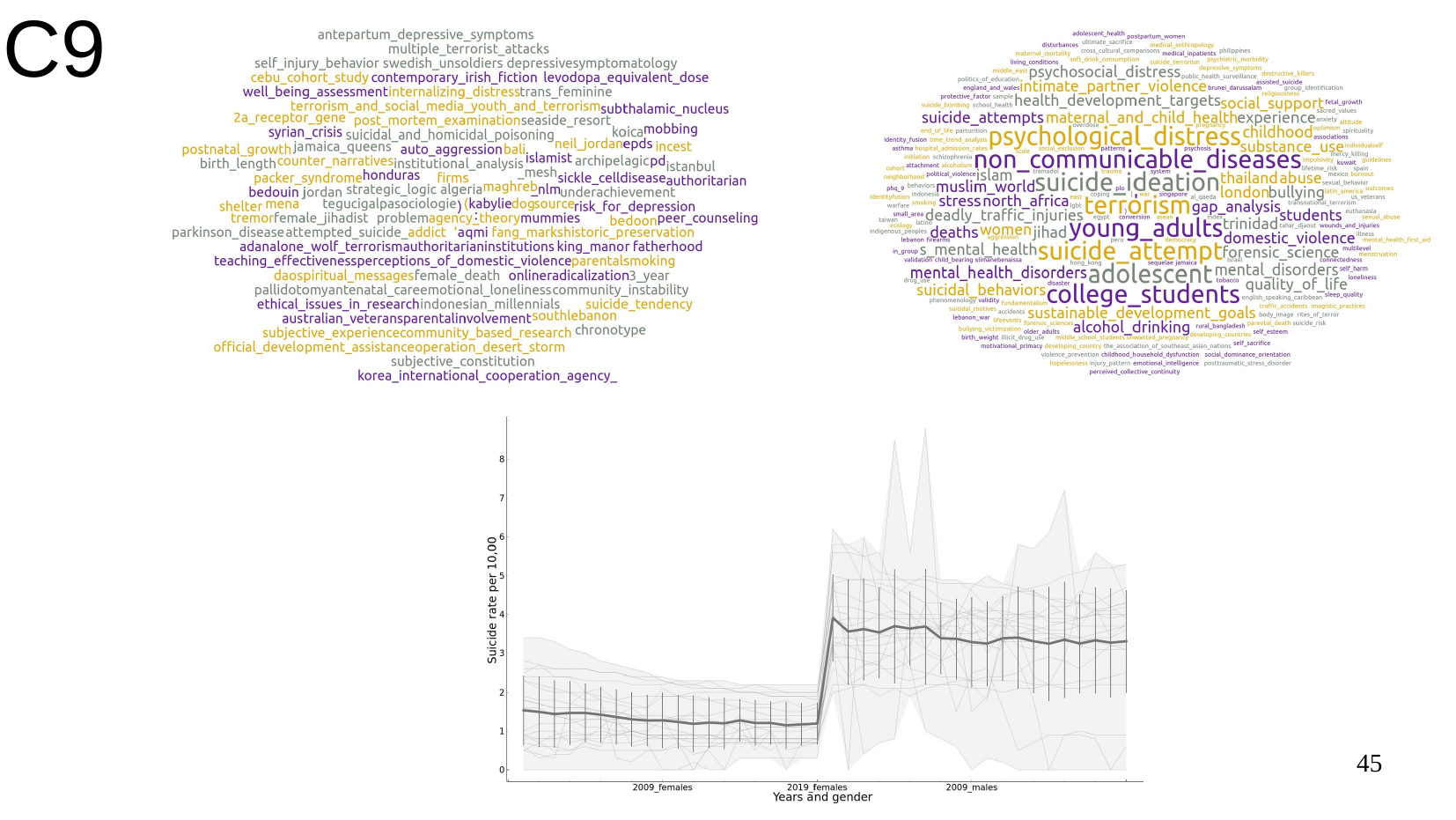

# C9
Supplementary slides S1 : Additional explanatory plots and graphs
45

## Slide 46
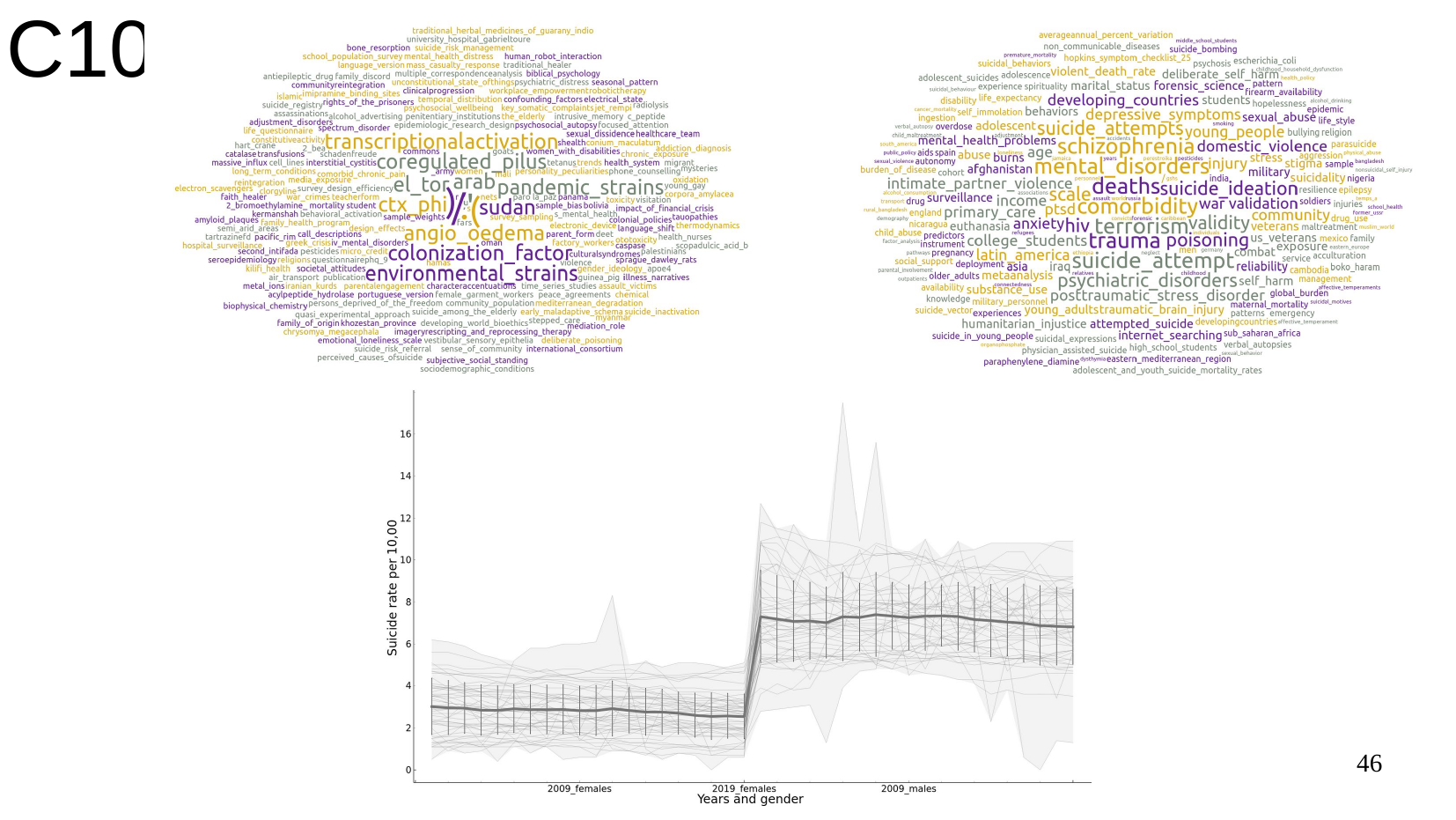

# C10
Supplementary slides S1 : Additional explanatory plots and graphs
46

## Slide 47
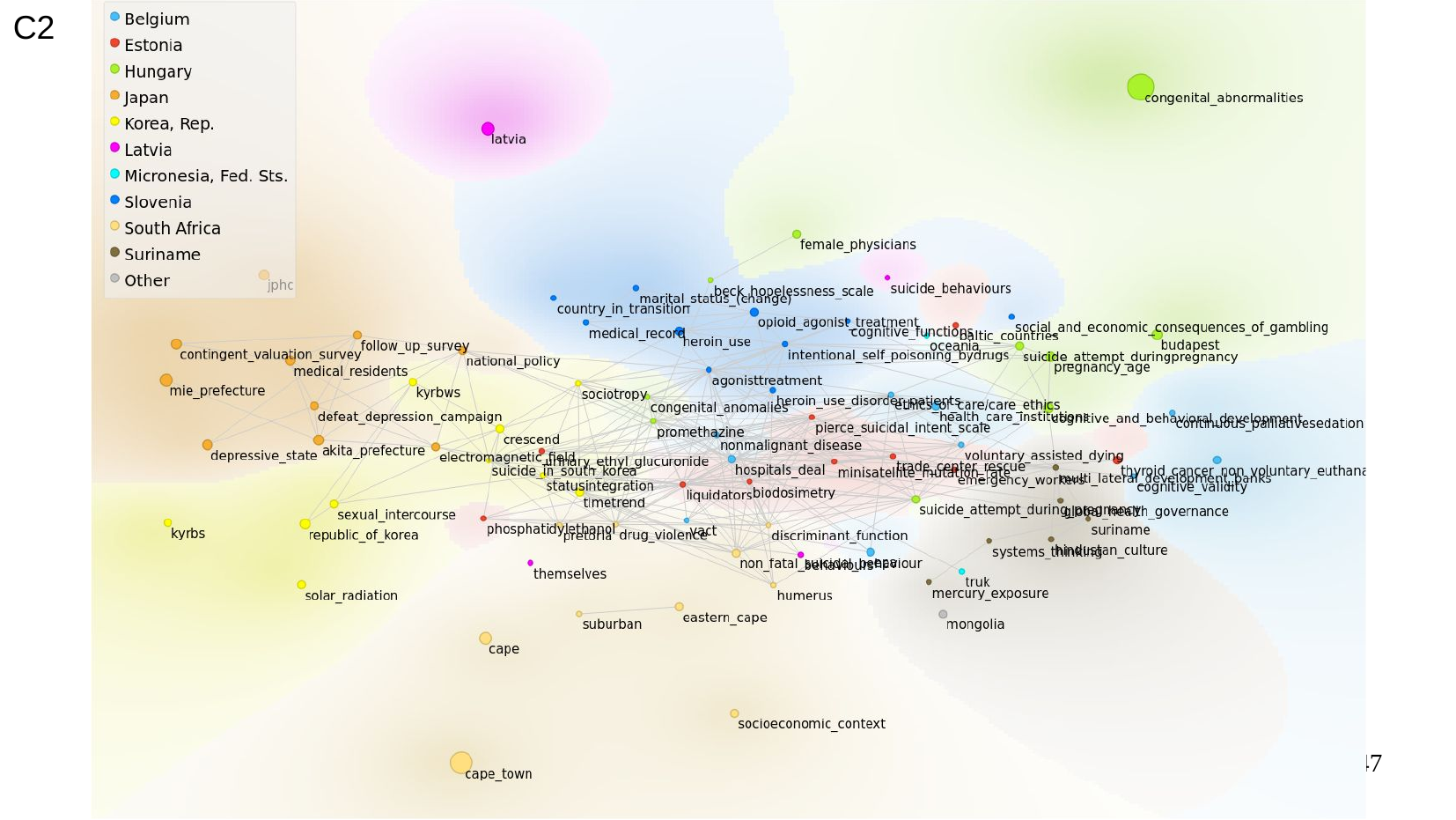

C2
Supplementary slides S1 : Additional explanatory plots and graphs
47

## Slide 48
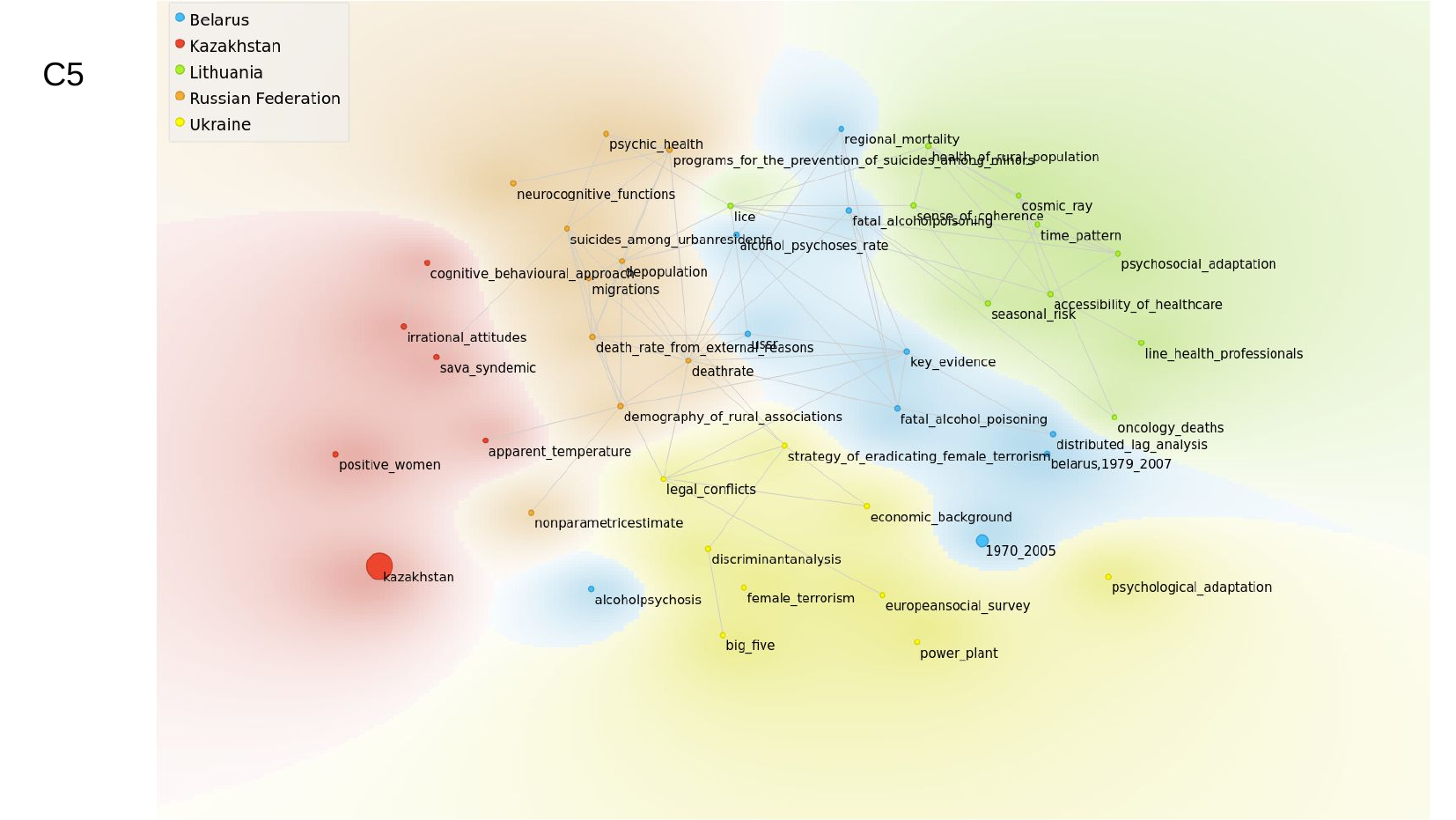

C5
Supplementary slides S1 : Additional explanatory plots and graphs
48

## Slide 49
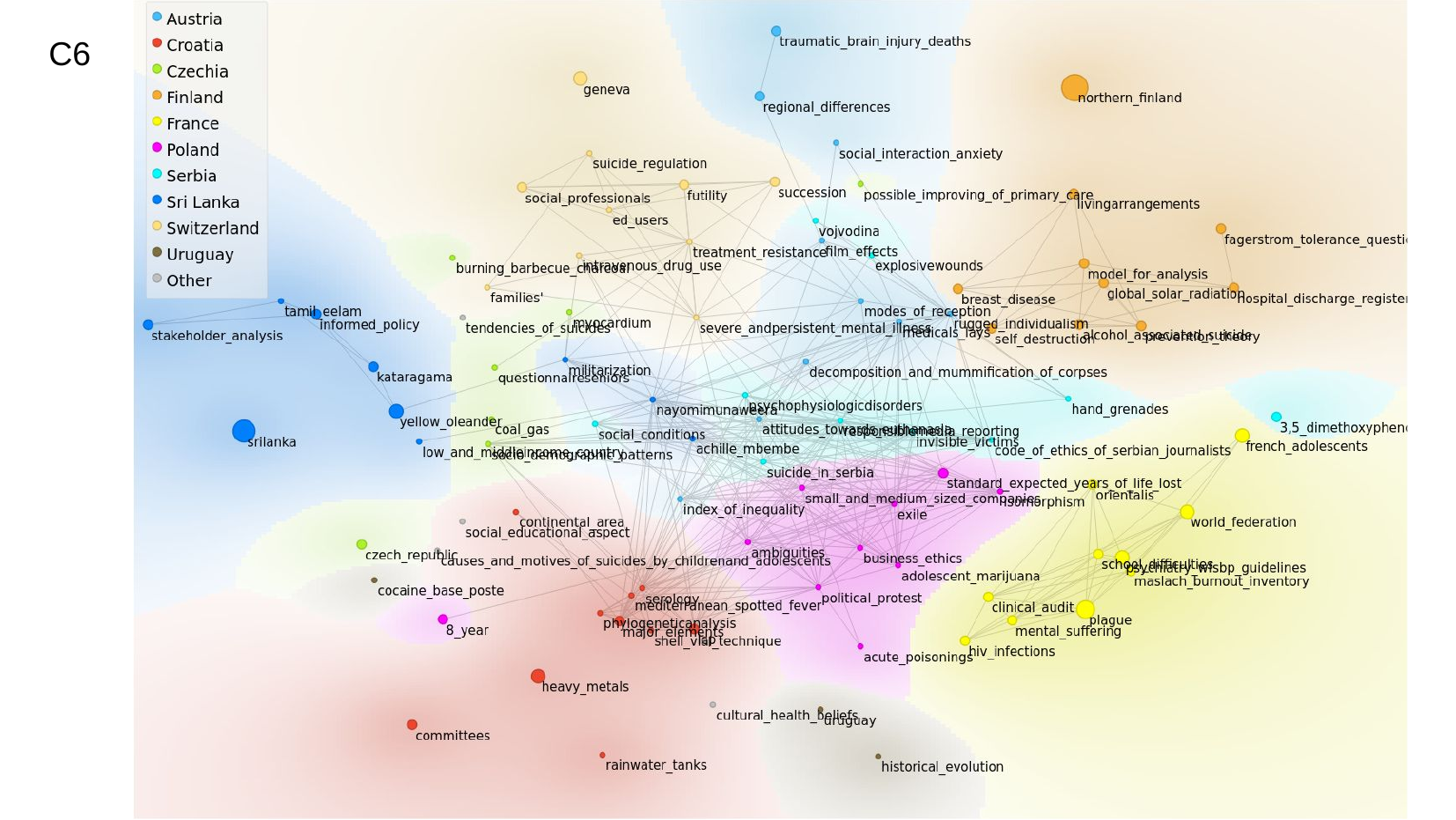

C6
Supplementary slides S1 : Additional explanatory plots and graphs
49
