# Supplementary figures and images for: Global Suicide Mortality Rates (2000–2019): Clustering, Themes, and Causes Analyzed through Machine Learning and Bibliographic Data
Source: Int J Environ Res Public Health. 2024 Sep 10;21(9):1202. doi: 10.3390/ijerph21091202 (PMC11431541; doi:10.3390/ijerph21091202)

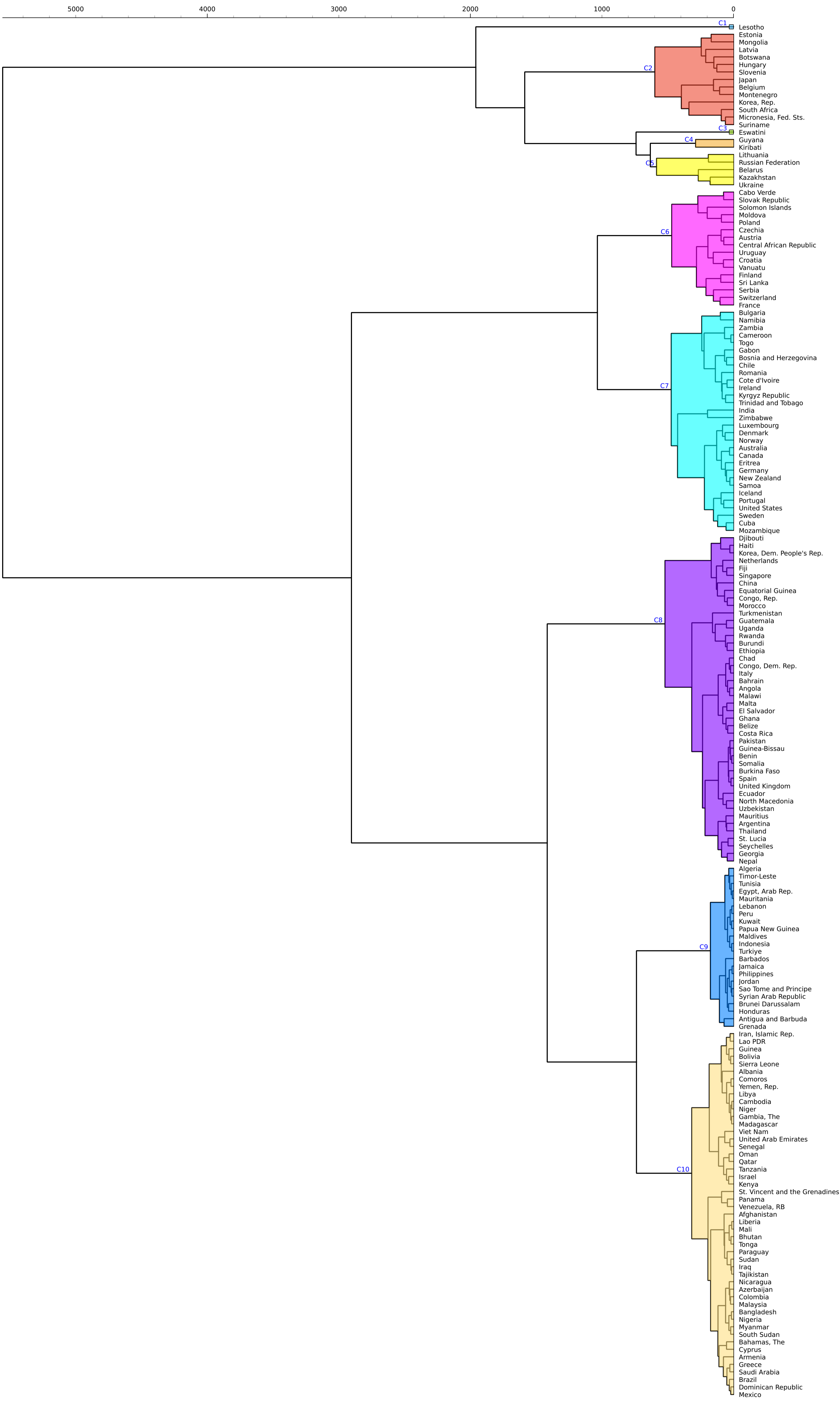

Supplement: Supplementary file 1 [file ijerph-21-01202-s001.zip › ijerph-3110525-supplementary/SUPPLEMENTARY/Supplementary_Figure_S1_Country_clusters.pdf]

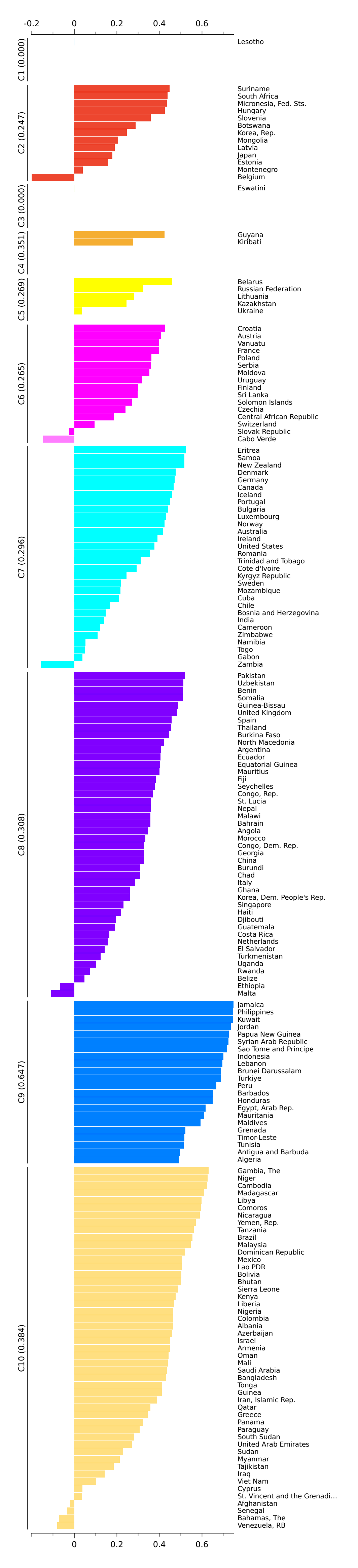

Supplement: Supplementary file 1 [file ijerph-21-01202-s001.zip › ijerph-3110525-supplementary/SUPPLEMENTARY/Supplementary_Figure_S2_Country_clusters_silhouette_plot.pdf]
